# Molecular clock REV-ERB**α** regulates cigarette smoke–induced pulmonary inflammation and epithelial-mesenchymal transition

**DOI:** 10.1172/jci.insight.145200

**Published:** 2021-06-22

**Authors:** Qixin Wang, Isaac K. Sundar, Joseph H. Lucas, Thivanka Muthumalage, Irfan Rahman

**Affiliations:** 1 Department of Environmental Medicine, School of Medicine and Dentistry, University of Rochester Medical Center, Rochester, New York, USA.; 2 Department of Internal Medicine, Division of Pulmonary, Critical Care and Sleep Medicine, University of Kansas Medical Center, Kansas City, Kansas, USA.

**Keywords:** Inflammation, Pulmonology, COPD

## Abstract

Cigarette smoke (CS) is the main etiological factor in the pathogenesis of emphysema/chronic obstructive pulmonary disease (COPD), which is associated with abnormal epithelial-mesenchymal transition (EMT). Previously, we have shown an association among circadian rhythms, CS-induced lung inflammation, and nuclear heme receptor α (REV-ERBα), acting as an antiinflammatory target in both pulmonary epithelial cells and fibroblasts. We hypothesized that molecular clock REV-ERBα plays an important role in CS-induced circadian dysfunction and EMT alteration. C57BL/6J WT and *REV-ERB*α heterozygous (Het) and –KO mice were exposed to CS for 30 days (subchronic) and 4 months (chronic), and WT mice were exposed to CS for 10 days with or without REV-ERBα agonist (SR9009) administration. Subchronic/chronic CS exposure caused circadian disruption and dysregulated EMT in the lungs of WT and *REV-ERB*α*–*KO mice; both circadian and EMT dysregulation were exaggerated in the *REV-ERB*α*–*KO condition. REV-ERBα agonist, SR9009 treatment reduced acute CS-induced inflammatory response and abnormal EMT in the lungs. Moreover, REV-ERBα agonist (GSK4112) inhibited TGF-β/CS–induced fibroblast differentiation in human fetal lung fibroblast 1 (HFL-1). Thus, CS-induced circadian gene alterations and EMT activation are mediated through a *Rev-erb*α–dependent mechanism, which suggests activation of REV-ERBα as a novel therapeutic approach for smoking-induced chronic inflammatory lung diseases.

## Graphical abstract

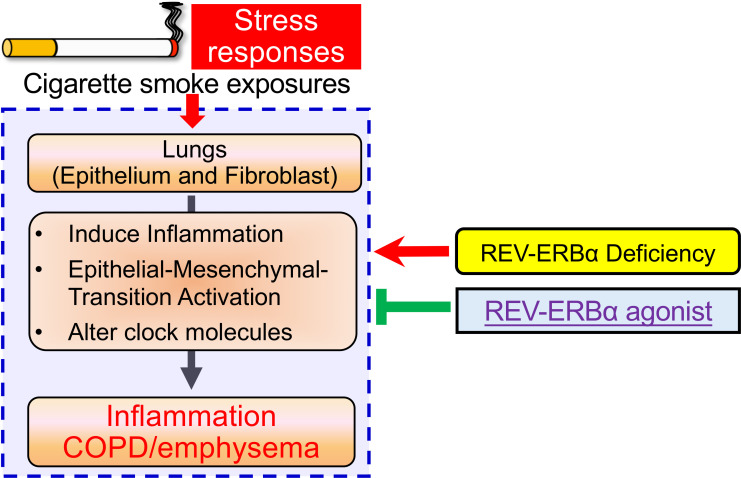

## Introduction

Chronic obstructive pulmonary disease (COPD) is one of the leading causes of death worldwide; it affects more than 10% of the global population (1). There are limited therapeutic strategies currently available, and the comprehensive treatment mechanisms remain unclear (2). COPD is a chronic lung disease resulting from exposure to environmental pollutants/toxicants, noxious gases, and cigarette smoke (CS). CS is a significant risk factor that causes COPD. Our previous reports showed that short-term CS exposure (acute and subchronic) causes inflammation/injury in mouse lungs, and chronic CS exposure results in emphysema/COPD, which could be exacerbated by influenza A virus infection (3–5).

Epithelial-mesenchymal transition (EMT) has been studied previously as a primary mechanism of COPD (6). Downregulation of epithelial phenotypes (E-cadherin, ZO-1, and occludin), increased mesenchymal phenotypes (vimentin, α-smooth muscle actin [αSMA], and fibronectin), and extracellular matrix remodeling, as well as altered barrier function, are all associated with CS-induced COPD phenotypes (7, 8). Additionally, bronchial epithelium exposed to CS showed decreased barrier function with a reduced abundance of adherence junction proteins and increased mesenchymal markers (9). Intriguingly, transcriptions of most EMT genes are regulated by E-box, which also regulates circadian clock genes (10–12). Previous research has shown that circadian clock molecules play an essential role in lung injury and chronic inflammatory lung diseases (11, 13, 14). However, there is no information available on the role of circadian clock in modulating EMT phenotypes in COPD and its exacerbations.

Circadian oscillation is a fundamental biological process occurring within various organs, such as the heart, lung, liver, and brain (12, 15). The circadian clocks in different organs are driven by a similar core molecular feedback loop. The circadian locomotor output cycles kaput (CLOCK)/brain and muscle ARNT-like 1 (BMAL1, also known as *Arntl*) heterodimer binds to E-box and promotes the transcription/translation of either core clock molecules or downstream targets. The core clock molecules include retinoid acid receptor–related orphan receptors (RORα, RORβ, and RORγ), the nuclear heme receptor α (REV-ERBα, also known as *Nr1d1*, and REV-ERBβ, also known as *Nr1d2*]), period 1/2/3 (PER1, PER2, and PER3), and cryptochrome 1/2 (CRY1 and CRY2), which form 2 different feedback loops that affect the activity of CLOCK/BMAL1 complexes (12). Heterodimers containing PERs and CRYs inhibit transcription activated by CLOCK/BMAL1 complexes. In a different loop, RORs compete with REV-ERBα/β for binding with ROR response elements (ROREs), with RORs contributing to the activation of BMAL1 transcription, whereas REV-ERBα/β inhibits it (12). Previous studies have shown that circadian clock molecules play an important role in the pathogenesis of chronic lung diseases, such as pulmonary fibrosis, COPD, and even lung cancer (13, 16–18).

Molecular clock REV-ERBα, a transcriptional inhibitor involved in circadian rhythms, can shorten the circadian period (19). Our previous studies identified a lower abundance of REV-ERBα protein in smokers and patients with COPD in comparison to nonsmokers (14). Moreover, loss of REV-ERBα worsens the inflammatory response induced by CS, whereas REV-ERBα agonist helped to reduce the inflammation (13). However, there is no research focused on how REV-ERBα modulates abnormal EMT induced by CS. In this study, we hypothesized that the loss of REV-ERBα promotes CS-induced EMT in mouse lungs and that treatment with REV-ERBα agonist attenuates both lung inflammation and EMT.

## Results

### Activation of EMT in the lungs of smokers compared with healthy controls.

**Figure 1:**
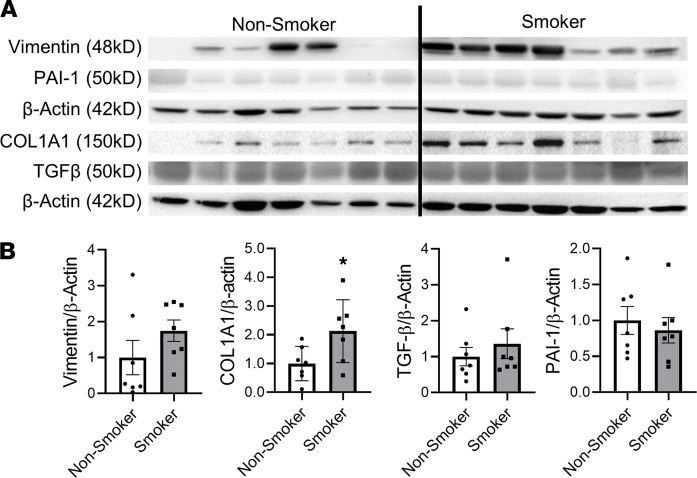
Mesenchymal markers increased in smokers compared with nonsmokers. Lungs from smokers and nonsmokers were homogenized and probed with epithelial-mesenchymal transition (EMT) markers, and protein abundance of different markers were analyzed by Western blotting. Representative blot images of target proteins (vimentin, COL1A1, TGF-β, and PAI-1) were shown in **A**. Densitometry is used for fold change of specific protein targets (**B**), and actin was used as endogenous controls for normalization. All the bands of different targets were probed on the same membrane. Vimentin and PAI-1 were probed in the same membrane, and COL1A1 and TGF-β were probed in the same membrane. Data are shown as mean ± SEM (*n* = 7, *P < 0.05, paired Student’s *t* test).

It has been shown that CS induces EMT in lung epithelial cells in culture and the lungs of smokers compared with health control. As shown in Figure 1, we observed a significant increase in the abundance of type-1 collagen (COL1A1) in smokers compared with nonsmokers as well as a nonsignificant increasing trend of TGF-β and vimentin between smokers and nonsmokers. There was no change in the protein level of plasminogen activator inhibitor-1 (PAI-1) between smokers and nonsmokers. Overall, our results confirm the upregulation in mesenchymal transition markers in the lungs of smokers compared with nonsmokers. In our previous study, we have shown decreased protein levels of REV-ERBα in smokers compared with nonsmokers (14). Here, we proposed to determine how REV-ERBα affects EMT activation.

### REV-ERBα deficiency promotes clock dysregulation in the lungs following subchronic CS exposure.

**Figure 2:**
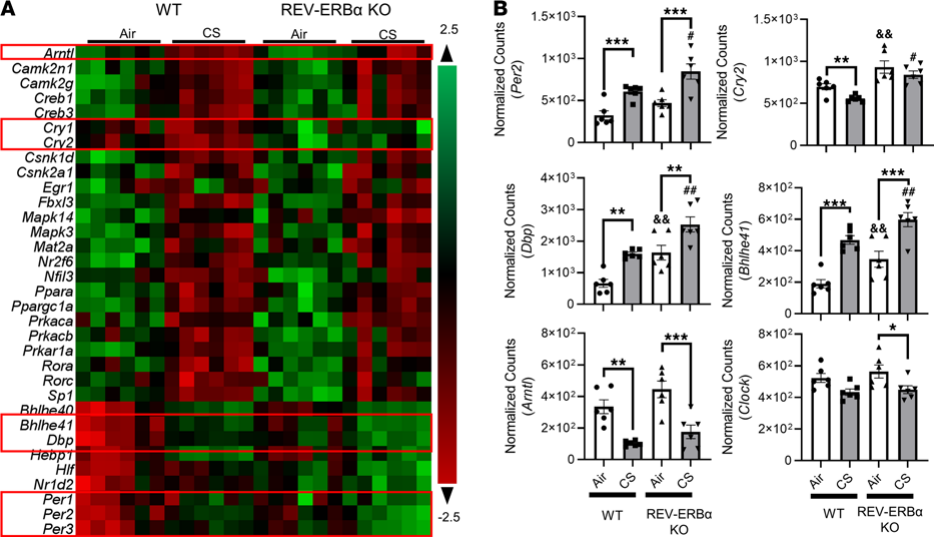
Subchronic cigarette smoke exposure affects circadian focused mRNA expression analyzed by NanoString. Total RNA was isolated from the lungs of mice exposed to air and cigarette smoke (CS) for 30 days. Our customized NanoString panel (circadian gene focused) was used to screen the potential targets via nCounter SPRINT Profiler. Normalization of absolute RNA count and data analysis were done by nSolver software. The overview of all the dysregulated targets is shown as a heatmap (**A**), and selected gene transcription changes are shown separately (**B**). Data are shown as mean ± SEM (*n* = 6; *P < 0.05, **P < 0.01, ***P < 0.001 between groups; ^#^*P* < 0.05, ^##^*P* < 0.01 compared with CS-exposed WT group; ^&&^*P* < 0.01 compared with WT air controls, 1-way ANOVA with Šidák correction).

To determine how subchronic CS exposure affects circadian clock gene targets in mouse lungs, we have employed a customized NanoString nCounter panel for gene transcription analysis. Subchronic CS exposure causes circadian clock dysregulation in the lungs (Figure 2 and Supplemental Figure 1; supplemental material available online with this article; https://doi.org/10.1172/jci.insight.145200DS1). The core clock-controlled genes (CCGs), such as *Arntl*, *Per2*, *Per3*, *Cry2*, and *Rora*, showed altered expression following subchronic CS exposure. Other circadian-related genes, such as D site of albumin promoter binding protein (*Dbp*), basic helix-loop-helix family, member e41 (*Bhlhe41*), basic helix-loop-helix family, member e40 (*Bhlhe40*), casein kinase 1 δ (*Csnk1d*), casein kinase 2 α 1 (*Csnk2a1*), F-box and leucine-rich repeat protein 3 (*Fbxl3*), mitogen-activated protein kinase 14 (*Mapk14*), methionine adenosyl transferase 2A (*Mat2a*), nuclear receptor subfamily 2 group F member 6 (*Nr2f6*), and nuclear factor, IL-3 regulated (*Nfil3*), were all dysregulated after subchronic CS exposure. In *REV-ERBα*–KO mice, subchronic CS exposure further upregulated circadian gene transcription of selected genes, such as *Per2*, *Per3*, *Dbp*, *Bhlhe41*, and hepatic leukemia factor (*Hlf*) (Figure 2 and Supplemental Figure 1). Notably, *Dbp*, *Bhlhe41*, and *Per2* genes showed a significant increase in *REV-ERBα–*KO mice exposed to CS compared with their respective air group control. However, we only observed a remarkable dysregulation in CCGs at the gene level. There was an increase in the protein abundance of BMAL1 after subchronic CS exposure (Supplemental Figure 2).

### REV-ERBα deficiency accelerates EMT activation following subchronic CS exposure.

**Figure 3:**
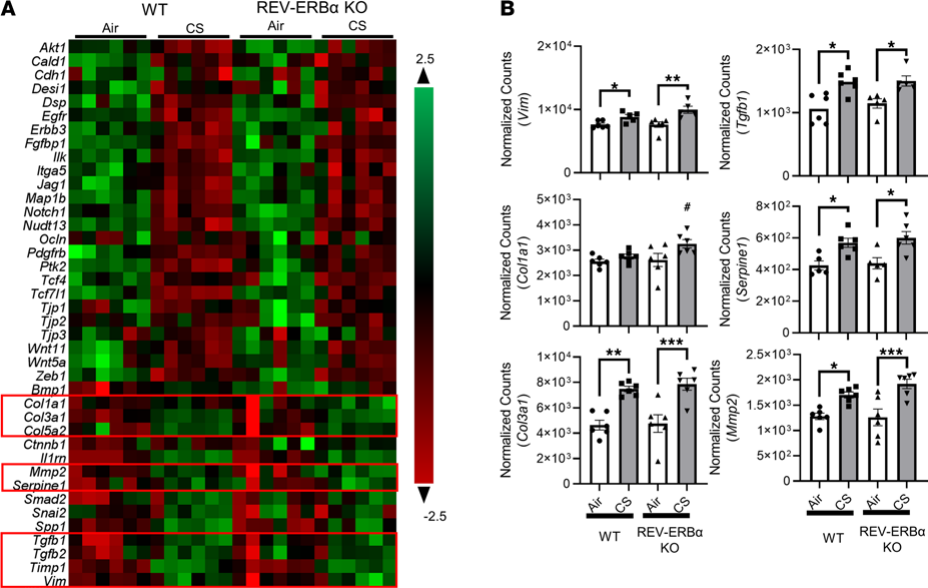
Subchronic CS exposure affects EMT-focused mRNA expression analyzed by NanoString. Total RNA was isolated from the lungs of mice exposed to air and CS for 30 days. Our customized NanoString panel (EMT gene focused) was used to screen the potential targets via nCounter SPRINT Profiler. Normalization of absolute RNA count and data analysis were performed by nSolver software. The overview of all the dysregulated targets was shown as a heatmap (**A**), and selected gene transcription changes were shown separately (**B**). Data are shown as mean ± SEM (*n* = 6; *P < 0.05, **P < 0.01, ***P < 0.001 between groups; ^#^*P* < 0.05 compared with CS- exposed WT group; 1-way ANOVA with Šidák correction).

**Figure 4:**
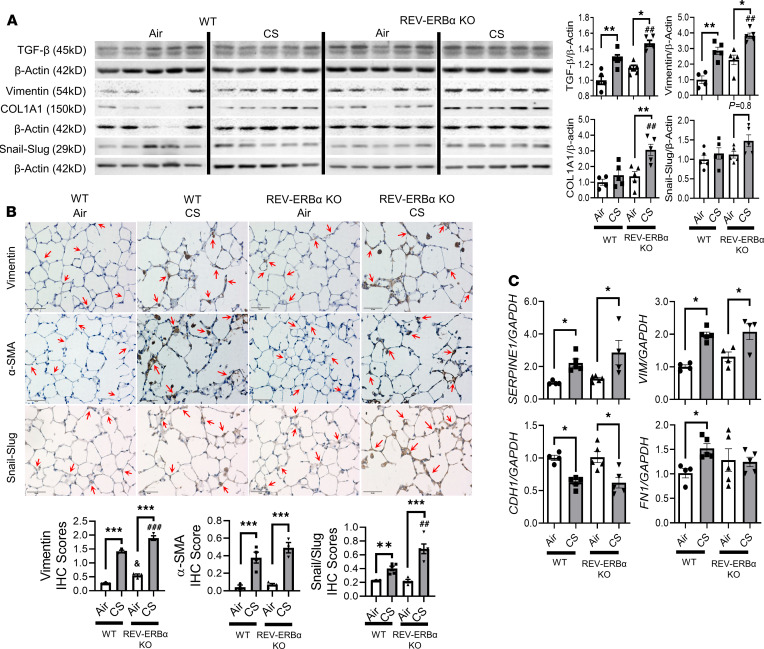
Subchronic CS exposure–induced mesenchymal transition in REV-ERBα–dependent manner. Protein abundance of mesenchymal markers in mouse lung affected by subchronic CS exposure (1 month/30 days) were analyzed by Western blotting. (**A**) Representative blot images (TGF-β, vimentin, COL1A1, and snail-slug) are shown, and densitometry analyses are done individually. Different groups were run on the same membrane but were noncontiguous. COL1A1 and vimentin were probed in the same membrane, and β-actin was used as an endogenous control (*n* = 5/group). (**B**) The localizations of dysregulated vimentin, α-smooth muscle actin (α-SMA), and snail-slug were observed via immunohistochemical staining. Regions of interest were pointed by red-arrow. Relative IHC score based on positive staining intensity was performed in a blind manner (*n* = 3–4/group); specific protein accumulation in alveoli was denoted by red arrows (original magnification, ×40; scale bar: 50 μm) (**C**). RNA isolated from lung tissues were used to determine the gene expression (*Serpine1*, *Vim,* *Cdh1*, and *Fn1*) by qRT-PCR. GAPDH was used as an endogenous control, and gene fold change was calculated by 2**^-ΔΔCt^** method (*n* = 4–5/group). Data are shown as mean ± SEM (*P < 0.05, **P < 0.01, ***P < 0.001 between groups; ^#^*P* < 0.05 compared with CS-exposed WT group; ^&^*P* < 0.05 compared with WT air controls; 1-way ANOVA with Šidák correction).

To further investigate the correlation between REV-ERBα and CS-induced EMT, we examined EMT-associated gene expression and protein abundances/localizations in the lungs of subchronic CS-exposed WT and *REV-ERBα*–KO mice (Figures 3 and 4). At the gene transcription level, only collagen type I α I (*Col1a1*) showed significant upregulation in CS-exposed REV-ERBα–KO mice compared with CS-exposed WT mice (Figure 3). Most of the EMT markers, especially the mesenchymal markers, including vimentin (*Vim*), *Tgfβ1*, serpin family E member 1 (*Serpine1*), collagen type III α I (*Col3a1*), matrix metalloproteinase-2 (*Mmp2*), and TIMP metallopeptidase inhibitor 1 (*Timp1*), were increased in CS-exposed mice compared with air group controls (Figure 3, Figure 4C, and Supplemental Figure 3). Surprisingly, we showed a decreased gene expression of Snail (also known as *Snai1*) after CS exposure in both WT and KO mice (Supplemental Figure 3). In addition, the epithelial marker *Cdh1* was decreased in CS-exposed (WT or KO) mice (Figure 4C).

**Figure 5:**
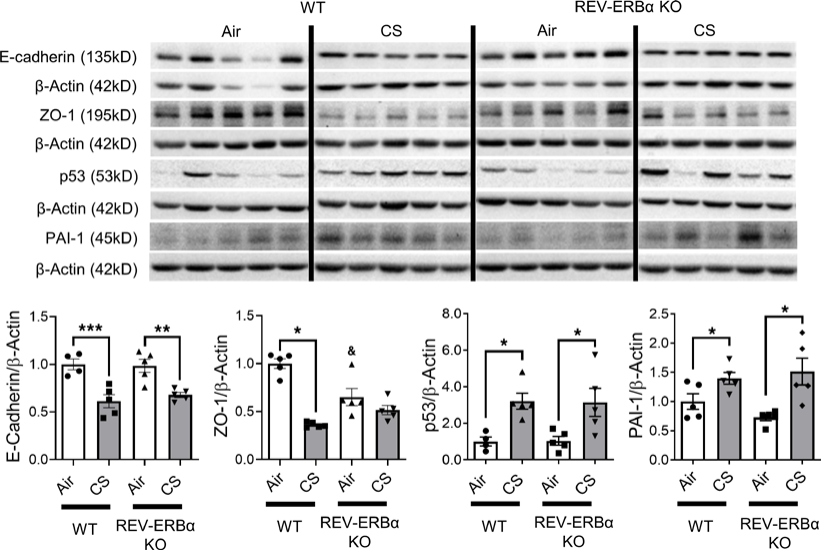
Protein abundance of EMT markers were affected by subchronic CS exposure. Lungs from mice exposed to CS for 30 days were homogenized, and protein abundances were measured by Western blotting. Representative blot images are shown and relative protein fold change of E-cadherin, ZO-1, p53, and PAI-1 are analyzed based on densitometry with β-actin as the endogenous control. Different groups were run on the same membrane, but were noncontiguous. Data are shown as mean ± SEM (*n* = 5, *P < 0.05 between groups; **P < 0.01 between groups, ***P < 0.001 between groups; ^&^*P* < 0.05 compared with WT air controls; 1-way ANOVA with Šidák correction).

EMT-related protein abundances and localizations were measured to further determine the role of REV-ERBα in CS-induced EMT. All mesenchymal markers, including vimentin, COL1A1, TGF-β, PAI-1, and p53, were increased in CS-exposed mice (Figures 4 and 5). Additionally, vimentin, COL1A1, TGF-β, and snail-slug protein expressions were augmented in CS-exposed REV-ERBα–KO mice (Figure 4A). To further understand the altered expression and localization of mesenchymal markers, vimentin, αSMA, and snail-slug were stained immunohistochemically. In agreement with immunoblotting results, CS exposure significantly increased vimentin expression in the alveolar region (Figure 4B). In *REV-ERBα–*KO mice, further increase in the protein abundance of vimentin and snail-slug in the alveolar regions of the lungs was semiquantitatively analyzed (Figure 4B and Supplemental Figure 4). αSMA expression was also increased in alveolar epithelium of CS-exposed mice independent of REV-ERBα (Figure 4B and Supplemental Figure 4). Most of the EMT markers, such as αSMA, vimentin, and snail-slug, were expressed near the airway, whereas CS exposure augmented mesenchymal expression in alveoli (Supplemental Figure 4). PAI-1 and p53 protein levels were increased after subchronic CS exposure in both WT and *REV-ERBα*–KO mice (Figure 5). The epithelial markers E-cadherin and ZO-1 were decreased in CS-exposed WT and *REV-ERBα–*KO mice (Figure 5).

MMPs are the essential components in ECM remodeling and deposition, and MMP2, MMP9, and MMP12 are upregulated after CS exposure. We evaluated the protein abundance of MMP2, MMP9, and MMP12 in mouse lung after 30 days of exposure (Supplemental Figure 5). We only found increased protein levels of MMP12 in CS-exposed REV-ERBα–KO mice, whereas MMP2 and MMP9 showed no alteration in protein abundance (Supplemental Figure 5). However, we observed an increase in MMP2 gene expression in both CS-exposed WT and *REV-ERBα*–KO mice (Figure 3). There was no difference in MMP9 gene expression among all the treatment groups (Supplemental Figure 3).

### REV-ERBα–deficient mice exposed to subchronic CS show altered ECM and lung remodeling.

**Figure 6:**
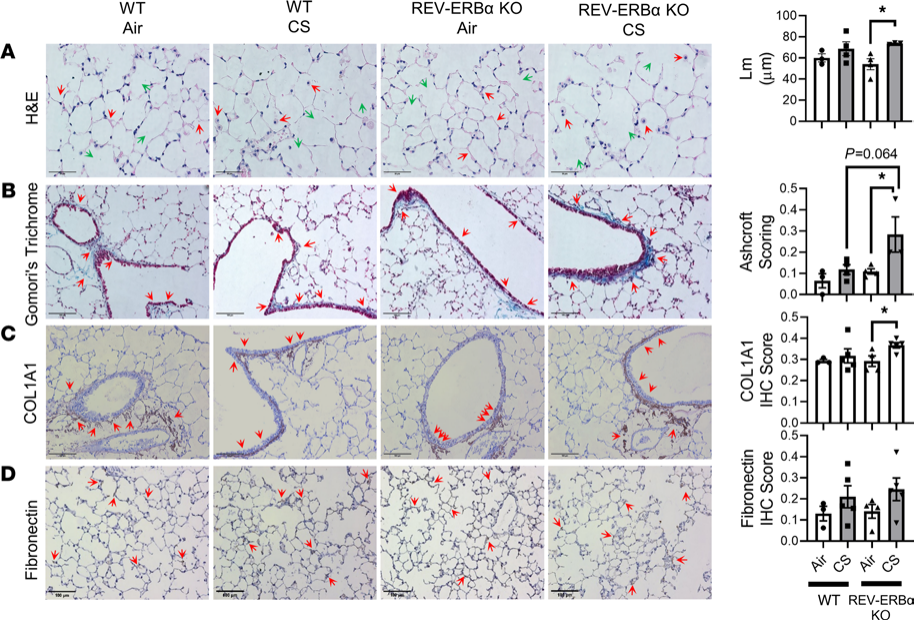
Airspace enlargement and abnormal ECM deposition were induced by subchronic CS exposure in REV-ERBα–KO mice. Subchronic 30 days of CS exposure induced airspace enlargement observed by (**A**). H&E (original magnification, ×40; scale bar: 50 μm) (green arrows indicated airspace enlargement and red arrows indicated inflammatory responses) and abnormal ECM deposition observed via **B**. Gomori’s Trichrome (original magnification, ×20) staining (scale bar: 100 μm) (**C**). IHC staining COL1A1 (original magnification, ×20; scale bar: 100 μm) (**D**). Fibronectin (original magnification, ×20; scale bar: 100 μm). Relative IHC score based on positive staining intensity was performed in a blind manner and denoted by red arrows. Data are shown as mean ± SEM (*n* = 3–4/group, *P < 0.05; 1-way ANOVA with Šidák correction).

We showed that CS-induced mesenchymal transition is associated with circadian clock gene REV-ERBα in the lungs. We investigated this association by using histological analysis of lung sections for alveolar destruction/airspace enlargement and ECM remodeling (Figure 6 and Supplemental Figure 6). We measured lung mean linear intercept (Lm) analysis using H&E-stained images and Gomori’s Trichrome staining to quantify collagen deposition in the lungs from different experimental groups. Type I collagen and fibronectin were also immunohistochemically stained to determine the ECM remodeling. We found that the Lm of airspace was increased but not significantly in WT mice exposed to CS compared with air exposed controls. However, *REV-ERBα–*KO mice exposed to subchronic CS exhibited significantly increased Lm compared with their respective air group controls (Figure 6A and Supplemental Figure 6). As expected, various immune cells were found in the airspace of both CS-exposed WT and REV-ERBα–KO mice compared with respective air-exposed controls (Figure 6A and Supplemental Figure 6). Additionally, a significant increase in collagen deposition around the airway was found in CS-exposed *REV-ERBα–*KO mice, whereas CS-exposed WT mice showed similar collagen deposition compared with the air-exposed control (Figure 6B). We also found a higher type-1 collagen deposition in CS-exposed *REV-ERBα–*KO mice compared with air controls (Figure 6C), complementing the Gomori’s Trichrome staining results. However, we did not observe any significant difference in fibronectin localization and expression (Figure 6D).

**Figure 7:**
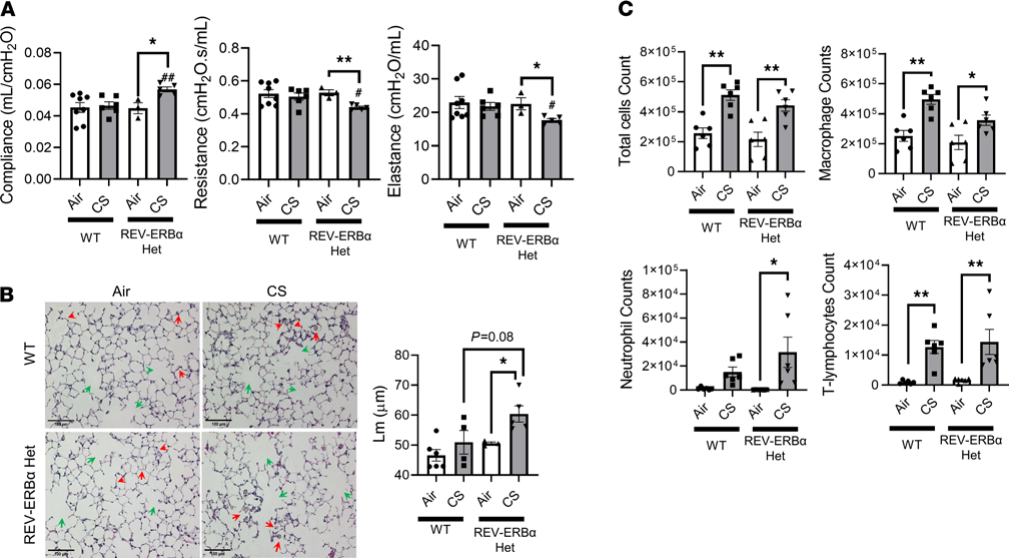
Altered airspace enlargement, lung mechanics, and inflammation were induced by chronic CS exposure in REV-ERBα Het mice. Lung mechanics, inflammatory cell influx in bronchoalveolar lavage fluid (BALF), and airspace enlargement induced by chronic CS exposure (4 months) were determined by Flexivent, flow cytometry, and lung morphometry. (**A**) Lung mechanics (lung compliance resistance and elastance) were measured after chronic CS exposure. (**B**) Lung histological analysis were conducted using H&E-stained sections, and mean linear intercept (Lm) analysis was performed using Metamorph software (original magnification, ×20; scale bar: 100 μm) from H&E-stained images (green arrows indicated airspace enlargement and red arrows indicated inflammatory responses). (**C**) Total cell was counted by Bio-Rad cell counter using Trypan blue staining. Differential inflammatory cell counts (macrophage, neutrophil, and T lymphocytes) were determined (cells/mL) by flow cytometry. Data are shown as mean ± SEM (*n* = 5–10; *P < 0.05, **P < 0.01, between groups; ^#^*P* < 0.05, ^##^*P* < 0.01 compared with CS-exposed WT group; 1-way ANOVA with Šidák correction).

We exposed REV-ERBα Het and WT C57 mice to CS for 4 months and found similar alterations of lung morphometry and inflammation (Figure 7). We found increased lung compliance and decreased lung resistance and elastance in CS-exposed *REV-ERBα* Het mice compared with WT mice or those in the air group (Figure 7A). In concurrence with the increased compliance, we observed airspace enlargement in CS-exposed *REV-ERBα* Het mice compared with that in WT mice (Figure 7B). We observed that the baseline Lm results varied between 1- and 4-month mouse groups, owing to mouse batch differences, such as their age and quantitation involved in scoring. Further, we found that chronic CS exposure increased inflammatory cell influx in bronchoalveolar lavage fluid (BALF) with increased macrophage, neutrophils, T lymphocytes, and total cells (Figure 7C). However, *REV-ERBα* Het mice did not show significant difference in altered inflammatory cell counts compared with WT mice (Figure 7C). To support our finding for lung morphometry and inflammatory responses, we found a similar change histologically (Figure 7B and Supplemental Figure 7). Increased airspace enlargement was only observed in *REV-ERBα* Het CS-exposed mice (original magnification, ×10 and ×20, denoted by green arrows); lung injury and infiltration of inflammatory cells in airspace were observed in both WT and *REV-ERBα* Het CS-exposed mice (original magnification, ×10 and ×20, denoted by red arrows) (Supplemental Figure 7).

### REV-ERBα agonist treatment attenuates lung inflammatory response caused by acute CS exposure.

**Figure 8:**
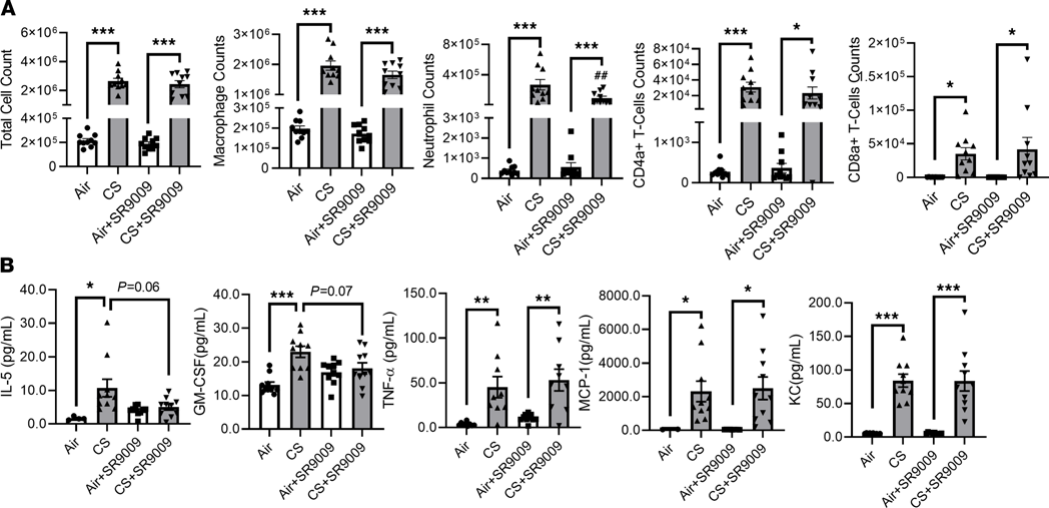
Acute CS exposure–induced inflammatory response was reduced by REV-ERBα agonist. Proinflammatory cell influx and cytokines in BALF induced by acute CS exposure (10 days) were determined by flow cytometry and Luminex. (**A**) Total number of inflammatory cells was counted by Bio-Rad cell counter using Trypan blue staining. Differential inflammatory cell counts (macrophage, neutrophil, CD4/CD8 T lymphocytes) were determined (cells/mL) by flow cytometry. (**B**) Relative cytokines were determined by Luminex. A total of 2 technical repeats were done to calculate the final concentration. Data are shown as mean ± SEM (*n* = 10; *P < 0.05, **P < 0.01, ***P < 0.001, between groups; ^##^*P* < 0.01 compared with CS exposed WT group; 1-way ANOVA with Šidák correction).

We have shown that REV-ERBα deficiency promotes abnormal mesenchymal transition, and our previous study has shown that REV-ERBα is associated with increased lung inflammation induced by subchronic CS exposure (13). Here, we demonstrate the therapeutic potential of REV-ERBα agonist (SR9009) in acute CS-induced inflammation. After 10 days of CS exposure, total cells from BAL fluid increased in both vehicle and Rev-erb agonist (SR9009) treatment groups (Figure 8A). The percentages of inflammatory cells were determined by flow cytometry, and the percentages of macrophages, CD4 T cells, and CD8 T cells were similar in acute CS-exposed mice treated with vehicle or SR9009 (Figure 8A). Intriguingly, acute CS-induced increase in neutrophils was inhibited by REV-ERBα agonist (SR9009) treatment (Figure 8A).

We measured the BAL fluid cytokines by Luminex to identify the inflammatory responses induced by acute CS exposure with or without SR9009 treatment. We found that only CS-induced increases in IL-5 (*P* = 0.06) and GM-CSF (*P* = 0.07) were inhibited by SR9009 treatment without significant differences. Other BAL fluid cytokines, such as MCP-1 and KC, had significantly increased in the CS alone and CS+SR9009 treatment groups (Figure 8B and Table 1).

### REV-ERBα agonist treatment reduced alterations in EMT markers caused by acute CS exposure.

**Figure 9:**
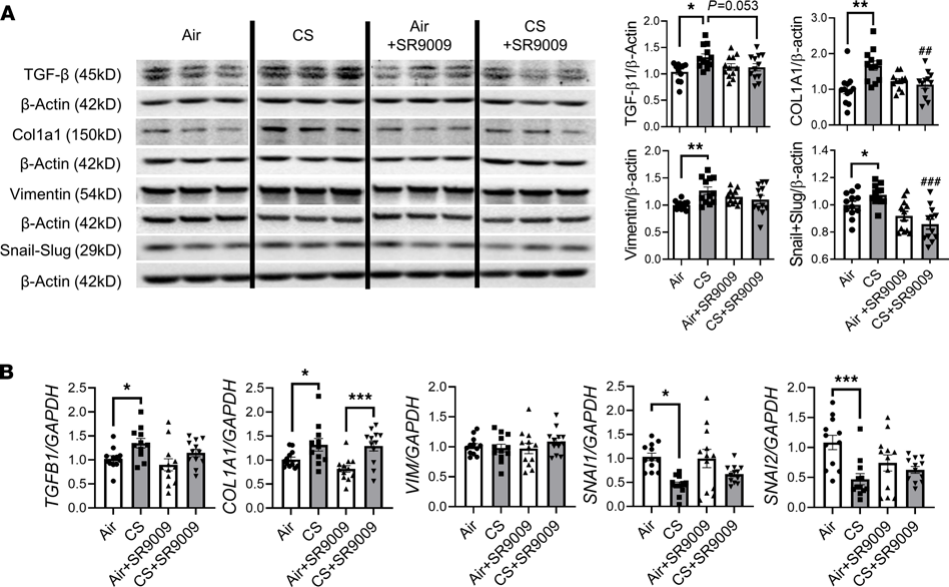
Acute CS exposure–induced mesenchymal transitions were inhibited by REV-ERBα agonist. Lungs from mice exposed to acute CS exposure with or without REV-ERBα agonist (SR9009) administration were homogenized and protein abundance were analyzed by Western blotting. (**A**) Representative blot (TGF-β, vimentin, COL1A1, and snail-slug) images are shown, and densitometry analyses are done individually. Different groups were run on the same membrane but were noncontiguous, and β-actin was used as an endogenous control. (**B**) RNA isolated from lung tissues was used to determine the gene expression (*Tgfb1*, *Col1a1*, *Vim*, *Snai1*, and *Snai2*) by qRT-PCR. GAPDH was used as an endogenous control, and gene fold change was calculated by 2^-ΔΔCt^ method (*n* = 11–12/group). Data are shown as mean ± SEM (*P < 0.05, **P < 0.01, ***P < 0.001, between groups; ^##^*P* < 0.01, ^###^*P* < 0.001 compared with CS-exposed group; 1-way ANOVA with Šidák correction).

**Figure 10:**
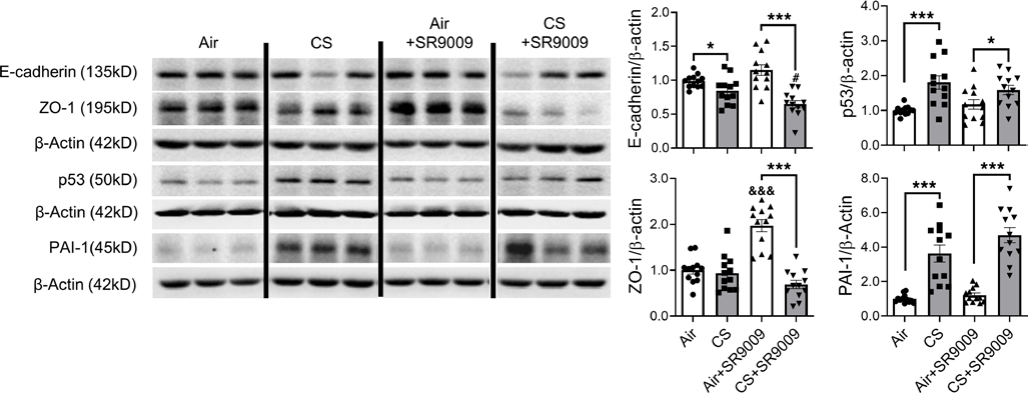
Protein abundance of EMT markers were affected by acute CS exposure. Lungs from mice exposed to CS for 10 days with SR9009 administration were homogenized, and protein abundance were measured by Western blotting. Representative blot images are shown, and relative protein fold changes of E-cadherin, ZO-1, p53, and PAI-1 are analyzed based on densitometry with β-actin as the endogenous control. E-cadherin and ZO-1 were probed in the same membrane. Different groups were run on the same membrane, but were noncontiguous. Data are shown as mean ± SEM (*n* = 11–12. *P < 0.05, ***P < 0.001 between groups; ^&&&^*P* < 0.001 compared with WT air controls; 1-way ANOVA with Šidák correction).

We have shown that subchronic CS exposure resulted in mesenchymal transition in a REV-ERBα–dependent manner. Here, we demonstrate the protective role of SR9009 against dysregulated EMT following acute CS exposure (Figures 9 and 10). As expected, vimentin, COL1A1, TGF-β, and snail-slug were upregulated by acute CS exposure, and SR9009 treatment significantly reduced COL1A1 and snail-slug protein abundance. Additionally, SR9009 treatment also inhibited the CS-induced increase in vimentin and TGF-β, which was not significantly different between the air- and CS-exposed SR9009 treatment groups (Figure 9A). We also measured the gene expression levels of *Tgfb1*, *Col1a1*, *Vim*, *Snai1*, and *Snai2* (Figure 9B). We observed increased *Tgfb1* and *Col1a1* after CS exposure, and SR9009 administration was able to inhibit the upregulation of *Tgfb1* but not *Col1a1* (Figure 9B). There was no difference in *Vim* expression among groups (Figure 9B). Interestingly, we found decreased gene expression of *Snai1* and *Snai2* after 10 days of CS exposure (Figure 9B). Other mesenchymal markers (PAI-1 and p53) were increased after acute CS exposure in both CS alone and CS+SR9009 treatment groups (Figure 10). We also measured the protein abundance of epithelial markers and found that acute CS exposure decreased E-cadherin protein levels, whereas CS+SR9009 treatment further decreased E-cadherin levels. Interestingly, there was no change in the protein levels of ZO-1 between air- and CS-exposed mice, whereas SR9009 treatment augments ZO-1 in air+SR9009 treatment group compared with both air alone and CS+SR9009 treatment groups (Figure 10).

We showed increased protein abundance of MMP12 in mouse lungs after 30 days of CS exposure and increased gene transcript level of MMP2. We measured the protein expression levels of MMP2, MMP9, and MMP12 and gene levels of *Mmp2, Serpine1*, and *Fn1* in 10-day-exposed mouse lungs as well (Supplemental Figure 8). Surprisingly, we observed a decreased protein expression level of MMP12 after 10 days of CS exposure (Supplemental Figure 8), whereas increased MMP12 was found after 30 days of CS exposure (Supplemental Figure 5). Similarly, gene expression of MMP2 was upregulated after CS exposure, and SR9009 administration failed to attenuate MMP2 gene expression. There was no difference in the protein expression of MMP9 and MMP2 as well as the gene transcripts of *Fn1* and *Serpine1* (Supplemental Figure 8).

Finally, we measured the protein abundance of a few key circadian clock molecules to understand the possible protective role of SR9009 treatment in acute CS-exposed mice. Immunoblot analysis of circadian clock targets confirmed an increase in the protein abundance of REV-ERBα in both acute air- and CS-exposed mice treated with SR9009 (Supplemental Figure 9). We found increased protein abundance of RORα in CS-exposed mice treated with SR9009 compared with vehicle/CS-exposed controls. BMAL1 protein levels remain unaffected among different exposure and treatment groups, and CLOCK protein expression was decreased in CS+SR9009 treatment group compared with the air+SR9009 group (Supplemental Figure 9). Overall, results revealed REV-ERBα agonist treatment mediates attenuation of lung inflammation and EMT phenotype via augmenting REV-ERBα expression in the lungs.

### REV-ERBα agonist treatment prevents fibroblast differentiation in vitro in HFL-1 cells.

**Figure 11:**
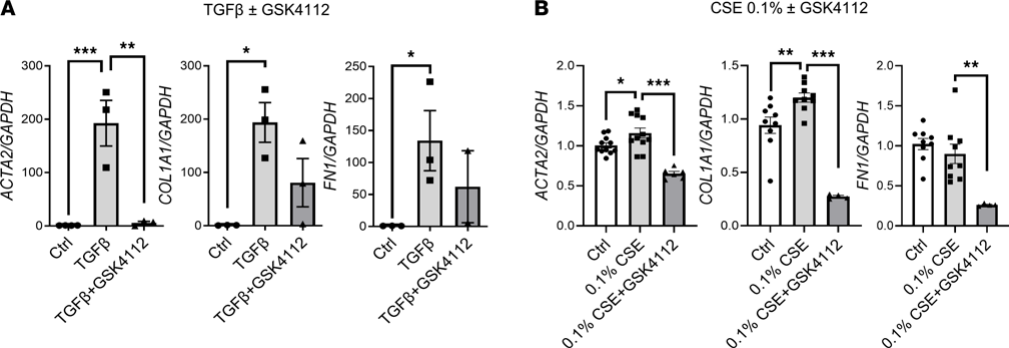
TGF-β/CS–activated fibroblast differentiation was inhibited by REV-ERBα agonist. Human lung fibroblast (HFL-1) cells were treated with (**A**) TGF-β with or without REV-ERBα agonist (GSK4112) for 3 days or (**B**) 0.1% CSE with or without GSK4112 for 2 days. RNA isolated from cells were used to determine the gene expressions (*ACTA2, COL1A1,* and *FN1*) by qRT-PCR. GAPDH was used as an endogenous control for normalization. Data are shown as mean ± SEM (*n* = 3 for **A**, and *n* = 4–9 for **B**; *P < 0.05, **P < 0.01, ***P < 0.001, between groups; 1-way ANOVA with Šidák correction for **A**, and unpaired Student’s *t* test for **B**).

To determine if REV-ERBα plays an essential role in CS-induced EMT, we investigated the role of REV-ERBα in fibroblast differentiation. Human fetal lung fibroblast 1 (HFL-1) cells were treated with TGF-β with or without GSK4112 (REV-ERBα agonist) for 3 days (Figure 11A). The TGF-β–induced increase in gene expression of *ACTA2* (αSMA) was significantly inhibited by GSK4112 treatment in HFL-1 cells, whereas mRNA levels of *COL1A1* and *FN1* (fibronectin) were reduced by GSK4112 but not significantly. We also treated HFL-1 cells with CS extract (CSE) with or without GSK4112 and measured the same fibroblast differentiation markers (Figure 11B). We found a high concentration of CSE (0.25%) showed reduced expression of *ACTA2* and *FN1*, whereas low 0.1% CSE increased gene expression of *ACTA2* and *COL1A1,* without change in the expression of *FN1* (Supplemental Figure 10). Similarly, we showed a significant reduction in *ACTA2*, *COL1A1*, and *FN1* gene expression after treatment with 0.1% CSE+GSK4112 (Figure 11B). These findings indirectly support the role REV-ERBα during inflammation induced mesenchymal differentiation in the lungs (Figure 11).

## Discussion

CS exposure induces lung inflammation, followed by the development of chronic lung diseases, such as emphysema/COPD and lung remodeling. Previously, we have shown that the CS-induced inflammatory response in mice, which was associated with circadian molecular clock dysfunction (13, 16) REV-ERBα expression, was reduced in smokers and patients with COPD compared with healthy controls as well as in LPS-treated peripheral blood mononuclear cells (14). In this study, we found that CS-induced EMT was associated with *REV-ERBα* in both acute and subchronic mouse models. Based on our findings, the role of *REV-ERBα* in mesenchymal activation following CS exposure in mice was warranted. As expected, REV-ERBα agonists (SR9009 and GSK4112) both showed potential therapeutic effects in fibroblast phenotype dysregulation, and SR9009 treatment showed attenuation against CS-induced acute lung inflammatory responses. NanoString data analysis confirmed that the circadian clock gene targets were affected by subchronic CS exposure, which may be responsible for the abnormal lung development (EMT) and reduced repair at the early stage of CS-induced lung injury.

Circadian rhythms exist in almost all the organs, such as lungs, liver, heart, and spleen. Previous studies have reported that several physiological processes, such as body temperature, hormone release, and sleep-wake cycle, are regulated by circadian rhythms. Additional evidence suggests that loss of specific circadian clock molecules, such as BMAL1, REV-ERBα, and CRY1, could promote an inflammatory response and metabolic dysfunction in target organs (20–22). Our results are in agreement with those of a previous study describing the association between lung inflammation and decreased levels of BMAL1 (23). Previous studies demonstrated the antiinflammatory effects of certain clock molecules, including REV-ERBα, CRY1, CRY2, RORα, and BMAL1 (24–27). In this study, we showed that the expression of several key circadian clock targets were significantly downregulated following subchronic CS exposure. From our previous study, we also observed decreased CRY2, BMAL1, and REV-ERBα in smokers and patients with COPD compared with healthy controls (14). The heterodimer CLOCK/BMAL1 activates the transcription of CRYs, PERs, and RORα; downregulation of BMAL1, CRYs, and RORα showed the tendency of circadian feedback loop inhibition (28). However, we also observed an increase in the expression of PERs (*Per1/Per2/Per3*) and *Dbp*, following subchronic CS exposure. It should be noted that the expression of genes within the mammalian PER families (*Per1/Per2/Per3*) was not only regulated by CLOCK/BMAL1 activated E-boxes, but also regulated by DBP via binding to the D-box (28). Overall, our results suggest that acute and subchronic CS exposure induced alterations in circadian clock gene expression which may have an impact in disrupting the positive-negative feedback loops, thus culminating in the circadian clock dysfunction in the lungs

Our data on transcriptomic analysis by NanoString revealed subchronic CS exposure–induced circadian clock dysfunction in the lungs. *REV-ERBα*–deficient mice showed alterations in the expression of circadian clock–controlled genes, such as *Per1*, *Per2*, *Per3*, *Dbp*, and *Bhelhe41*, whereas *Arntl*, *Cry1*, and *Cry2* expression was not affected. As previously reported, *REV-ERBα*–KO mice showed increased transcription of *Dbp* (24), and DBP is capable of activating the expression of PERs (28). In this study, we found that *REV-ERBα* deletion resulted in CS-induced expression of *Dbp* and *Per2*. Interestingly, *Per2* also served as a tumor suppressor, targeting cell proliferation and cell-cycle progression (18). The role of *Per2* and *Dbp* need further investigation, especially their interaction with REV-ERBα. Based on our gene transcription data, selective clock antagonists/agonists specific for CRYs, RORα, and PERs and activators of protein kinases showed possible protective effects against CS-induced lung injury. However, their therapeutic role against CS-induced circadian clock dysfunction requires further studies.

We have shown that EMT activation was observed in smokers relative to nonsmokers and that subchronic CS exposure in mice had induced EMT in a REV-ERBα–dependent manner. It is obvious that EMT induced by TGF-β downregulates epithelial markers (E-cadherin and ZO-1) along with activation of mesenchymal markers (vimentin, collagen, fibronectin, and αSMA) (29). CLOCK/BMAL1 heterodimer, the major transcription activator of REV-ERBα, has been shown to be associated with EMT markers E-cadherin and N-cadherin (30). Similarly, silencing BMAL1 attenuated the TGF-β–induced EMT in mouse lungs as well as in lung epithelium (31). Moreover, TGF-β induction in mouse lungs resulted in increased BMAL1 along with decreased REV-ERBα and RORα levels (31). The indirect connection between EMT markers and circadian molecules cannot be ruled out. In this study, subchronic CS exposure reduced transcriptional levels of BMAL1 and CLOCK, while increasing BMAL1 at the protein levels. The dysregulated BMAL1 and CLOCK might be one of the reasons for EMT induced by CS, and the detailed mechanisms need further investigation. Interestingly, REV-ERBα has been associated with the pathogenesis of pulmonary fibrosis with possible interaction with the transcription factor TBPL1 (17). We have shown that dysregulated EMT markers, which are associated with *REV-ERBα* KO, are mostly mesenchymal markers (snail-slug, vimentin, COL1A1, and TGF-β), whereas epithelial markers showed reduced expression between the *REV-ERBα*–KO and WT groups. Our results, in part, highlight the important role of REV-ERBα in fibroblasts, with these cells having a crucial role in exacerbating fibrotic responses in the lungs (17). The robust circadian gene oscillations in fibroblasts present the importance of circadian rhythmicity in fibroblasts and also have been shown to regulate the wound healing response in vitro and in vivo (32, 33). In addition to the importance of fibroblast in stress responses, diminished REV-ERBα levels in lung epithelium could also enhance the inflammation induced by LPS (21). We have also observed that vimentin snail-slug and collagen accumulation were increased by CS exposure in the alveolar and bronchial epithelium and *REV-ERBα*–KO mice showed further augmentation of mesenchymal markers. Our results emphasize the importance of REV-ERBα regulating EMT markers in lung epithelium during CS-induced lung inflammation. We also measured the protein abundance and gene expression of MMPs (MMP2, MMP9, and MMP12) after acute or subchronic CS exposure; however, we observed little or no changes in MMPs associated with REV-ERBα. Currently, to our knowledge there is no report on the dysregulated EMT associated with REV-ERBα, specifically in lung epithelium. Our data demonstrate the accumulation of mesenchymal markers in lung epithelium after subchronic CS exposure. Understanding the role of the specific circadian clock targets using genetic (KO and overexpression) and pharmacologic (selective agonist and antagonist) models associated with altered EMT will shed light into the cellular processes that play crucial role at early stage of chronic lung disease.

Previously, we have shown that subchronic CS exposure induced lung inflammation in a REV-ERBα–dependent manner (14), which is in agreement with our data presented in another study (13). *REV-ERBα–*KO mice exposed to subchronic CS (30 days) increased neutrophil percentage (13), and our data demonstrate that REV-ERBα agonist (SR9009) treatment reduced the neutrophil percentages after 10 days of acute CS exposure. Previously, we showed both LPS and CSE induced inflammatory response in human small airway epithelial cells and mouse lung fibroblasts were attenuated by REV-ERBα agonist (GSK4112) (13). Here, we showed that GSK4112 treatment could ease TGF-β–induced fibroblast differentiation by inhibiting the activation of mesenchymal markers in HFL-1 cells.

Interestingly, some of the EMT genes showed rhythmic oscillation during a 24-hour period, including *Snai2*, *Col1a1*, *Col3a1*, and *Col5a2,* as confirmed by the CIRCA database (http://circadb.hogeneschlab.org). A recent study has explained that collagen secretion and homeostasis are regulated by the circadian clock (34). Others, such as *Serpine1* (PAI-1), *Tgfb1*, and *Tjp3*, also showed rhythmic expression at the transcript level. Since PAI-1 expression peaks at 6 am, and it has been shown that CLOCK/BMAL1 heterodimer induced activation of the *Serpine1* promoter through E-box (35), this may lead to an increased risk of cardiovascular disorders during early morning hours (36). Similarly, rhythmic expression of *Tgfb1* was also regulated by the CLOCK/BMAL1 heterodimer through E-box and CLOCK deficiency attenuated the oscillation (37). As TGF-β1 is a common activator of EMT, and TGF-β pathway activation could also induce the abnormal ECM deposition, such as collagen and αSMA. Targeting the novel gene transcriptional rhythms, which regulate canonical signaling pathways related to EMT, may be critical to devise potential therapeutic strategies against chronic lung disease in which ECM remodeling plays an important role.

In this study, we investigated the therapeutic potential of REV-ERBα agonist in abnormal EMT activated by CS exposure as well as the antiinflammatory effects. We and others have reported that REV-ERBα agonists were capable of attenuating inflammatory responses (13, 21, 24). Recently, REV-ERBα agonists have been shown to modulate fibrotic responses in mice and ex vivo human lung tissue (17). Additionally, SR9009 treatment in tendon fibroblasts showed a decrease in collagen fiber formation per cell, and the CRY1/2 agonist KL001 enhanced the transcript levels of genes (*Sec61a2*, *Mia3*, *Pde4d*, and *Vps33b*) that regulate collagen trafficking, fiber assembly, and the amount of collagen fiber formation per cell (34). Furthermore, SR9009 exhibited antitumor effects on small-cell lung cancer through REV-ERBα–mediated autophagy (38). Based on our data, the dysregulation in EMT and TGF-β–induced fibroblast differentiation was modulated by REV-ERBα agonists, SR9009 and GSK4112.

Apart from SR9009 and GSK4112, there are other REV-ERBα ligands, such as SR9011, GSK2945, SR12418, GSK0999, GSK5072, and GSK2667, that are available and currently being tested (39). SR9011 and SR9009 are designed based on the chemical structure of GSK4112 for in vivo evaluation, because GSK4112 has poor solubility in PBS as well as poor pharmaceutical properties (39, 40). We have proven that SR9009 is able to modulate CS-induced inflammation and EMT. Similarly, GSK2945 was designed based on the similarities observed in the structure of GSK4112, whereas GSK2945 showed both REV-ERBα activation and/or inhibition that varies owing to the cell-type/organ specificity (39, 41, 42). Another agonist, SR12418, which is modified based on the chemical structure of SR9009, showed higher efficiency in boosting the level of REV-ERBα and inhibiting the expression of IL-17A and NLRP3 in THP-1 cells (43). In addition, some other REV-ERBα agonists, such as GSK0999, GSK5072, and GSK2667, were synthesized with GSK2945 in the same study based on the chemical structure of GSK4112 and SR9009, and all of them showed inhibition of IL-6 upregulation induced by LPS on THP-1 cells (41). Currently, there are multiple REV-ERBα agonists synthesized, and most showed antiinflammation effects, which makes them promising candidates (39). However, applications of REV-ERBα agonists to other diseases are lacking and need verification of their efficacy. Our study showed that the REV-ERBα agonist is capable of attenuating CS-induced lung inflammation and abnormal EMT.

Although several new REV-ERBα agonists have been currently synthesized and evaluated to overcome the limitations of GSK4112 (poor solubility and short half-life), there are still challenges that may restrict the clinical translational value of REV-ERBα agonists. The in vivo half-life (oral gavage administration) of GSK2945 is approximately 2 hours, whereas that of SR9009 is 0.5 hours, and those of GSK0999, GSK5072, and GSK2667 are only 0.25 hours (41). It takes a total of 24 hours to clear the GSK2945 in serum, whereas it only takes 4 hours for SR9009, GSK0999, GSK5062, and GSK2667 (41). However, although there are new drugs synthesized based on molecular clock target, the half-life is still too short to satisfy the clinical needs. Frequent dosing is required because of the short half-life and fast clearance, and that is why we daily injected SR9009 i.p. before CS exposure for 10 days. Additionally, SR9009 also presents with side effects on cellular proliferation and metabolic progresses (44). Administration of SR9009 showed similar dose-dependent cytotoxicity, cellular proliferation, and mitochondrial respiration between WT and *REV-ERBα/β* double KO mouse embryonic stem cells (44). Short-duration time in biological system and off-target effects diminish the translational values of REV-ERBα agonists. However, multiple studies, including our previous reports, have identified that REV-ERBα is essential and critical in pulmonary injury, and REV-ERBα agonists possess the potential to combat lung inflammation and injury induced by environmental toxicants.

Further, RORα inhibitor, SR1001, presented a therapeutic effect in hypoxia-induced pulmonary hypertension (45), and the expression of RORα was upregulated after CS exposure as well as in patients with COPD/emphysema compared with healthy controls (46). The casein kinases (CKs) are required in the phosphorylation of PERs, which are also important in circadian rhythm regulation (47). The inhibitor of CK1δ/ε, PF670462, was found with antifibrotic ability either in TGF-β–induced cell differentiation or bleomycin-administered mice (48). Furthermore, CKs were found to interact with other targets in the Wnt signaling pathway, which is one of the key pathways that regulate EMT (49). Altogether, investigation of various circadian agonists in different lung disease models is necessary to understand the role of circadian clock targets and their therapeutic potentials for alleviating chronic inflammatory lung diseases. The combination of REV-ERBα agonist SR9009 with other steroidal versus nonsteriodal drugs could possibly be a new therapeutic strategy for chronic lung disease that needs to be tested.

In conclusion, REV-ERBα deficiency causes circadian clock dysfunction in the lungs, as well as abnormal mesenchymal transition induced by subchronic CS exposure. We found that REV-ERBα deletion in mice exposed to subchronic CS showed increased airspace enlargement and ECM remodeling compared with CS-exposed WT mice. Furthermore, inflammation and aberrant activation of EMT caused by acute CS exposure were partially abrogated by REV-ERBα agonist treatment. Taken together, our results show a promising role of circadian clock molecular target, REV-ERBα in management of chronic lung diseases associated with EMT dysregulation. The REV-ERBα agonist, SR9009 treatment showed potential effects against lung inflammation or inhibiting mesenchymal transition. Targeting multiple circadian clock molecules using selective agonists/antagonists (e.g., SR9009, and KL001) could be a novel approach for treating chronic lung disease associated with mesenchymal transition and abnormal ECM remodeling, such as COPD and lung fibrosis.

## Methods

For a description of experimental details, please refer to the Supplemental Information. All cellular and molecular analysis details, including inflammatory cell and cytokine measurement, Western blotting, quantitative reverse transcription PCR (qRT-PCR), NanoString quantification, H&E staining, Gomori’s Trichrome (collagen) staining, and IHC staining are provided in the supplemental materials.

### Human lung tissues.

Lung tissue specimens from 14 subjects, including 7 nonsmokers and 7 smokers, were procured from the National Disease Research Interchange (NDRI) and provided by Vuokko L. Kinnula (Pulmonary Division, Department of Medicine, University of Helsinki and Helsinki University Hospital, Helsinki, Finland) (Supplemental Table 1). Lungs were homogenized in RIPA buffer, and protein concentration was determined by the Pierce BCA Assay Kit (23227; Thermo Fisher Scientific).

### Cell culture and treatment.

HFL-1 cells were purchased from ATCC, and cultured in DMEM: F12K medium (11320033; Thermo Fisher Scientific) with 10% FBS (10082147; Thermo Fisher Scientific), 1% Penicillin-Streptomycin-Glutamine (10378016; Thermo Fisher Scientific), and 1% Non-Essential Amino Acids (11140076, Thermo Fisher Scientific). Before treatment, cells were serum deprived for 12 hours and then treated with 5 ng/mL TGF-β with or without 10 μM GSK4112 (3663; TOCRIS) for 3 days; or 0.1% CSE with or without 10 μM GSK4112 or 0.25% CSE for 2 days. CSE stock was always prepared freshly right before the treatment as described previously (13). In brief, CS (3R4F, University of Kentucky) was bubbling into 10 mL FBS-free DMEM: F12K medium (Phenol-red free) as 10% CSE stock. The 0.25% and 0.1% CSE working solutions were diluted from the stock CSE. Quality control of the CSE was based on the absorbance at 320 nm (1.00 ± 0.05 nm).

### Animals and CS exposure.

Adult C57BL/6 (WT) and *REV-ERBα* global KO (*REV-ERBα* KO) mice (male and female mice,4–5 months old) were housed in a 12-hour-light/12-hour-dark cycle in the inhalation core facility at the University of Rochester. *REV-ERBα*–KO mice were provided by Ronald Evans from the Salk Institute (La Jolla, California, USA) (19). WT mice were injected i.p. (veh/SR9009, 100 mg/kg body weight) with SR9009 dissolved in 15% cremophor (vehicle) 1 hour before CS exposure, SR9009 or vehicle were injected at ZT5 (11 am EST), every day for 10 days. Mice were exposed to CS for 10 days or 30 days; 2 hours/day with approximately 300 total particular matter (TPM, mg/m^3^), and exposed to CS for 4 months (chronic exposure) with approximately TPM 2 hours every day using the Baumgartner Jaeger mainstream smoking machine. Air group mice were i.p. injected with SR9009 or vehicle at the same time for 10 days without CS exposure. Body weight was recorded daily during the SR9009 treatment period. Mice were sacrificed 24 hours after the last exposure. Lungs were either snap-frozen for gene/protein analysis or fixed (10% formalin) for histological observation. BALF was collected for inflammatory measurement. Lung mechanical properties were analyzed 24 hours after the last exposure via Flexivent FX1 system (Scireq) as per the manufacturer’s instructions. Each measurement was performed 3 times per animal.

### Inflammatory cells and cytokine analysis.

Mice were euthanized with ketamine/xylazine, and lungs were lavaged with total 1.8 mL saline (0.6 mL/each time, for a total of 3 times). The BAL fluids were spun down to collect inflammatory cells, and supernatants were transferred for cytokine analysis. The cells were suspended and labeled with specific antibodies to identify the differentiated inflammatory cells. Specific cells were identified by Guava easyCyte flow cytometer (Millipore Sigma) and analyzed by Guava InCyte (50). Inflammatory cytokines were measured by Bio-plex Pro mouse cytokine 23-plex immunoassay kit (M60009RDPD; Bio-Rad), and plates were read through the Luminex Flexmap 3D (Luminex Corp).

### Protein isolation and Western blot.

Proteins were isolated from the snap-frozen lungs (approximately 30 mg) in RIPA buffer with protease inhibitor (78440; Thermo Fisher Scientific) with mechanical homogenization. An equal amount of protein (20 μg/lane) was used and then separated through 10% SDS-polyacrylamide electrophoresis (SDS-PAGE). The gels with protein samples were transferred onto a nitrocellulose membrane (1620112; Bio-Rad). The membranes were blocked with 5% non-fat milk at room temperature for 1 hour, followed by primary antibody incubation (overnight at 4°C). After primary antibody probing, membranes were washed with TBS-T, 10 minutes each for 4 times, and probed with goat anti-rabbit secondary antibody (1:5000, 1706515; Bio-Rad) for 1 hour at room temperature. Membranes were developed with Pierce ECL Western Blotting Substrate (32106; Thermo Scientific) and imaged by Bio-Rad ChemiDoc MP imaging system (Bio-Rad). The normalization was done based on β-actin (1:2500, ab20272; Abcam) or GAPDH (1:1000, ab9482; Abcam), and fold change was normalized to the WT air group or the air group treated with vehicle. See complete unedited blots in the supplemental material.

### RNA isolation.

First, snap frozen lung lobes were homogenized in QIAzol reagent (79306; Qiagen) mechanically. Lung homogenates were mixed with chloroform and vortexed for 10 seconds, followed by centrifugation at 15,000*g* for 15 minutes in 4°C. After centrifugation, aqueous phase was transferred to new RNase-free tubes and then mixed with isopropanol. The mixtures were kept at –20°C for 2 hours and then spun down at 20,637*g* for 15 minutes in 4°C, and the supernatant discarded. The RNA pellets were washed with 75% EtOH and spun down again at 20,637*g* for 15 minutes in 4°C. The EtOH was discarded and resuspended in RNA with RNase-free water. All of the RNA samples were cleaned up by RNeasy Plus Mini Kit (74136, Qiagen) based on the manufacturer’s protocol. RNA samples were stored at –80°C until analysis.

### NanoString quantification.

RNA samples isolated from lung lobes were quantified through NanoDrop spectrophotometer (ND-1000, NanoDrop Technologies), and 100 ng RNA samples were prepared for NanoString analysis. The customized panels from NanoString, including circadian genes and EMT genes, were used in this study. All the gene expressions were presented as normalized counts and measured by nCounter SPRINT Profiler (NanoString Technologies) and analyzed by nSolver 4.0 software.

### qRT-PCR.

RNA samples isolated from lung lobes were quantified by Nano-drop spectrophotometer (ND-1000, NanoDrop Technologies). RT was done by RT^2^ First Strand Kit (330401; Qiagen), and qRT-PCR was based on SYBR green expression master-mix (330509; Qiagen). qPCR was done by the Bio-Rad CFX96 qPCR instrument, and mRNA expression was determined by 2^-ΔΔCt^ methods with normalization using GAPDH as housekeeping control.

### H&E staining.

The staining protocol has been described in our previous study (51). Briefly, lung sections (5 μm) were deparaffinized with xylene and rehydrated with gradient percentage of ethanol (100%, 95%, and 70%). Then, sections were soaked in hematoxylin for 1 minute and 7% ammonia-water for 10 seconds. The sections were stained with eosin for 1 minute and rinsed in 95% ethanol for 1 minute. After staining, slides were dehydrated in 95% ethanol and 100% xylene. All slides were mounted for further analysis.

### Gomori’s Trichrome (collagen) staining.

The staining protocol has been described in our previous study (51). Lung sections were stained using the Gomori’s Trichrome staining kit that was commercially available (87020; Thermo Fisher Scientific) based on the manufacturer’s protocol. The slides were dehydrated and mounted for observation and Ashcroft scoring (52). The Ashcroft scoring was done based on the previous study in a blinded manner (5, 51).

### IHC staining.

The staining protocol has been described in our previous study (51). Lung sections (5 μm) were deparaffinized and rehydrated, and then the antigens unmasked with antigen retrieval solution (10x) (S1699; Dako). Sections were blocked with 10% normal goat serum and then incubated with primary antibodies at 4°C overnight. Then, slides were soaked in 0.3% hydrogen peroxide for 15 minutes, then washed with TBS. Secondary antibody (1:1000, ab7090; Abcam) was applied to the section at room temperature for 1 hour. Slides were developed with DAB Quanto Chromogen and Substrate (TA-125-QHDX; Thermo Fisher Scientific) and then counter stained with hematoxylin. Slides then dehydrated and were mounted for further analysis.

### Lung morphometry.

The Lm of airspace was measured through H&E-stained lung section (×20) by MetaMorph software (Molecular Devices). Pictures were taken randomly from 2–4 sections per slide in a blinded manner, more than 8 pictures were used for Lm calculation, and all of the pictures were used for analysis following manual threshold as described previously (53).

### Declarations.

Lung tissues were obtained from the NDRI and/or NIH Lung Tissue Research Consortium and/or provided by Vuokko L. Kinnula.

### Statistics.

The statistical differences among samples were analyzed through either 1-way ANOVA or 2-tailed Student’s *t* test in GraphPad Prism software (version 8.0). Results were presented as the mean ± SEM. A *P* value of less than 0.05 was considered significant.

### Study approval.

This study was performed according to the standards from the United States Animal Welfare Act, NIH. All of the animal experiments followed the protocol approved by the Animal Research Committee of the University of Rochester.

## Author contributions

QW, IKS, and IR conceived and designed the experiments. QW, JHL, TM, and IKS conducted the experiments. QW and TM analyzed the data. QW, JHL, TM, IKS, and IR wrote and revised the manuscript.

## Supplementary Material

Supplemental data

## Figures and Tables

**Table 1:**
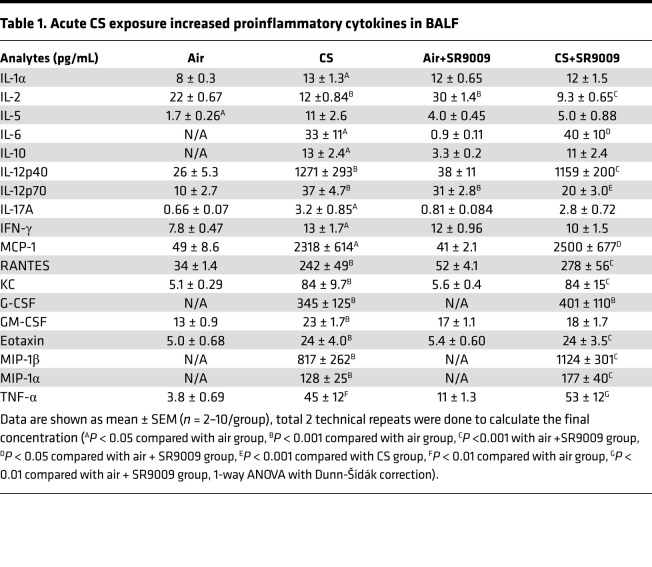
Acute CS exposure increased proinflammatory cytokines in BALF

## Version 1 05/20/2021

In-Press Preview

## Version 2 06/22/2021

Electronic publication

## References

[1] Blanco I (2019). Geographic distribution of COPD prevalence in the world displayed by Geographic Information System maps. Eur Respir J.

[2] Zhang L (2020). Epigenetic modifications and therapy in chronic obstructive pulmonary disease (COPD): an update review. COPD.

[3] Yao H (2013). SIRT1 redresses the imbalance of tissue inhibitor of matrix metalloproteinase-1 and matrix metalloproteinase-9 in the development of mouse emphysema and human COPD. Am J Physiol Lung Cell Mol Physiol.

[4] van der Vaart H (2004). Acute effects of cigarette smoke on inflammation and oxidative stress: a review. Thorax.

[5] Sundar IK (2015). Influenza A virus-dependent remodeling of pulmonary clock function in a mouse model of COPD. Sci Rep.

[6] Sohal SS, Walters EH (2013). Role of epithelial mesenchymal transition (EMT) in chronic obstructive pulmonary disease (COPD). Respir Res.

[7] Bartis D (2014). Epithelial-mesenchymal transition in lung development and disease: does it exist and is it important?. Thorax.

[8] Hou W (2019). Cigarette smoke induced lung barrier dysfunction, EMT, and tissue remodeling: a possible link between COPD and lung cancer. Biomed Res Int.

[9] Eurlings IM (2014). Cigarette smoke extract induces a phenotypic shift in epithelial cells; involvement of HIF1α in mesenchymal transition. PLoS One.

[10] Vu T (2016). Effect of cigarette smoking on epithelial to mesenchymal transition (EMT) in lung cancer. J Clin Med.

[11] Sundar IK (2015). Circadian molecular clock in lung pathophysiology. Am J Physiol Lung Cell Mol Physiol.

[12] Mohawk JA (2012). Central and peripheral circadian clocks in mammals. Annu Rev Neurosci.

[13] Sundar IK (2017). The nuclear receptor and clock gene REV-ERBα regulates cigarette smoke-induced lung inflammation. Biochem Biophys Res Commun.

[14] Yao H (2015). Disruption of sirtuin 1-mediated control of circadian molecular clock and inflammation in chronic obstructive pulmonary disease. Am J Respir Cell Mol Biol.

[15] Dibner C (2010). The mammalian circadian timing system: organization and coordination of central and peripheral clocks. Annu Rev Physiol.

[16] Hwang JW (2014). Circadian clock function is disrupted by environmental tobacco/cigarette smoke, leading to lung inflammation and injury via a SIRT1-BMAL1 pathway. FASEB J.

[17] Cunningham PS (2020). The circadian clock protein REVERBα inhibits pulmonary fibrosis development. Proc Natl Acad Sci U S A.

[18] Papagiannakopoulos T (2016). Circadian rhythm disruption promotes lung tumorigenesis. Cell Metab.

[19] Cho H (2012). Regulation of circadian behaviour and metabolism by REV-ERB-α and REV-ERB-β. Nature.

[20] Zhang Z (2019). Genome-wide effect of pulmonary airway epithelial cell-specific *Bmal1* deletion. FASEB J.

[21] Pariollaud M (2018). Circadian clock component REV-ERBα controls homeostatic regulation of pulmonary inflammation. J Clin Invest.

[22] Cao Q (2017). Circadian clock cryptochrome proteins regulate autoimmunity. Proc Natl Acad Sci U S A.

[23] Ehlers A (2018). BMAL1 links the circadian clock to viral airway pathology and asthma phenotypes. Mucosal Immunol.

[24] Gibbs JE (2012). The nuclear receptor REV-ERBα mediates circadian regulation of innate immunity through selective regulation of inflammatory cytokines. Proc Natl Acad Sci U S A.

[25] Delerive P (2001). The orphan nuclear receptor RORα is a negative regulator of the inflammatory response. EMBO Rep.

[26] Narasimamurthy R (2012). Circadian clock protein cryptochrome regulates the expression of proinflammatory cytokines. Proc Natl Acad Sci U S A.

[27] Curtis AM (2015). Circadian control of innate immunity in macrophages by miR-155 targeting Bmal1. Proc Natl Acad Sci U S A.

[28] Christ P (2018). The circadian clock drives mast cell functions in allergic reactions. Front Immunol.

[29] Saito A (2018). TGF-β signaling in lung health and disease. Int J Mol Sci.

[30] Wang Y (2017). Upregulation of circadian gene ‘hClock’ contribution to metastasis of colorectal cancer. Int J Oncol.

[31] Dong C (2016). Regulation of transforming growth factor-beta1 (TGF-β1)-induced pro-fibrotic activities by circadian clock gene BMAL1. Respir Res.

[32] Balsalobre A (1998). A serum shock induces circadian gene expression in mammalian tissue culture cells. Cell.

[33] Hoyle NP (2017). Circadian actin dynamics drive rhythmic fibroblast mobilization during wound healing. Sci Transl Med.

[34] Chang J (2020). Circadian control of the secretory pathway maintains collagen homeostasis. Nat Cell Biol.

[35] Chong NW (2006). Circadian clock genes cause activation of the human PAI-1 gene promoter with 4G/5G allelic preference. FEBS Lett.

[36] Scheer FA, Shea SA (2014). Human circadian system causes a morning peak in prothrombotic plasminogen activator inhibitor-1 (PAI-1) independent of the sleep/wake cycle. Blood.

[37] Chen W-D (2015). Circadian CLOCK mediates activation of transforming growth factor-β signaling and renal fibrosis through cyclooxygenase 2. Am J Pathol.

[38] Shen W (2020). SR9009 induces a REV-ERB dependent anti-small-cell lung cancer effect through inhibition of autophagy. Theranostics.

[39] Wang S (2020). Targeting REV-ERBα for therapeutic purposes: promises and challenges. Theranostics.

[40] Solt LA (2012). Regulation of circadian behaviour and metabolism by synthetic REV-ERB agonists. Nature.

[41] Trump RP (2013). Optimized chemical probes for REV-ERBα. J Med Chem.

[42] Zhang T (2018). REV-ERBα regulates CYP7A1 through repression of liver receptor homolog-1. Drug Metab Dispos.

[43] Amir M (2018). REV-ERBα regulates T(H)17 cell development and autoimmunity. Cell Rep.

[44] Dierickx P (2019). SR9009 has REV-ERB-independent effects on cell proliferation and metabolism. Proc Natl Acad Sci U S A.

[45] Li C (2020). Protective effects of resveratrol and SR1001 on hypoxia-induced pulmonary hypertension in rats. Clin Exp Hypertens.

[46] Shi Y (2012). Retinoic acid-related orphan receptor-α is induced in the setting of DNA damage and promotes pulmonary emphysema. Am J Respir Crit Care Med.

[47] Lee H-m (2011). The period of the circadian oscillator is primarily determined by the balance between casein kinase 1 and protein phosphatase 1. Proc Natl Acad Sci U S A.

[48] Keenan CR (2018). Casein kinase 1δ/ε inhibitor, PF670462 attenuates the fibrogenic effects of transforming growth factor-β in pulmonary fibrosis. Front Pharmacol.

[49] Thorne CA (2010). Small-molecule inhibition of Wnt signaling through activation of casein kinase 1α. Nat Chem Biol.

[50] Wang Q (2020). E-cigarette-induced pulmonary inflammation and dysregulated repair are mediated by nAChR α7 receptor: role of nAChR α7 in SARS-CoV-2 Covid-19 ACE2 receptor regulation. Respir Res.

[51] Wang Q (2020). Prenatal exposure to electronic-cigarette aerosols leads to sex-dependent pulmonary extracellular-matrix remodeling and myogenesis in offspring mice. Am J Respir Cell Mol Biol.

[52] Ashcroft T (1988). Simple methodof estimating severity of pulmonary fibrosis on a numerical scale. J Clin Pathol.

[53] Sundar IK (2018). Genetic ablation of histone deacetylase 2 leads to lung cellular senescence and lymphoid follicle formation in COPD/emphysema. FASEB J.

