# Supplementary material for: Molecular clock REV-ERBα regulates cigarette smoke–induced pulmonary inflammation and epithelial-mesenchymal transition
Source: JCI Insight. 2021 Jun 22;6(12):e145200. doi: 10.1172/jci.insight.145200 (PMC8262497; doi:10.1172/jci.insight.145200)
Supplement: Supplemental data [file jciinsight-6-145200-s149.pdf]

Supplementary information

Molecular Clock REV-ERBα regulates Cigarette Smoke-Induced Pulmonary Inflammation And Epithelial-Mesenchymal-Transition

Qixin Wang[1], Isaac K. Sundar[2], Joseph H. Lucas[1], Thivanka Muthumalage[1], and Irfan Rahman[1]*

[1]Department of Environmental Medicine, School of Medicine and Dentistry, University of Rochester Medical Center, Rochester, NY, USA

[2]Department of Internal Medicine, Division of Pulmonary, Critical Care and Sleep Medicine, University of Kansas Medical Center, Kansas City, KS, USA

Address for Correspondence:

*Irfan Rahman, Ph.D.
Department of Environmental Medicine
University of Rochester Medical Center
Box 850, 601 Elmwood Avenue
Rochester 14642, NY, USA
E-mail: irfan_rahman@urmc.rochester.edu

Supplementary information

Methods

Human lung tissues

Lung tissue specimens from 14 subjects, including 7 non-smokers and 7 smokers, were procured from National Disease Research Interchange (NDRI) and generously provided by Dr. Vuokko L. Kinnula (**Table S1**). Lungs were homogenized in RIPA buffer, and protein concentration was determined by the Pierce BCA Assay Kit (Cat#: 23227, ThermoFisher Scientific).

Cell culture and treatment

Human fetal lung fibroblast (HFL-1) was purchased from ATCC, and cultured in DMEM: F12K medium (Cat#:11320033, ThermoFisher Scientific) with 10%FBS (Cat#:10082147, ThermoFisher Scientific), 1% Penicillin-Streptomycin-Glutamine (Cat#: 10378016, ThermoFisher Scientific), and 1% Non-Essential Amino Acids (Cat#: 11140076, ThermoFisher Scientific). Before treatment, cells were serum-deprived for 12hrs and then treated with 5 ng/mL TGF-β with or without 10 μM GSK4112 (Cat#: 3663, TOCRIS) for 3 days; or 0.1% CSE with or without 10 μM GSK4112 or 0.25% CSE for 2 days. CSE stock was prepared freshly right before the treatment, and preparation was described in our previous study (1). In brief, cigarette smoke (3R4F, University of Kentucky, KY) was bubbling into 10mL FBS-free DMEM: F12K medium (Phenol-red free) as 10% CSE stock. The 0.25% and 0.1% CSE working solutions were diluted from the stock CSE. Quality control of the CSE was based on the absorbance at 320nm (1.00 ± 0.05).

Animals and Cigarette smoke (CS) exposure

Adult C57BL/6 (WT) and *Rev-erbα* global KO (*Rev-erbα* KO) mice (male and female mice, 4-5-month-old) were housed in a 12:12 light/dark cycle in the inhalation core facility at the University of Rochester. *Rev-erbα* KO mice were generously provided by Ronald Evans, PhD, from Salk Institute (La Jolla, CA) (2). WT mice were injected intra-peritoneally (i.p. veh/SR9009, 100 mg/kg body weight) with SR9009 dissolved in 15% cremophor (vehicle) 1h before CS exposure, SR9009 or vehicle were injected at ZT5 (11 am), every day for 10 days. Mice were exposed to CS for 10 days, 30 days or 4months; 2 h/day with ~300 total particular matter (TPM, mg/m^3) using the Baumgartner Jaeger mainstream smoking machine. Air group mice were i.p. injected with SR9009 or vehicle at the same time for 10 days without CS exposure. Bodyweight was recorded daily during the SR9009 treatment period. Mice were sacrificed 24 hrs after the last exposure; lungs were either snap-frozen for gene/protein analysis, or fixed (10% formalin) for histological observation; bronchoalveolar lavage fluid (BALF) was collected for inflammatory measurement.

Inflammatory cells and cytokine analysis

Mice were euthanized with ketamine/xylazine, and lungs were lavaged with total 1.8 mL saline (0.6 mL/each time, for a total of 3 times). The BAL fluids were spun down to collect inflammatory cells, and supernatants were transferred for cytokine analysis. The cells were suspended in 1 mL PBS and counted by trypan blue via the Bio-Rad cell counter. Re-suspended cells (1×10^5) were labeled with specific antibodies to identify the differentiated inflammatory cells. All cells were blocked with anti-mouse CD16/32 (Cat# 50-163-432, Fisher Scientific), and labeled with F4/80 PE ‑ conjugated antibody for macrophages (Cat# 123109, BioLegend), LY6B.2 Alexa fluor488 ‑ conjugated antibody for neutrophils (Cat# NBP213077AF488, Novus Biologicals), PE-Cyanine7 antibody for CD4a+ T-lymphocytes (Cat# 25-0041-82, Fisher Scientific), and APC conjugated Monoclonal Antibody for CD8a+ T-Lymphocytes (Cat# 17-0081-82, Fisher

Scientific). Specific cells were identified by Guava® easyCyte™ flow cytometer (Millipore Sigma) and analyzed by Guava® InCyte (3). Inflammatory cytokines were measured by Bio-plex Pro mouse cytokine 23-plex immunoassay kit (Cat#: M60009RDPD, Bio-Rad) based on the instruction manual, plates were read through the Luminex Flexmap 3D (Luminex Corp).

Protein isolation and Western blot

Proteins were isolated from the snap-frozen lungs (~30 mg) in RIPA buffer with protease inhibitor (Cat#: 78440, ThermoFisher Scientific) with mechanical homogenization. The homogenates were centrifuged at 15,000 g for 30 min at 4°C, and supernatants were collected for immunoblotting, and protein concentrations were determined by Pierce BCA Assay Kit (Cat#: 23227, ThermoFisher Scientific). An equal amount of protein (20 µg/lane) was used and then separated through 10% SDS-polyacrylamide electrophoresis (SDS-PAGE). The gels with protein samples were transferred onto a nitrocellulose membrane (Cat# 1620112, Bio-Rad), and proteins were confirmed by Ponceau S staining (Cat#: BP103-10, Fisher Scientific). Membranes were washed with Tris-buffered saline containing 0.1% Tween 20 (TBS-T) until Ponceau S disappeared. The membranes were blocked with 5% non-fat milk at room temperature for 1 h, followed by primary antibodies incubation (overnight at 4°C), anti-TGFβ1 (1:1000, ab92486, Abcam) www.labome.com/product/Abcam/ab92486.html and www.citeab.com/antibodies/1903011-ab92486-anti-tgf-beta-1-antibody reacting with human and mouse with 42kDa-50kDa; anti-Vimentin (1:1000, ab92547, Abcam); anti-COL1A1 (1:1000, NBP1-30054, Novus Biologicals); anti-Snail-slug (1:1000, ab180714, Abcam); anti-MMP9 (1:1000, ab38898, Abcam), anti-MMP2 (1:1000, ab92536, Abcam), anti-MMP12 (1:1000, NBP2-67344, Novus) anti-E-cadherin (1:1000, 3195S, Cell Signaling Technology); anti-ZO-1 (1:1000, ab96587, Abcam); anti-p53 (1:1000, ab131442, Abcam); anti-PAI-1(1:1000, ab66705, Abcam); anti-RORα (1:1000, ab60134, Abcam); anti-NR1D1 (1:1000, ab174309, Abcam) ; anti-BMAL1 (1:1000, ab93806, Abcam); Anti-CLOCK (1:1000, ab3517, Abcam) were used in this study.

After primary antibody probing, membranes were washed with TBS-T, 10 min each for 4 times, and probed with Goat-anti-Rabbit Secondary antibody (1:5000, #1706515, BioRad) for 1 h at room temperature. After secondary antibody incubation, membranes were washed with TBS-T 4 times, 10 min each, then developed with Pierce™ ECL Western Blotting Substrate (Cat#: 32106, Thermo Scientific™). Bio-Rad ChemiDoc MP imaging system (Bio-Rad Laboratories, Hercules, CA, USA) was used to capture the images from membrane as well as for quantification of band intensity. The normalization was done based on β-actin (1:2500, ab20272, Abcam) or GAPDH (1:1000, ab9482, Abcam), and fold-change was normalized to the WT air group, or air group treated with vehicle.

RNA isolation

Snap frozen lung lobes were selected and homogenized in QIAzol reagent (Cat#:79306, Qiagen) mechanically. Lung homogenates were mixed with chloroform and vortexed for 10s, followed by centrifugation at 12,000 RPM, for 15 min in 4°C. After centrifugation, the top phase (aqueous phase) was transferred to new RNase-free tubes and then mixed with an equal volume of isopropanol. The mixtures were kept at -20°C for 2 h, for RNA condensation. After 2 h incubation, RNA samples were spun down at 15,000 RPM, for 15 min in 4°C, and the supernatant was discarded without disturbing the pellets. The RNA pellets were washed with 75% EtOH, and spun down again at 15,000 RPM, for 15 min in 4°C. The EtOH was carefully removed and the pellets were re-suspended with RNase-free water. All the RNA samples were then cleaned up and their gDNA eliminated by RNeasy Plus Mini Kit (Cat#: 74136, Qiagen) based on the manufacture's protocol. RNA samples were stored at -80°C until analysis.

Nanostring quantification

RNA samples isolated from lung lobes were quantified through Nano-drop spectrophotometer (ND-1000, NanoDrop Technologies, Wilmington, DE, USA), and 100 ng RNA samples were

prepared for nanostring analysis. The customized panels from nanostring, including circadian genes and epithelial-mesenchymal-transition (EMT) genes, were used in this study. Briefly, all reagents were thawed at room temperature, and the master mix was added into reporter codeset. Aliquot 8 µL of mixtures into each sample tubes followed by 5 µL of the total RNA samples. Finally, added 2 µL of capture probeset, the contents were mixed thoroughly, and incubated for 16 h at 65°C for RNA hybridization. After RNA hybridization, samples were diluted to 30 µL and loaded into Nanostring cartridge. All the gene expressions were presented as normalized counts and measured by nCounter SPRINT Profiler (NanoString Technologies, Inc.), and analyzed by nSolver 4.0 software.

Quantitative-RT-PCR

RNA samples isolated from lung lobes were quantified by Nano-drop spectrophotometer (ND-1000, NanoDrop Technologies, Wilmington, DE, USA).Total 500 ng RNA samples were prepared for reverse transcription and real-time PCR quantification. Reverse transcription was done by RT^2 First Strand Kit (Cat# 330401, Qiagen), and qRT-PCR was based on SYBR green expression master-mix (Cat# 330509, Qiagen). Primers used here was purchased from Bio-Rad: SERPINE1 (Mouse, qMmuCID0027303), VIM (Mouse, qMmuCED0046651), CDH1 (Mouse, qMmuCEP0052623), FN1 (Mouse, qMmuCEP0054113), SANI1 (Mouse, qMmuCID0024342), SNAI2 (Mouse, qMmuCED0046072), MMP2 (Mouse, qMmuCED0047200), GAPDH (Mouse, qMmuCEP0039581), ACTA2 (Human, qHsaCIP0028813), COL1A1 (Human, qHsaCEP0050510), Fn1 (Human, qHsaCEP0050873), and GAPDH (Human, qHsaCEP0041396). The qPCR conditions include 95°C for 10 min, then 40 cycles of 15 secs at 95°C followed by 1 min at 60°C, fluorescence intensity was measured at the end of 60°C incubation. A melting curve was carried for determination of the quality of the cDNA amplification. Quantitative PCR was done by the Bio-Rad CFX96 qPCR instrument, and the

expression level of mRNA was determined by $2^{-\Delta\Delta Ct}$ methods with normalization using GAPDH as housekeeping control.

Hematoxylin and Eosin (H&E) staining

Lung sections (5 µm) were deparaffinized, rehydrated with xylene, and gradient percentage of ethanol (100%, 95%, and 70%). Lung section was washed with water after rehydration, and soaked in hematoxylin for 1 min, and rinsed with running water for 5 min. Slides were dipped into 7% ammonia-water for 10s, and washed with running water for 5 min; then slides were soaked into 95% ethanol for 1 min. Slides were stained with eosin for 1 min and rinsed in 95% ethanol for 1 min. After staining, slides were dehydrated in 95% and 100% ethanol for 3 min twice and then in xylene for 3 min, 3 time. All slides were mounted with Permount for light microscopy observation and lung morphometry analysis.

Gomori Trichrome (collagen) staining

Lung sections (5 µm) were deparaffinized, rehydrated with xylene and a gradient percentage of ethanol (100%, 95%, and 70%). Lung section was washed with water after rehydration. Lung sections were stained using the Gomori's Trichrome staining kit that was commercially available (Cat#: 87020, Thermo Fisher Scientific) based on the manufacturer's protocol. Slides were soaked in Bouin's fluid at 56°C for 1h, following by washing with water for 10 min. The slides were applied with working Wiegert’s iron hematoxylin solution for 10 min, and washed with water for 10 min. Then, slides were stained with trichrome for 15 min, and soaked in acetic acid for 1 min. The slides were washed with water for 30s, dehydrated in 95% ethanol and anhydrous alcohol for 1 min each. The slides were dehydrated and mounted for observation and Ashcroft scoring (4). The Ashcroft scoring was done based on the previous study in a blind manner (5, 6).

Immunohistochemistry (IHC) staining

Lung sections (5 µm) were deparaffinized and rehydrated before immunohistochemical staining. Antigen retrieval solution (10x) (Cat#: S1699, Dako, Denmark) was used for antigen retrieval at 95°C for 30 min, slide stayed in solution until reached room temperature. Then, slides were washed with TBS+0.25% triton-100 (wash buffer) 2 times, 5 min each. Blocking of the section was done using 10% normal horse serum, and primary antibodies: anti-α-smooth-muscle-actin (1:1000, ab124964, Abcam); anti-Vimentin (1:250, ab92547, Abcam), anti-Snail-slug (1:200, ab180714,Abcam), anti-fibronectin (1:250, ab268020, Abcam) and anti-Col1A1 (1:50, NBP1-30054, Novus) were incubated at 4°C overnight. Then, slides were washed with wash buffer 2 times, 5 min each, and TBS 5 min. Slides were soaked in 0.3% hydrogen peroxide for 15 min, then washed with TBS for 10 min. Secondary antibody (1:1000, ab7090, Abcam) was applied to the section at room-temperature for 1h. Slides were washed with wash buffer and developed with DAB Quanto Chromogen and Substrate (Cat#: TA-125-QHDX, Thermo Fisher Scientific) for 10 mins at room temperature. Slides were washed with water and counter stained with hematoxylin, then dehydrated and mounted for further observation. Histological scoring was done based on a blind manner.

Lung morphometry

The mean linear intercept (Lm) of airspace was measured through H&E stained lung section (20x) by MetaMorph software (Molecular Devices, Sunnyvale, CA, USA). Pictures were taken randomly from 2 sections per slide in a blinded manner, >8 pictures were used for Lm calculation, and all the pictures were used for analysis followed manual threshold as described previously (7).

Statistical analysis

The statistical differences among samples were analyzed through either one-way ANOVA or student's t-test in GraphPad Prism software (Version 8.0, La Jolla, CA). Results were presented as the mean ± SEM, and $P < 0.05$ was considered as a significant difference.

References

1. Sundar IK, Rashid K, Sellix MT, and Rahman I. The nuclear receptor and clock gene REV-ERBα regulates cigarette smoke-induced lung inflammation. *Biochem Biophys Res Commun.* 2017;493(4):1390-5.
2. Cho H, Zhao X, Hatori M, Yu RT, Barish GD, Lam MT, et al. Regulation of circadian behaviour and metabolism by REV-ERB-α and REV-ERB-β. *Nature.* 2012;485(7396):123-7.
3. Wang Q, Sundar IK, Li D, Lucas JH, Muthumalage T, McDonough SR, et al. E-cigarette-induced pulmonary inflammation and dysregulated repair are mediated by nAChR α7 receptor: role of nAChR α7 in SARS-CoV-2 Covid-19 ACE2 receptor regulation. *Respir Res.* 2020;21(1):154.
4. Ashcroft T, Simpson JM, and Timbrell V. Simple method of estimating severity of pulmonary fibrosis on a numerical scale. *J Clin Pathol.* 1988;41(4):467-70.
5. Wang Q, Sundar IK, Blum JL, Ratner JR, Lucas JH, Chuang TD, et al. Prenatal Exposure to Electronic-Cigarette Aerosols Leads to Sex-Dependent Pulmonary Extracellular-Matrix Remodeling and Myogenesis in Offspring Mice. *Am J Respir Cell Mol Biol.* 2020;63(6):794-805.
6. Sundar IK, Ahmad T, Yao H, Hwang J-w, Gerloff J, Lawrence BP, et al. Influenza A virus-dependent remodeling of pulmonary clock function in a mouse model of COPD. *Sci Rep.* 2015;5(1):9927.
7. Sundar IK, Rashid K, Gerloff J, Rangel-Moreno J, Li D, and Rahman I. Genetic ablation of histone deacetylase 2 leads to lung cellular senescence and lymphoid follicle formation in COPD/emphysema. 2018;32(9):4955-71.

Table S1. **Patient information for human samples**

Group	Sex	Health Status	Group (smoking history)	Age (yr)
	Female	Normal	Non-smoker	47
	Male	Normal	Non-smoker	29
	Male	Normal	Ex-smoker	63
Non-smoker	Female	Normal	Non-smoker	34
	Male	Normal	Non-smoker	39
	Male	Normal	Ex-smoker	58
	Male	Normal	Ex-smoker	47
	Male	Normal	Current Smoker	67
	Female	Normal	Current Smoker	62
	Female	Normal	Current Smoker	40
Smoker	Female	Normal	Current Smoker	61
	Male	Normal	Current Smoker	41
	Male	Normal	Current Smoker	26
	Male	Normal	Current Smoker	55

Supplementary figures and legends

Fig S1

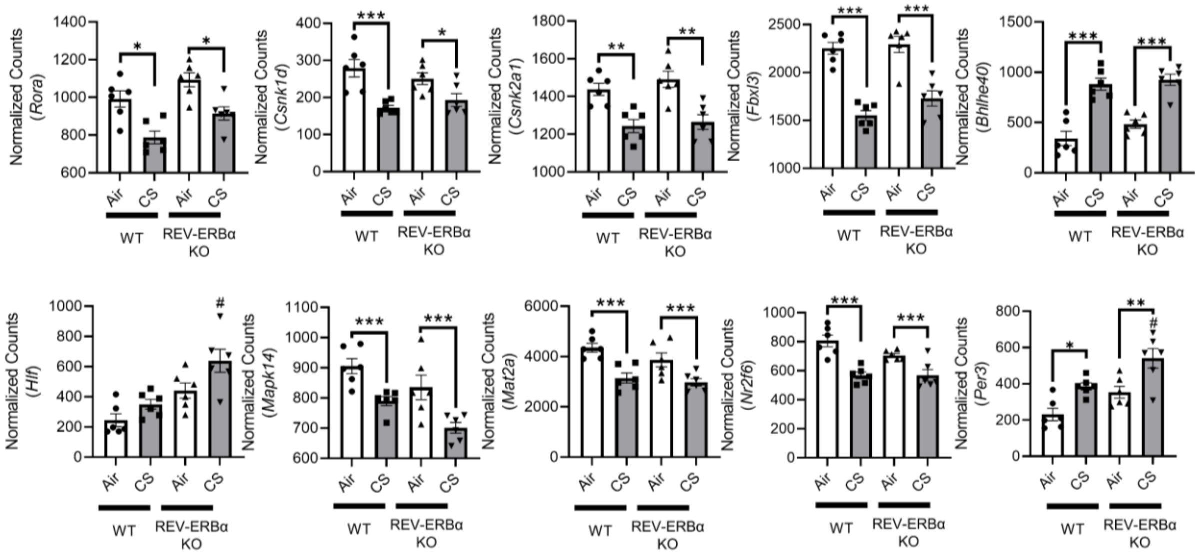

Figure S1 **Sub-chronic CS exposure affects circadian clock genes analyzed by NanoString**

Lungs from sub-chronic 30 days air and CS exposed mice were used for the isolation of total RNA. Our customized NanoString panel (circadian gene focused) was used to screen the potential targets via nCounter® SPRINT Profiler. Normalization of absolute RNA counts and data analysis were done by nSolver software. Data are shown as mean ± SEM. (n=6. * $P < 0.05$, ** $P < 0.01$, *** $P < 0.001$ between groups; $^{\#}$ $P < 0.05$ compared with CS exposed WT group, One-way ANOVA with Šidák correction).

Fig S2

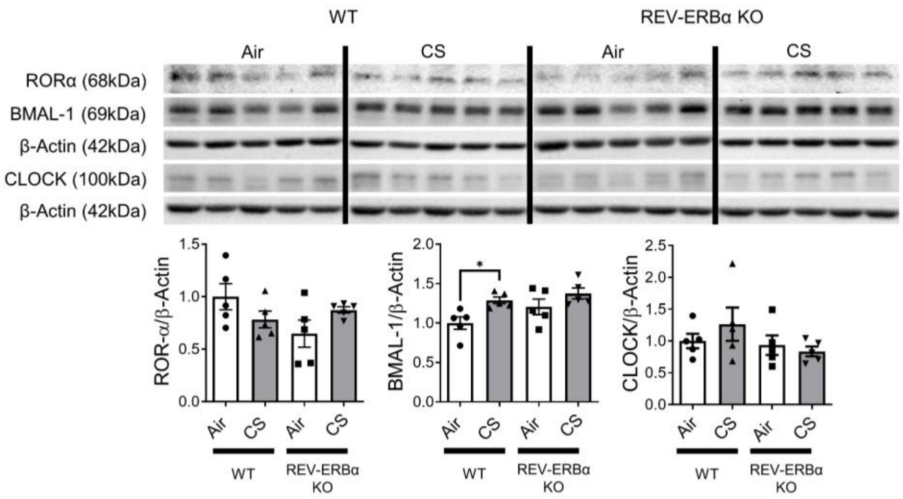

Figure S2 **Protein levels of circadian molecules were affected by sub-chronic CS exposure**

Lungs from sub-chronic 30 days air and CS exposed mice were homogenized and protein abundance were measured by Western blotting. Representative blot images are shown and relative protein fold change of RORα, BMAL1 and CLOCK are analyzed based on densitometry with β-actin as the endogenous control. RORα and BMAL1 were probed in the same membrane. Different groups were run on the same membrane, but were noncontiguous. Data are shown as mean ± SEM. (n=5. * $P < 0.05$, One-way ANOVA with Šidák correction).

Fig S3

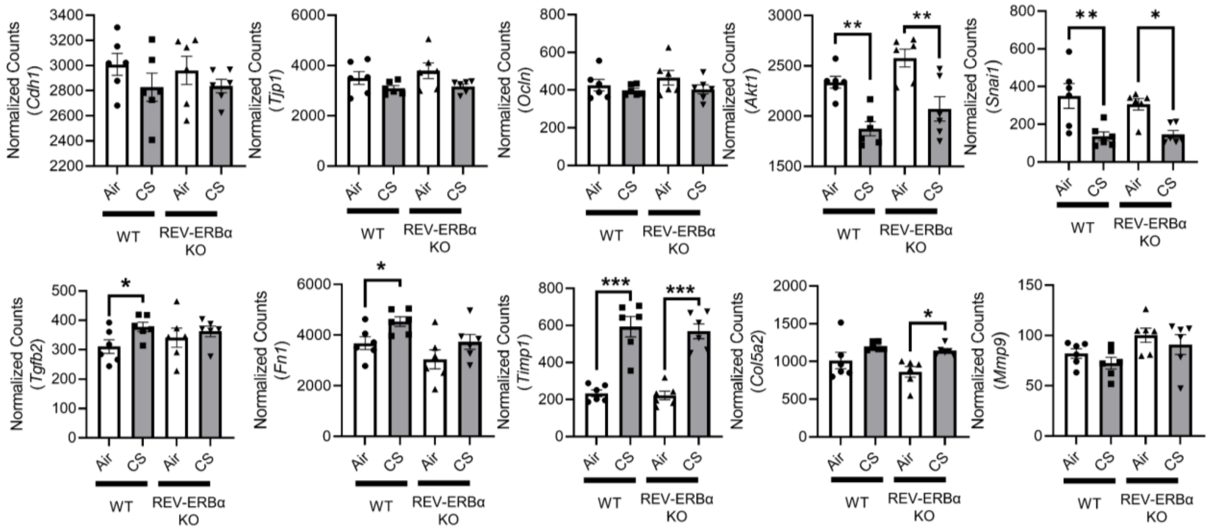

Figure S3 **Sub-chronic CS exposure affects gene expression of EMT markers analyzed by NanoString**

Lungs from sub-chronic 30 days air and CS exposed mice were used for the isolation of total RNA. Our customized NanoString panel (EMT gene focused) was used to screen the potential EMT targets via nCounter® SPRINT Profiler. Normalization of absolute RNA counts and data analysis were done by nSolver software. Data are shown as mean ± SEM. (n=6. * $P < 0.05$, ** $P < 0.01$, *** $P < 0.001$, One-way ANOVA with Šidák correction).

Fig S4

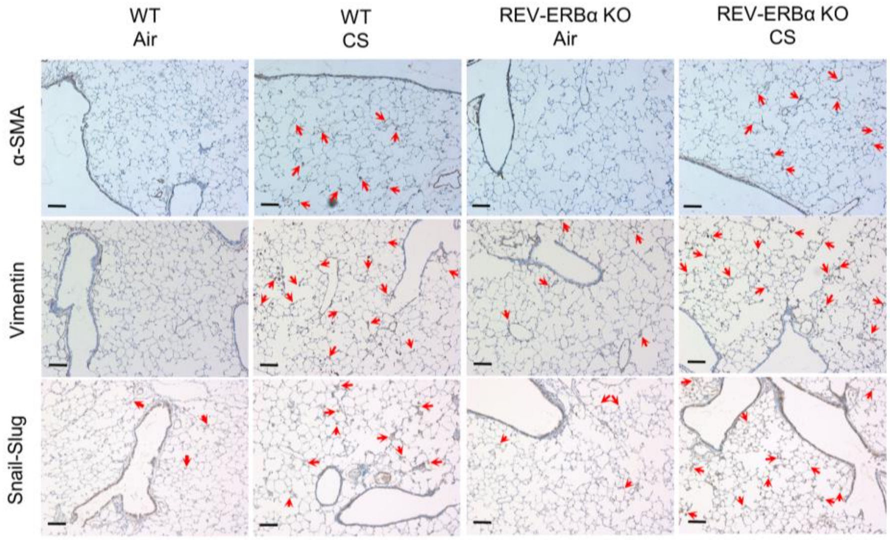

Figure S4 **Sub-chronic CS exposure demonstrates REV-ERBα-dependent change in activation of EMT markers in the lungs**

Sub-chronic CS exposure induced abnormal EMT activation in alveolar region as confirmed by immuno-histochemical staining specific for EMT markers such as αSMA, Vimentin, and Snail-Slug (10x). Abnormal protein accumulations were denoted by red arrows (Scale bar = 100 μm).

Fig S5

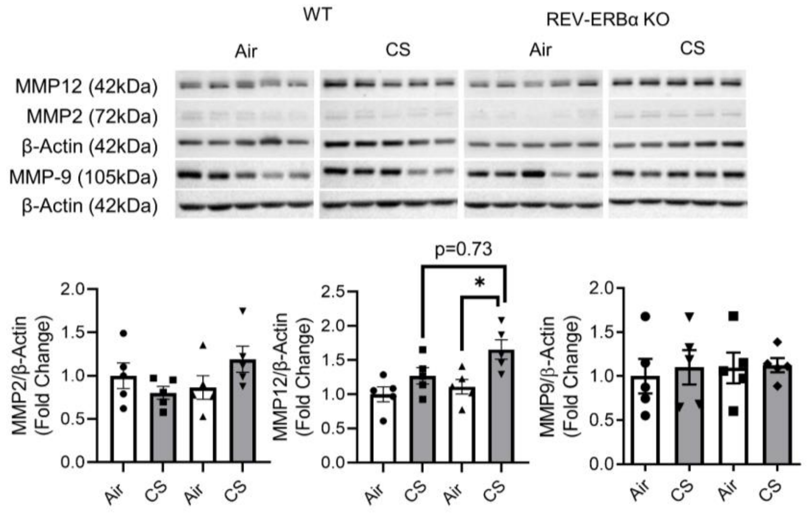

Figure S5 **Protein abundance of MMPs were affected by sub-chronic CS exposure**

Lungs from sub-chronic 30 days air and CS exposed mice were homogenized and protein abundance were measured by Western blotting. Representative blot images are shown and relative protein fold change of MMP2, MMP9, and MMP12, were analyzed based on densitometry with β-actin as the endogenous control. MMP12 and MMP2 were probed in the same membrane. Different groups were run on the same membrane, but were noncontiguous. Data are shown as mean ± SEM. (n=5. * $P < 0.05$, One-way ANOVA with Šidák correction).

Fig S6

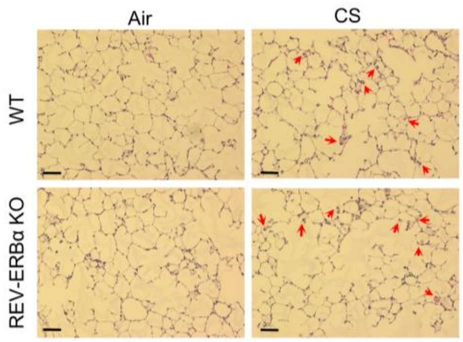

Figure S6 **Airspace enlargement and inflammation were induced by sub-chronic CS exposure**

Sub-chronic CS exposure (1 month) induced airspace enlargement and lung inflammation observed by H&E (20x) staining. CS exposure induced airspace enlargement was denoted by green arrows and inflammatory responses were denoted by red arrows (Scale bar = 100 μm).

Fig S7

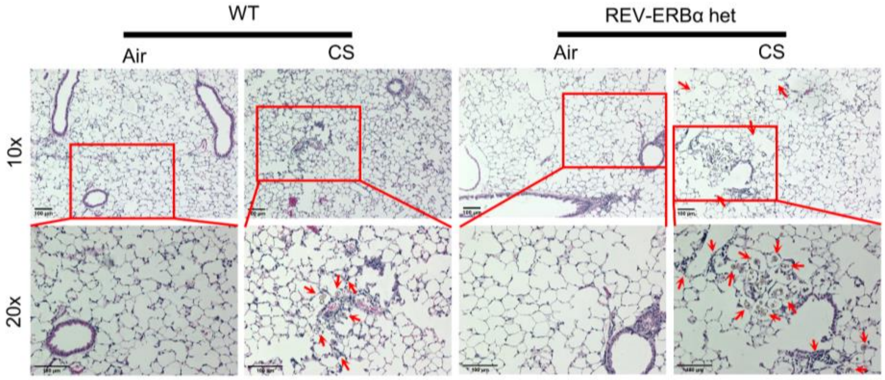

Figure S7 **Airspace enlargement and inflammation were induced by chronic CS exposure in REV-ERBα Het mice**

Chronic CS exposure (4 month) induced airspace enlargement and lung inflammation observed by H&E (10x and 20x) staining. CS exposure induced airspace enlargement was denoted by green arrows and inflammatory responses were denoted by red arrows (Scale bar =100μm).

Fig S8

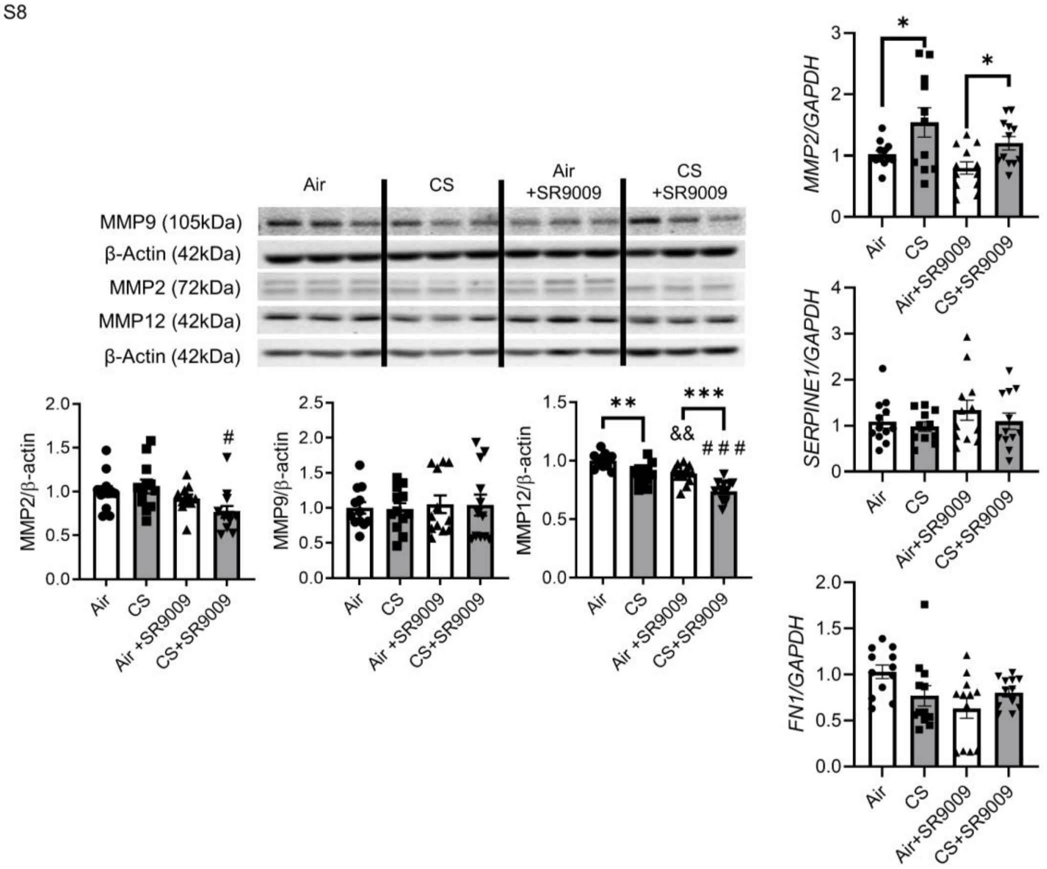

Figure S8 **Protein and gene expression of EMT markers were altered by sub-chronic CS exposure**

Lungs from sub-chronic (30 days) air and CS exposed mice were homogenized and protein abundance were measured by Western blotting. (A). Representative blot images are shown and relative protein fold change of MMP2, MMP9, and MMP12, were analyzed based on densitometry with β-actin as the endogenous control. MMP12 and MMP2 were probed in the same membrane. (B). Total RNA isolated from lung tissues, and transcript levels of specific target genes (*MMP2*, *SERPINE1*, and *FN1*) were analyzed by qRT-PCR. GAPDH was used as an endogenous control, and gene fold change was calculated by $2^{-\Delta\Delta Ct}$ method (total 2 technical repeats were done to calculate the final results). Data are shown as mean ± SEM. (n=11-12. * *P* < 0.05, ** *P* < 0.01, *** *P* < 0.001; $^{\&\&}$ *P*<0.01 compared to air group; $^{\#\#\#}$ *P*< 0.001 compared to CS group, One-way ANOVA with Šidák correction).

Fig S9

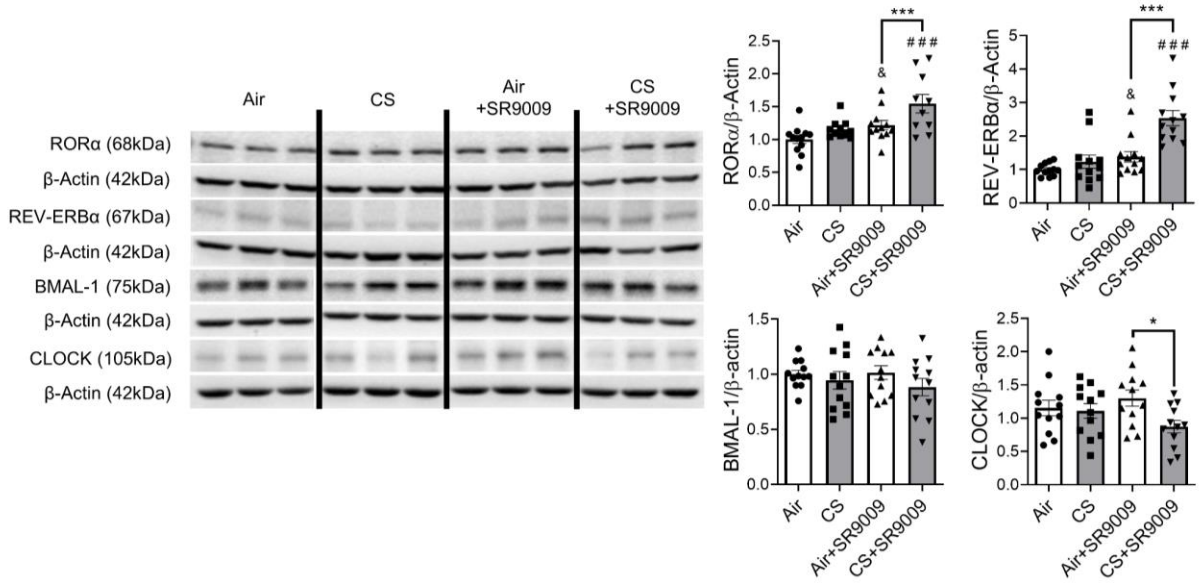

Figure S9 **Protein abundance of circadian molecules were affected by acute CS exposure** Lungs from acute 10 days air and CS exposed mice administered with or without veh/SR9009 were homogenized and protein abundance were measured by Western blotting. Representative blot images are shown and relative protein fold change of RORα, REV-ERBα, BMAL1, and CLOCK were analyzed based on densitometry with β-actin as the endogenous control. RORα and REV-ERBα were probed in the same membrane. Different groups were run on the same membrane, but were noncontiguous. Data are shown as mean ± SEM. (n=11-12. * $P < 0.05$, *** $P < 0.001$, One-way ANOVA with Šidák correction).

Fig S10

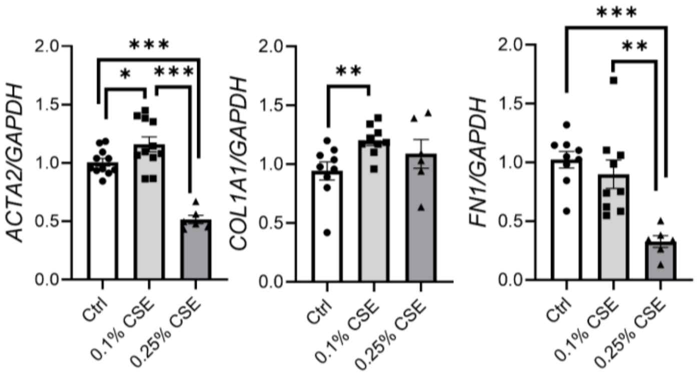

Figure S10 **High concentration of CSE inhibited fibroblast differentiation**

Human lung fibroblast (HFL-1) cells were treated with 0.1% and 0.25% CSE for 2 days. Total RNA was isolated from HFL-1 cells, and transcript levels of target genes (*ACTA2, COL1A1,* and *FN1*) were analyzed by qRT-PCR. GAPDH was used as an endogenous control for normalization. Data are shown as mean ± SEM. (n=6-11; * $P < 0.05$, ** $P < 0.01$, *** $P < 0.001$, between groups, paired student’s t-test).

Uncropped/unedited images for all blots and gels

Molecular Clock REV-ERBα regulates Cigarette Smoke-Induced Pulmonary Inflammation And Epithelial-Mesenchymal-Transition

Qixin Wang[1], Isaac K. Sundar[2], Joseph H. Lucas[1], Thivanka Muthumalage[1], and Irfan Rahman[1]*

[1]Department of Environmental Medicine, School of Medicine and Dentistry, University of Rochester Medical Center, Rochester, NY, USA

[2]Department of Internal Medicine, Division of Pulmonary, Critical Care and Sleep Medicine, University of Kansas Medical Center, Kansas City, KS, USA

Full unedited gel for Fig 1: PAI-1, Vimentin and β-Actin

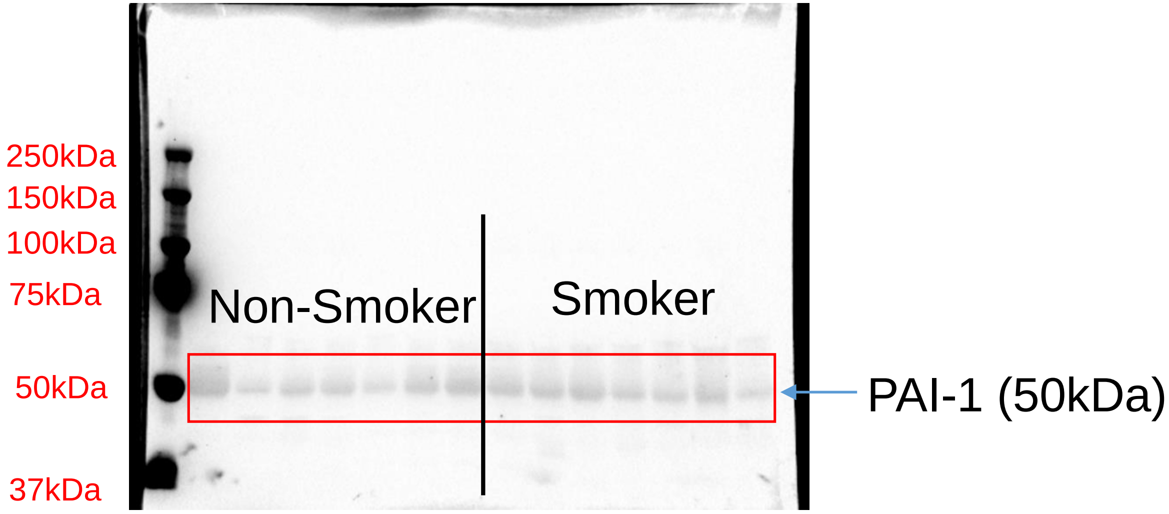

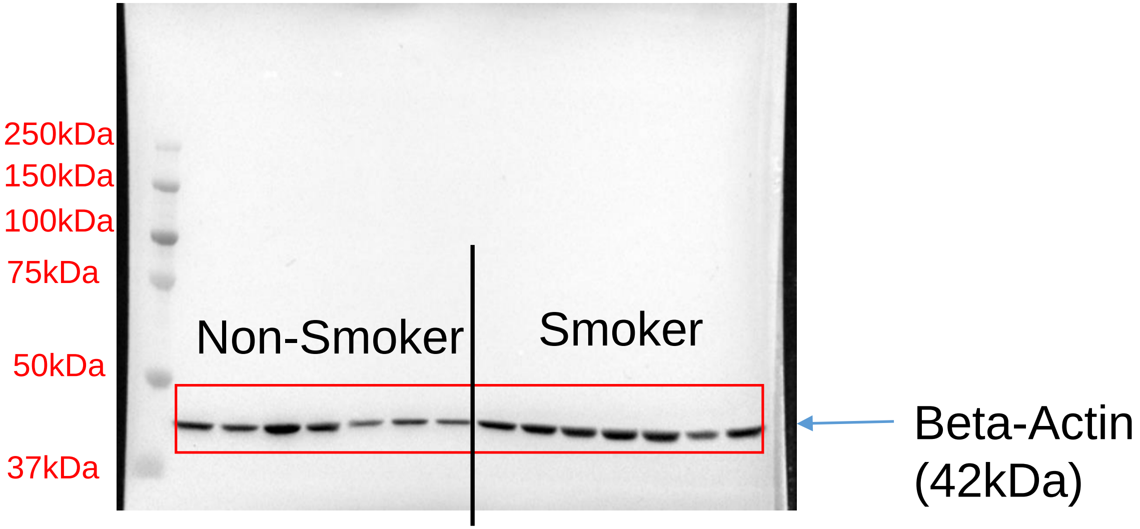

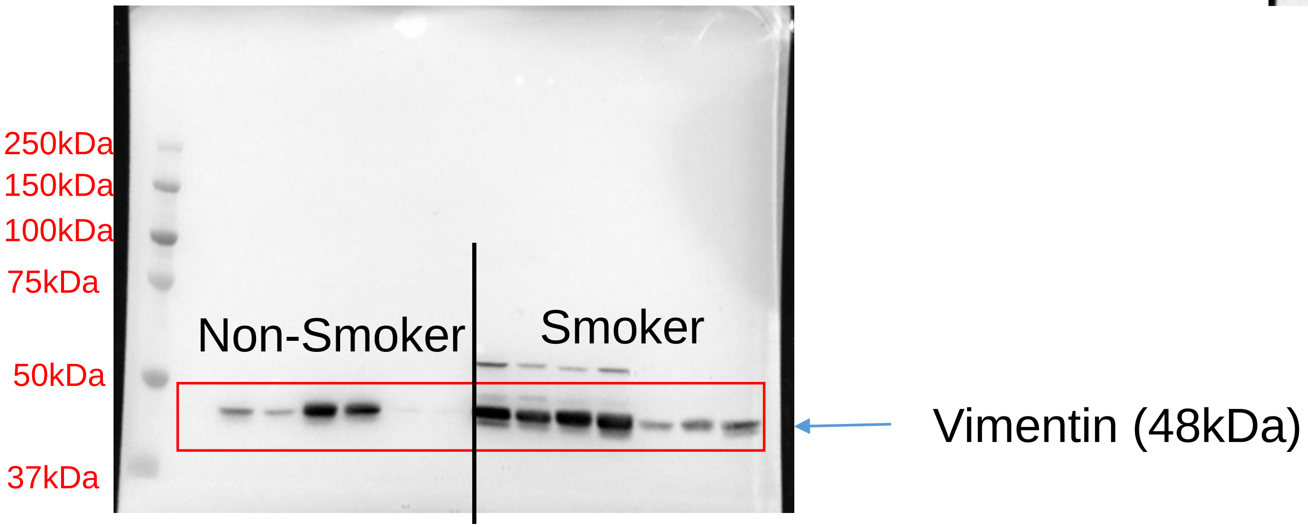

PAI-1 and Vimentin were probed on one membrane

Full unedited gel for Fig 1: COL1A1, TGFβ and β-Actin

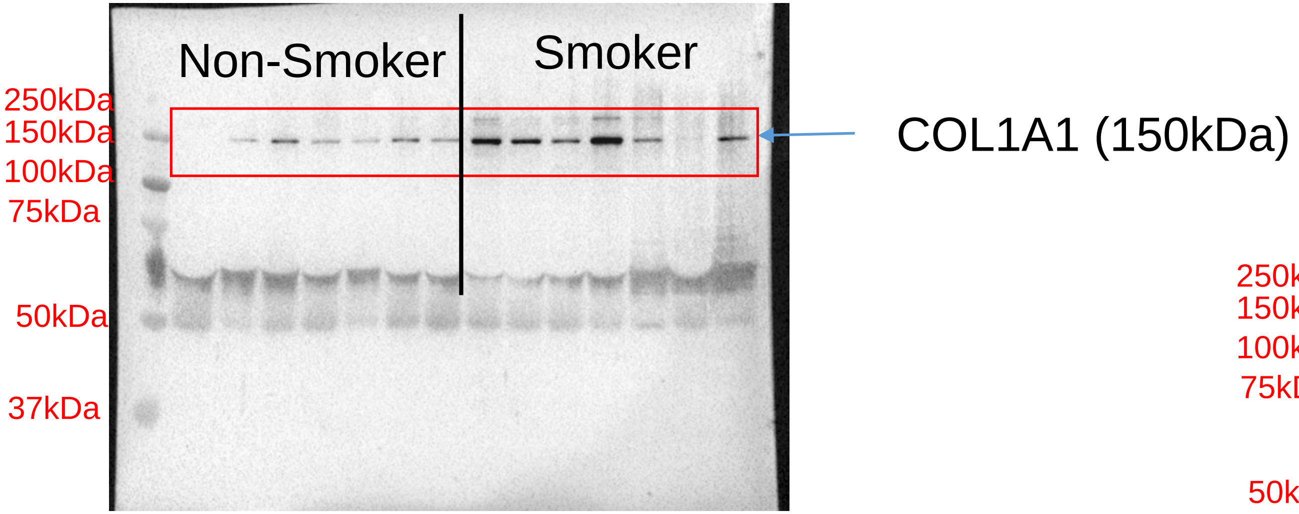

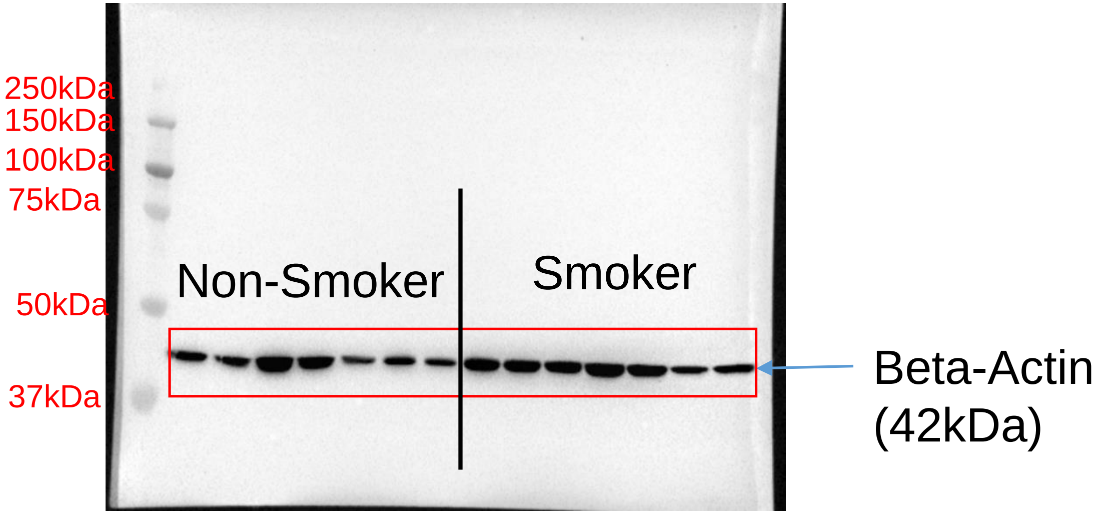

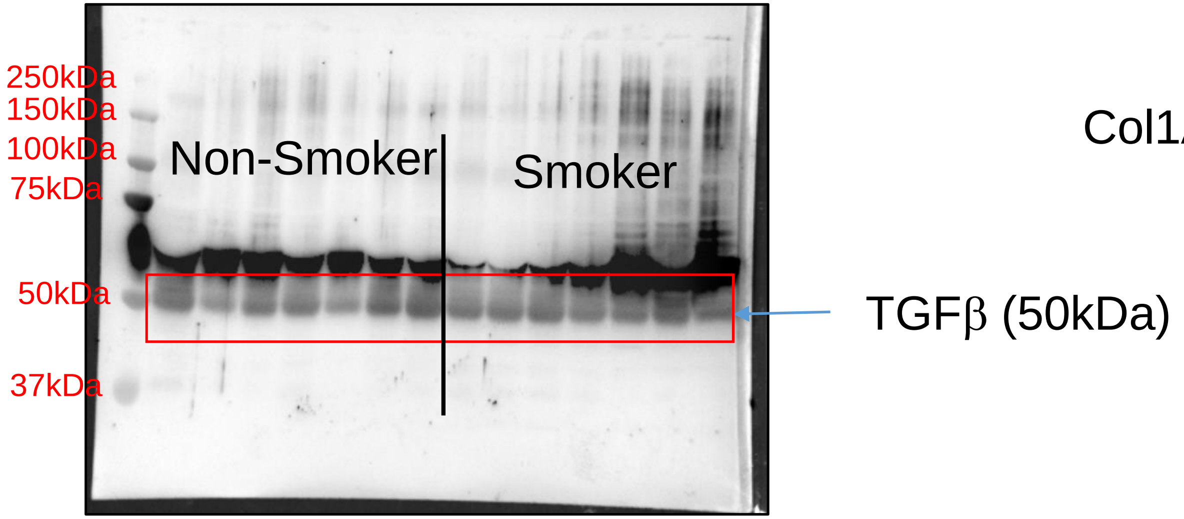

Col1A1 and TGF-beta were probed on one membrane

Full unedited gel for Fig 4A: Vimentin, COL1A1, and β-Actin

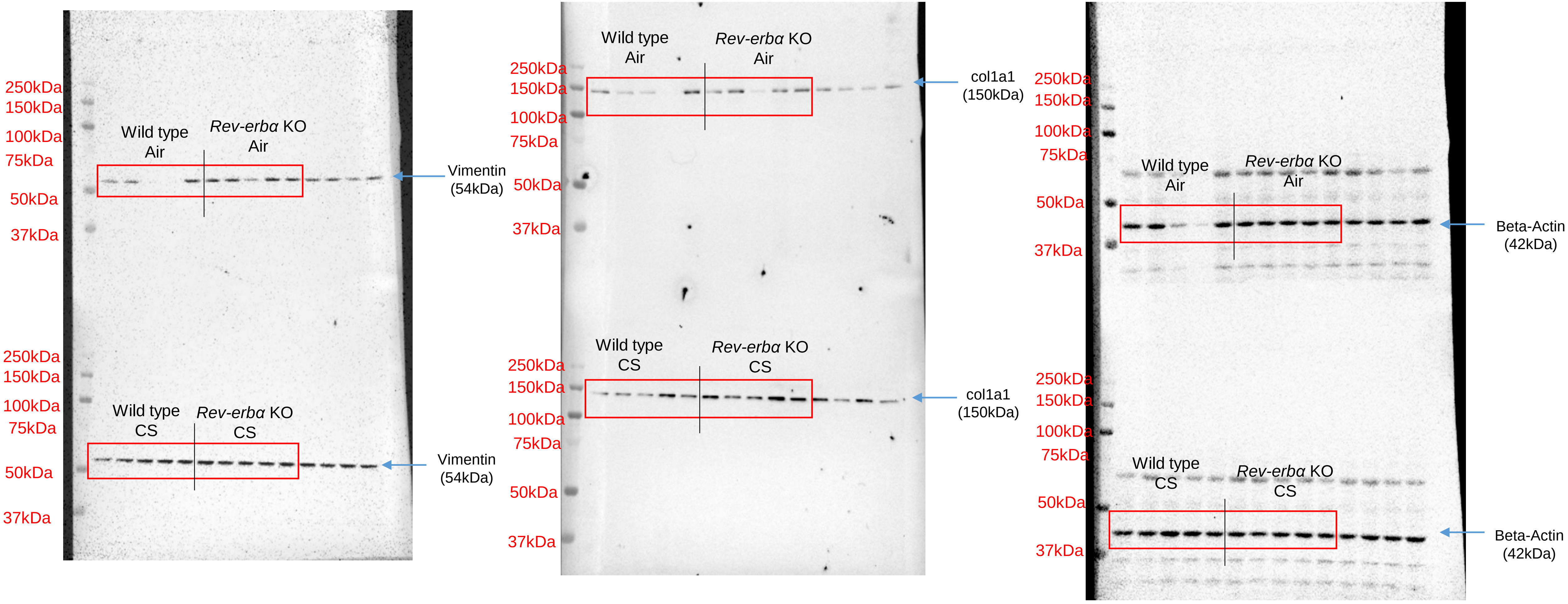

*Col1A1 and Vimentin were probed on one membrane

Full unedited gel for Fig 4A: TGFβ and β-Actin

Same beta actin were used for normalization of ZO-1 expression level as showed in Figure 5

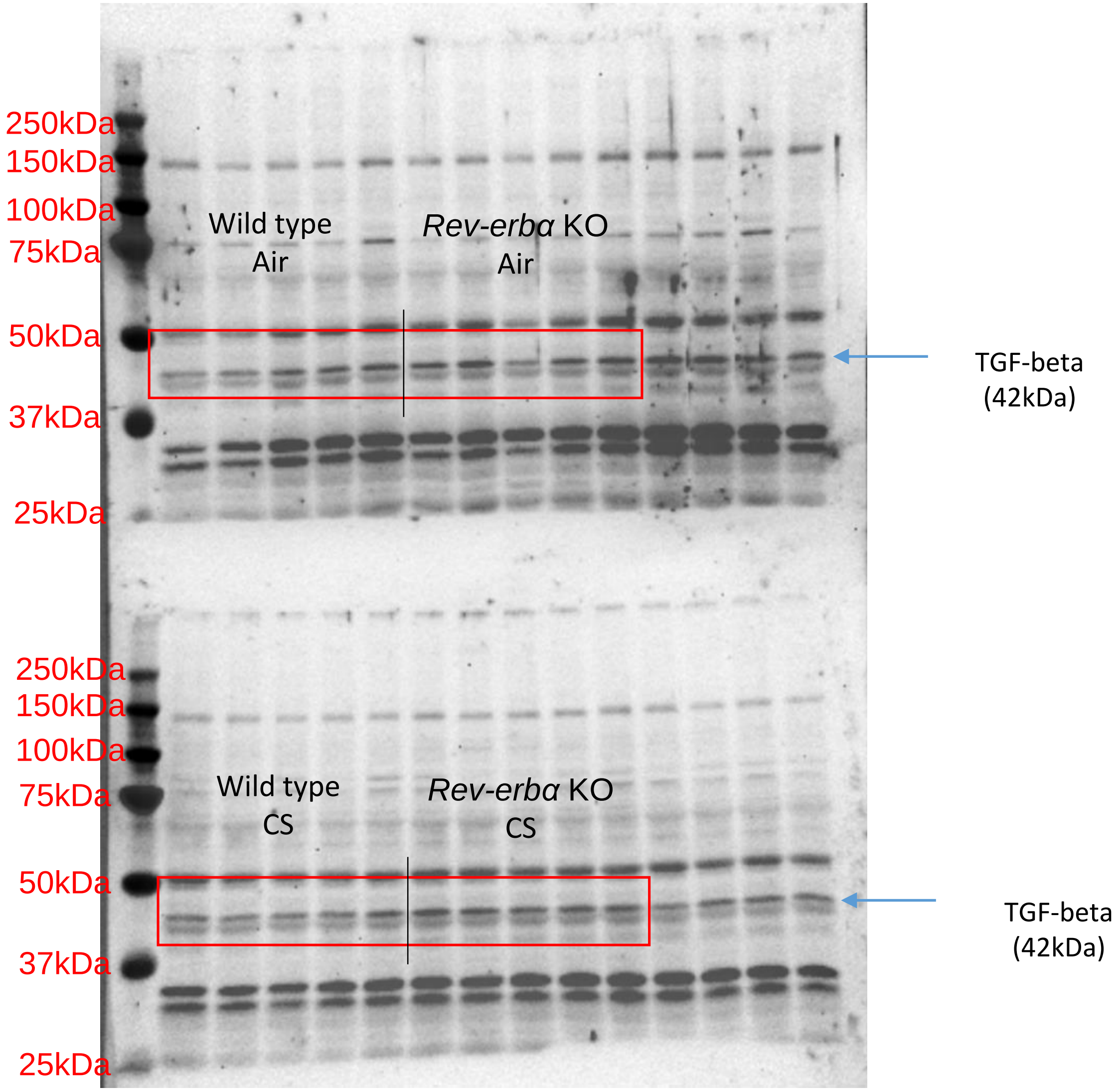

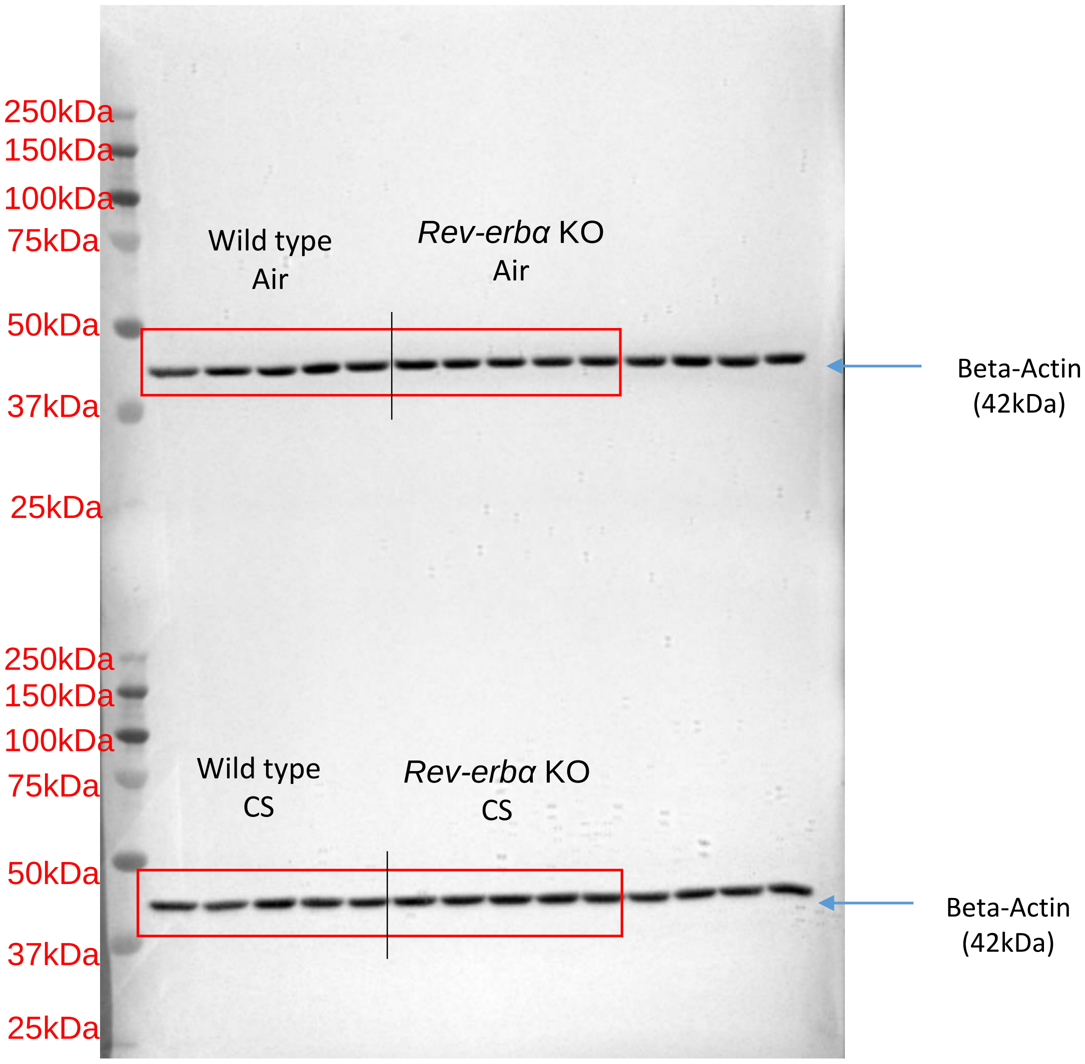

Full unedited gel for Fig 4A: Snail-Slug and β-Actin

Same beta actin were used for normalization of MMP2 and MMP12 expression level as showed in Figure S5

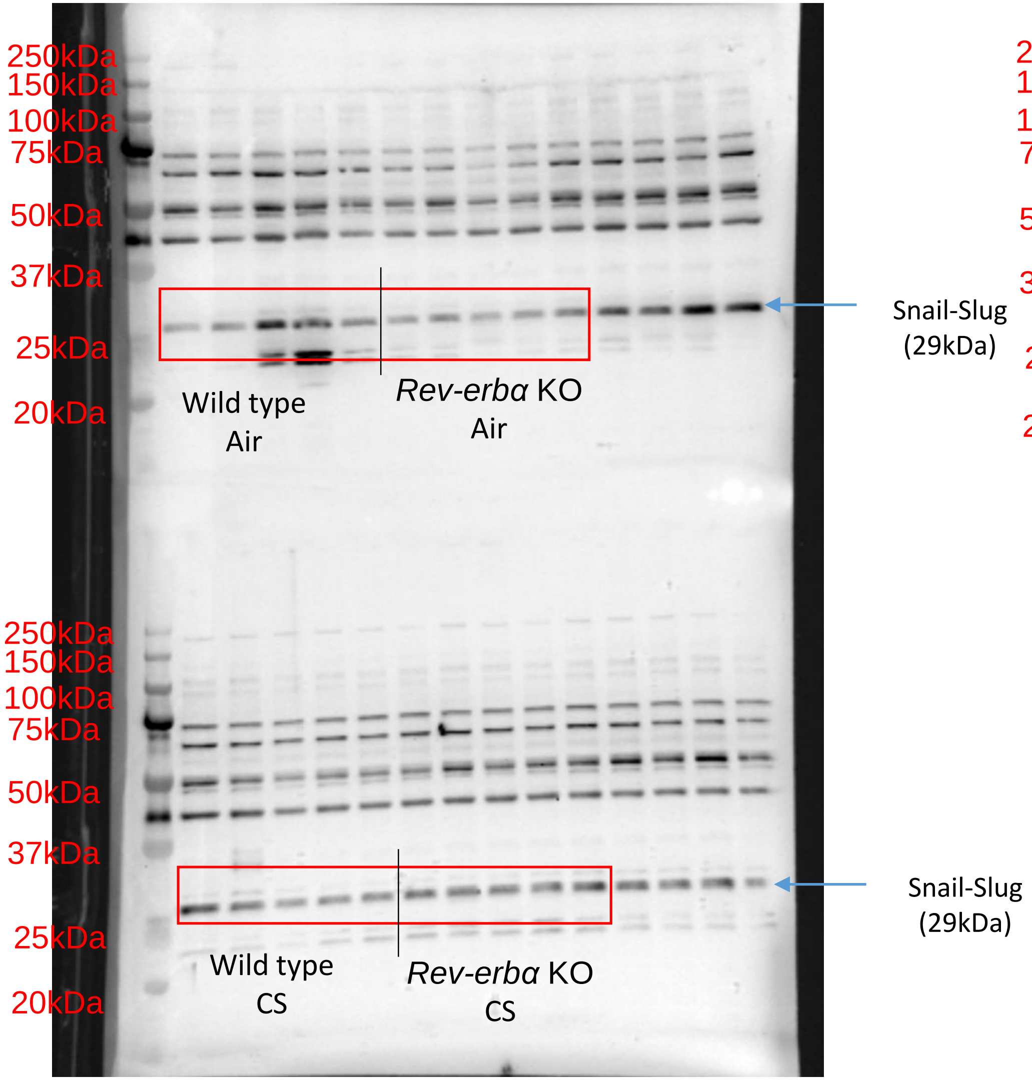

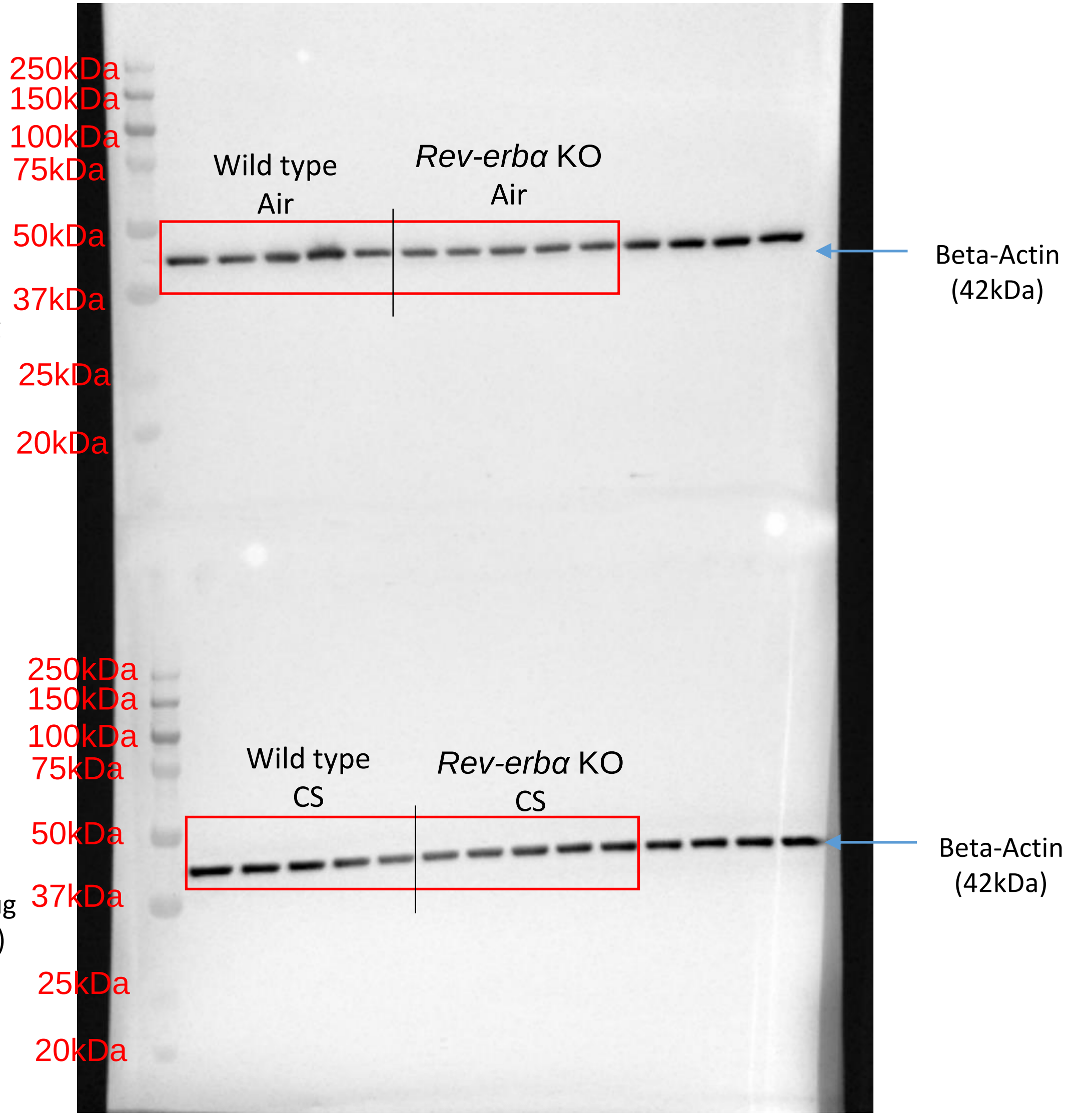

Full unedited gel for Fig 5: E-cadherin and β-Actin

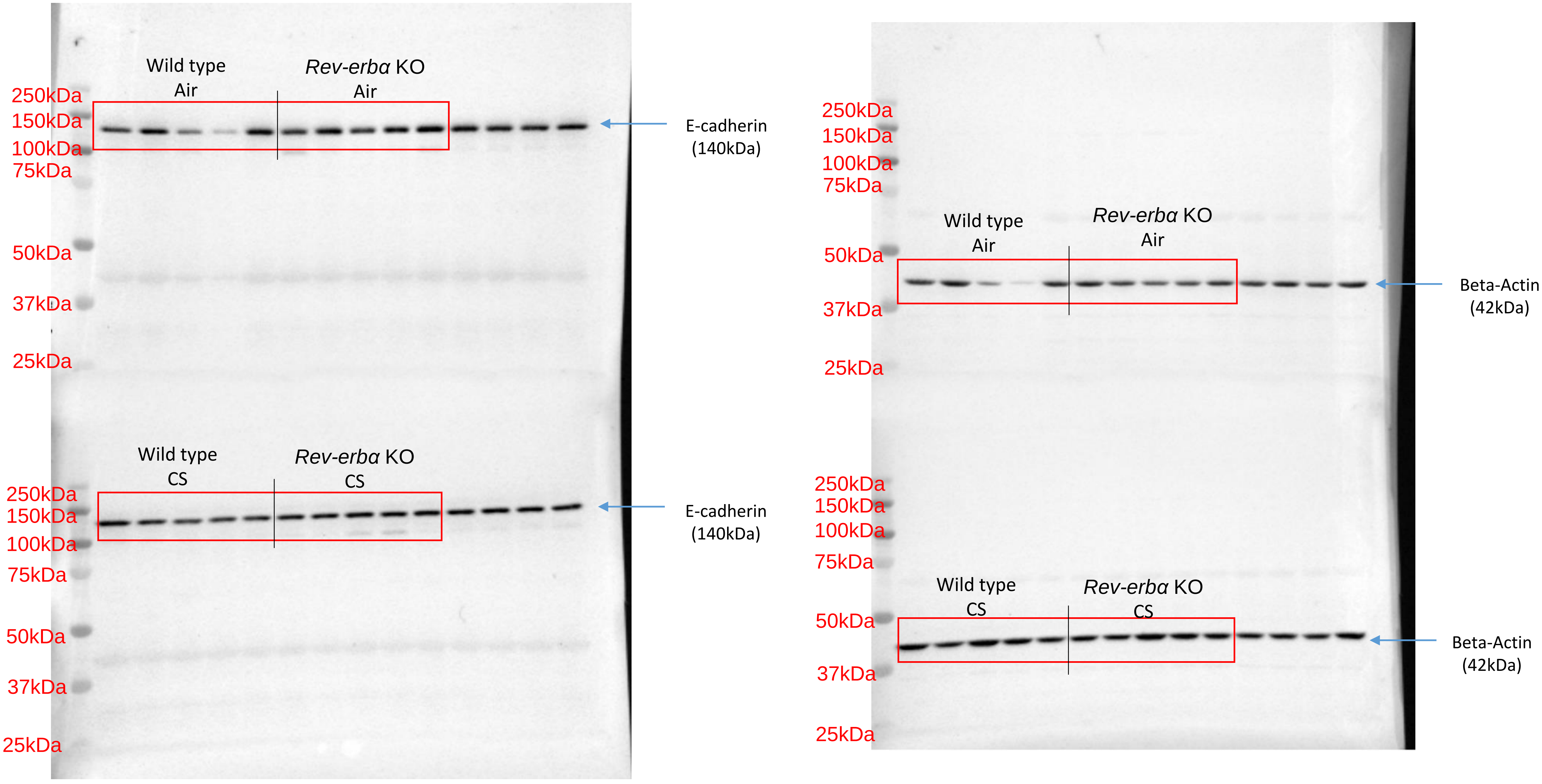

Full unedited gel for Fig 5: ZO-1 and β-Actin

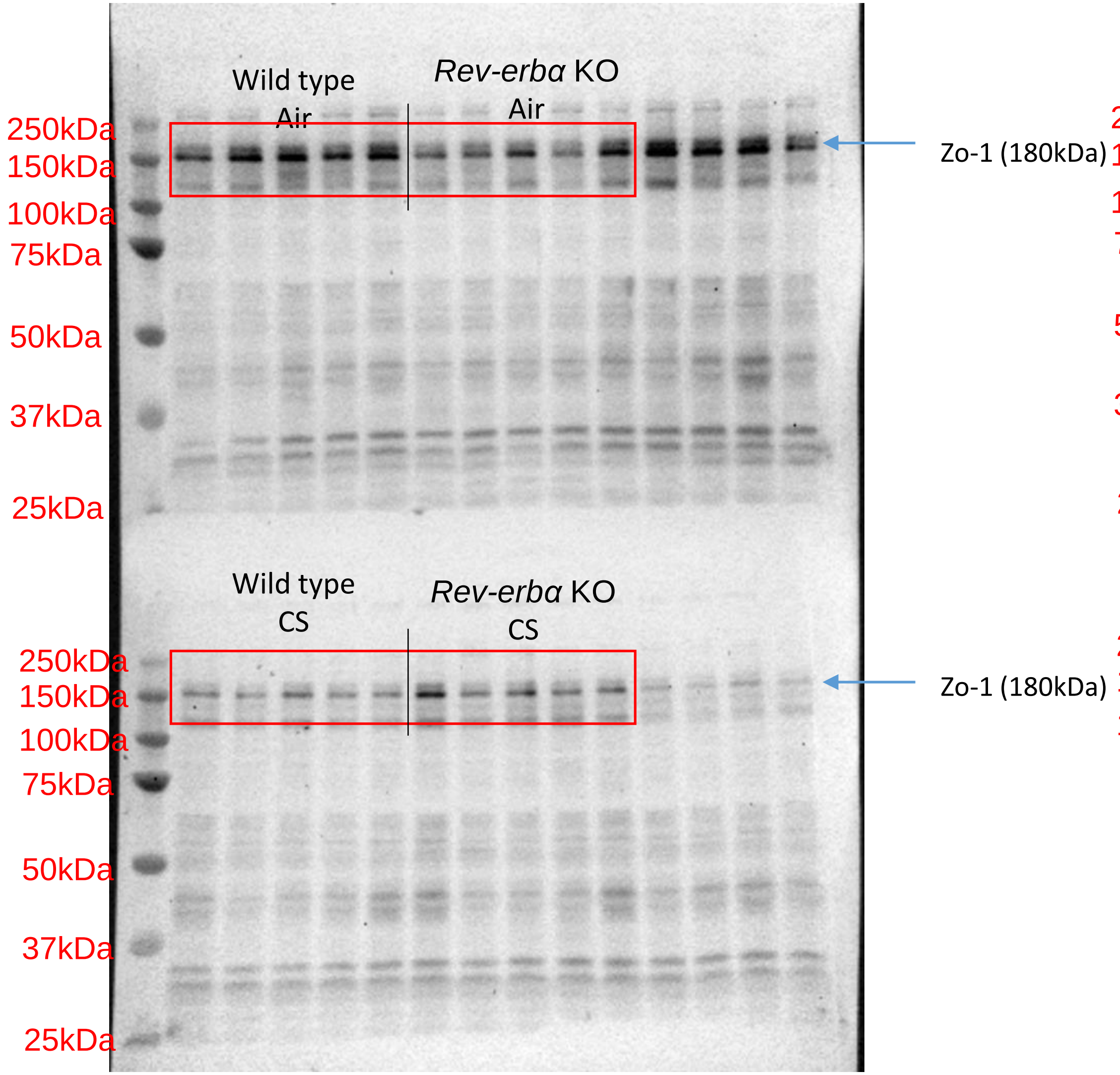

Same beta actin were used for normalization of TGF-beta expression level as showed in Figure 4A

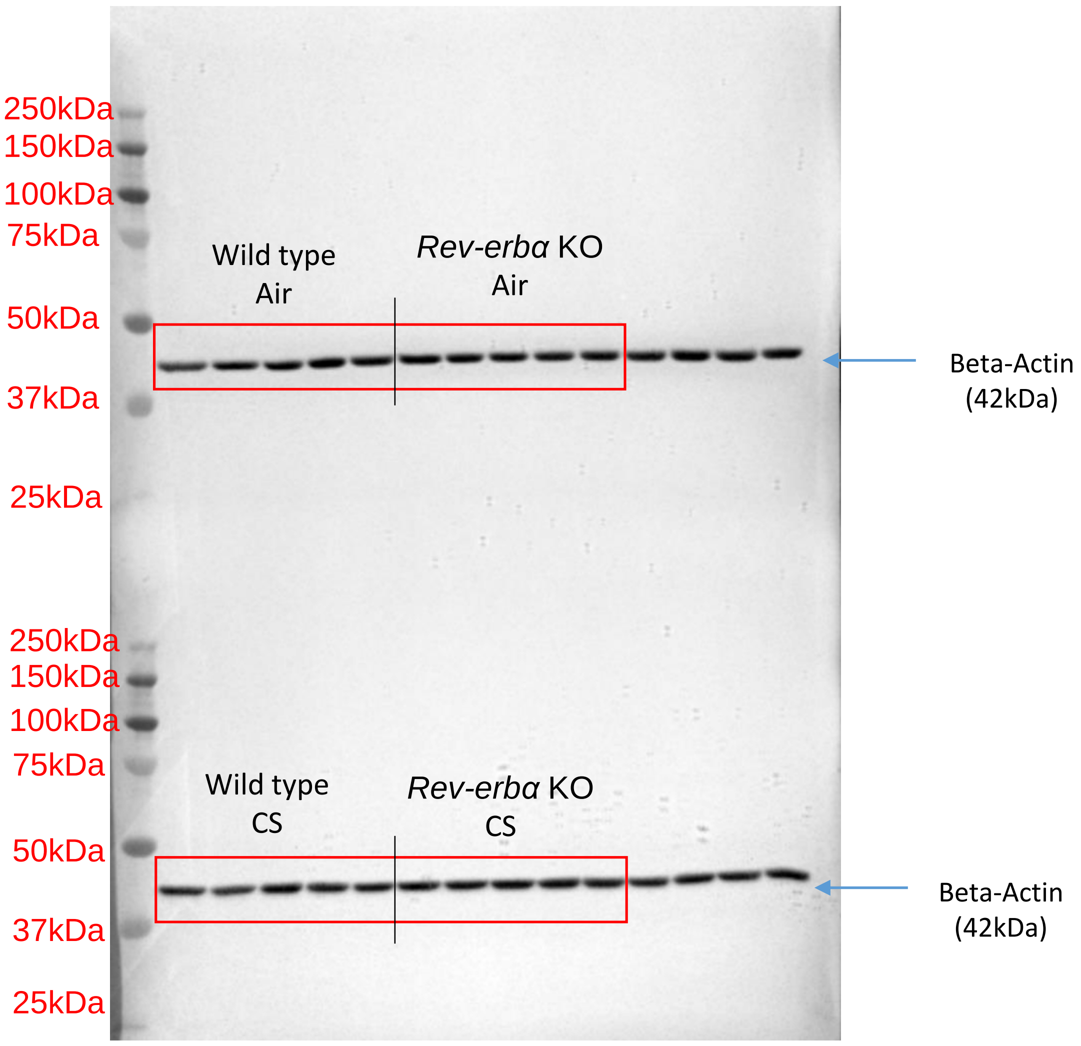

Full unedited gel for Fig 5: p53 and β-Actin

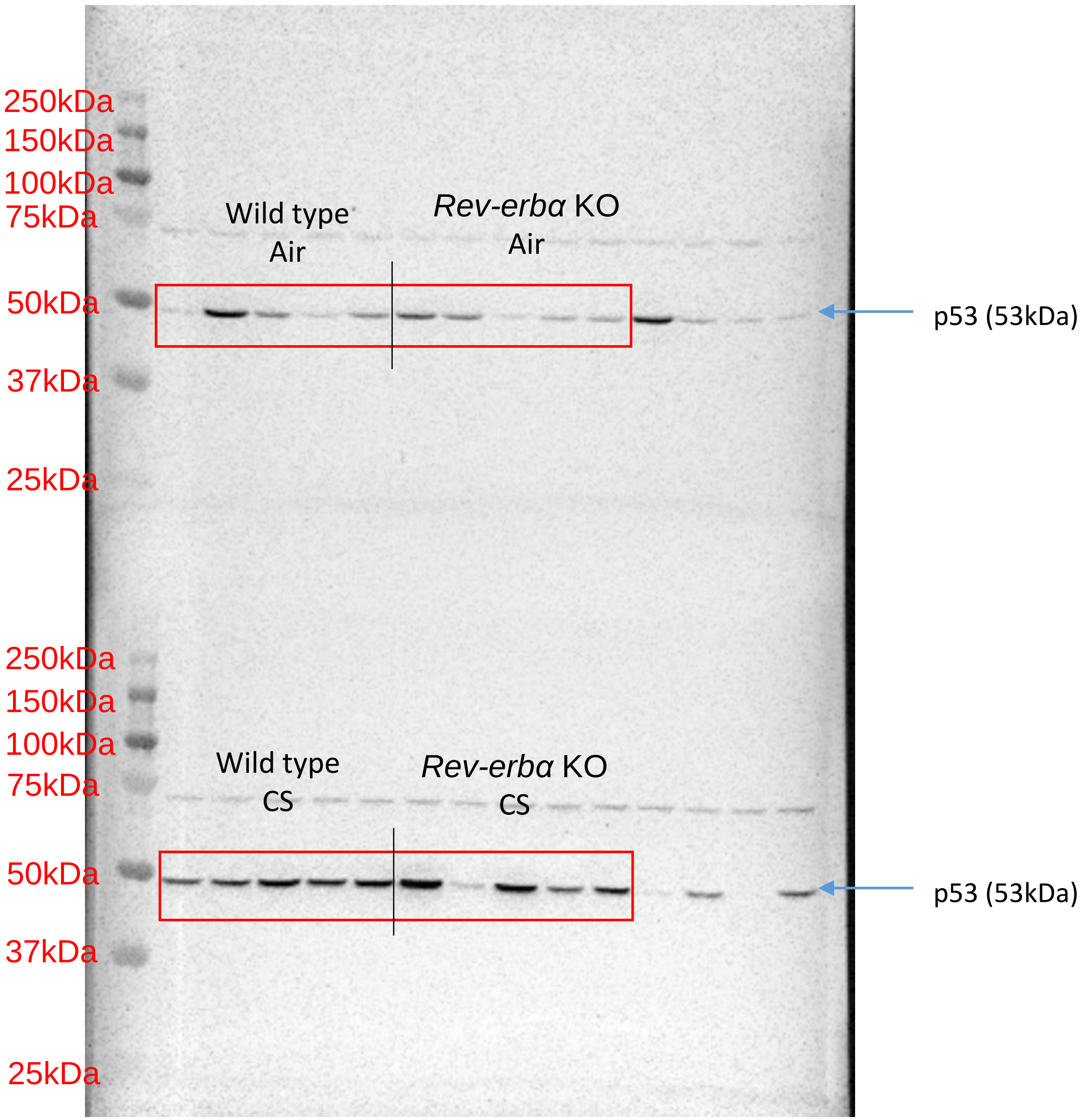

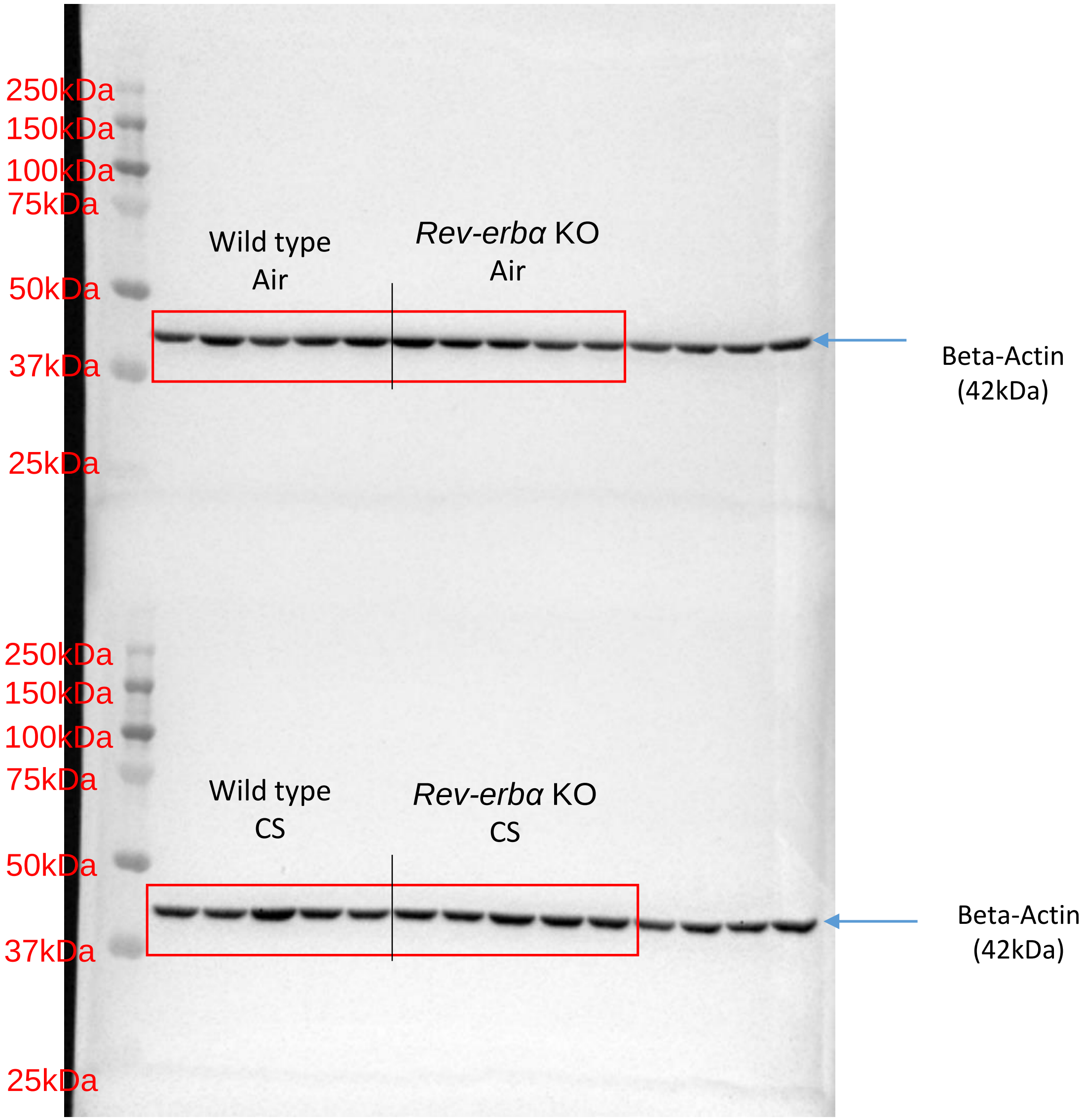

Full unedited gel for Fig 5: PAI-1 and β-Actin

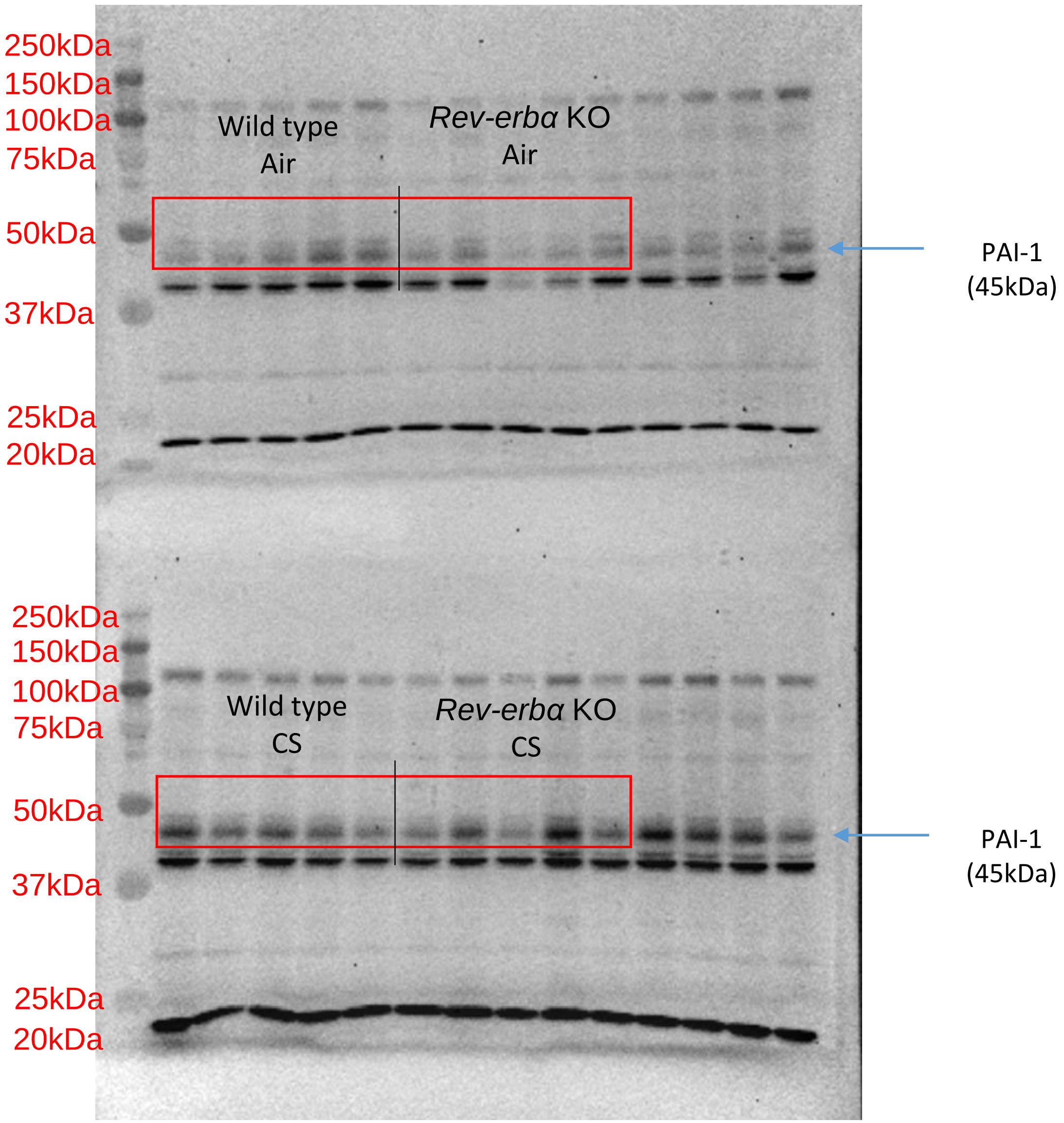

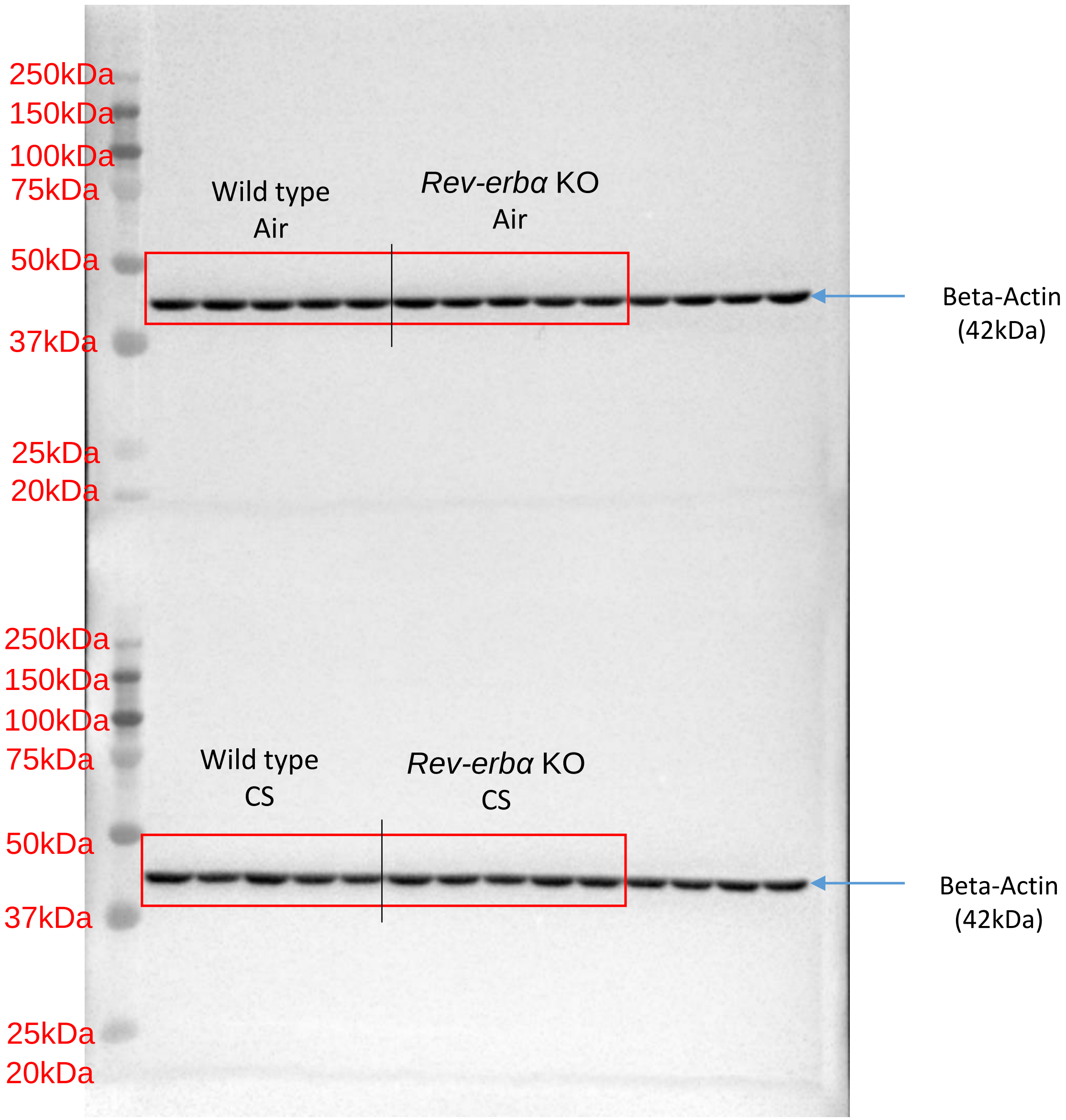

Full unedited gel for Fig 9A: COL1A1, TGFβ and β-Actin

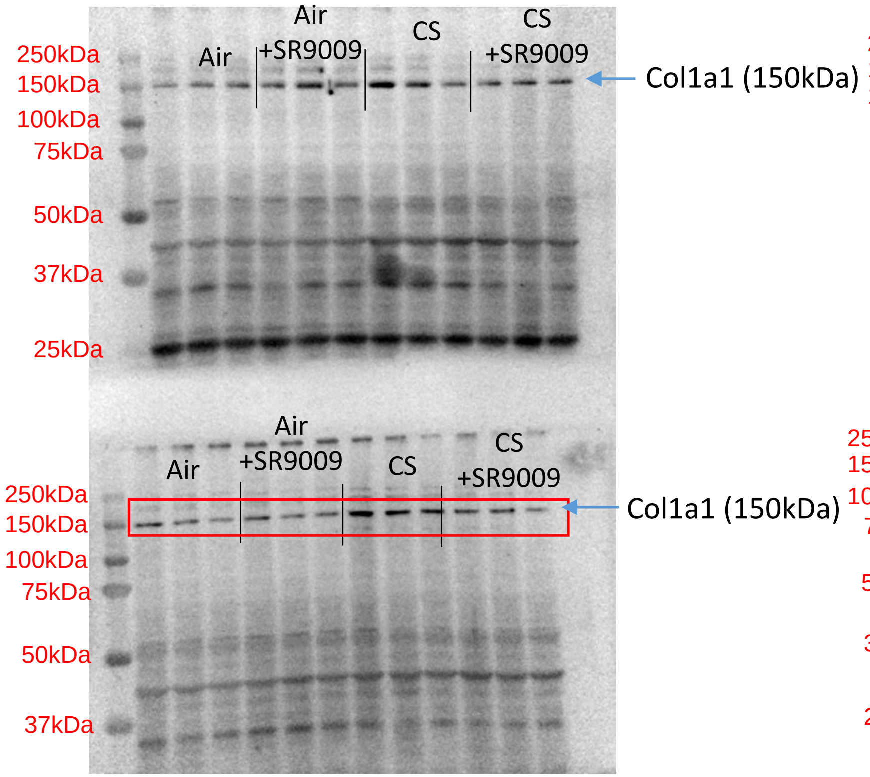

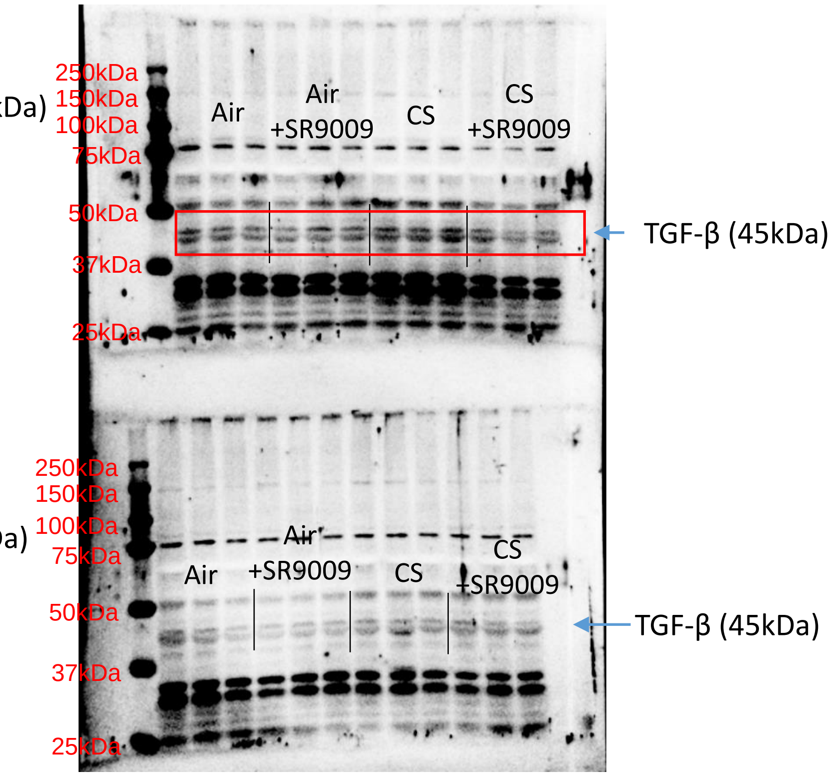

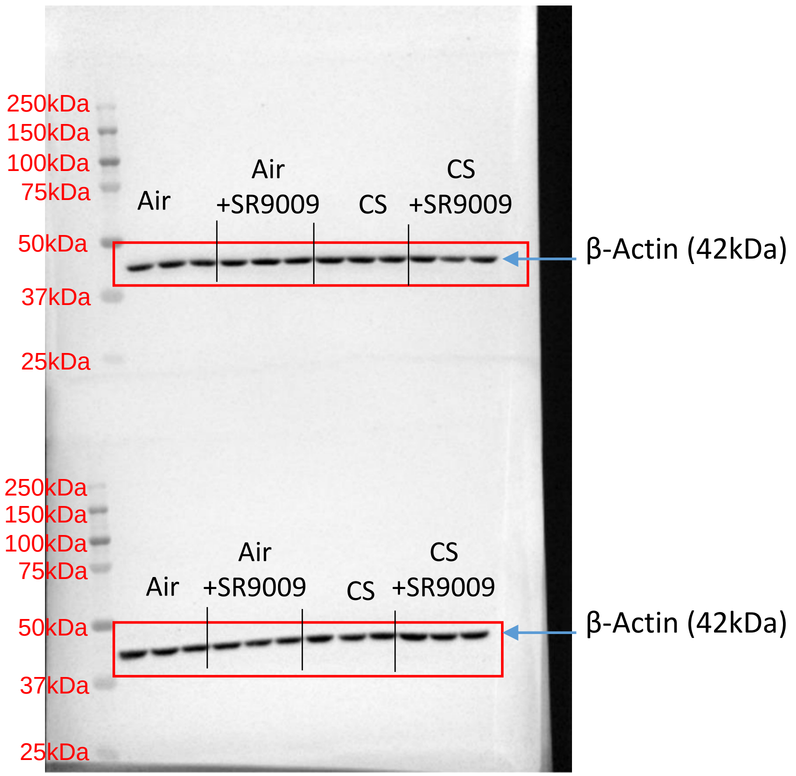

Only bands in red square represented in final figures, others were used in data analysis

*Col1A1 and TGFβ were probed on one membrane

Full unedited gel for Fig9A: Vimentin and β-Actin

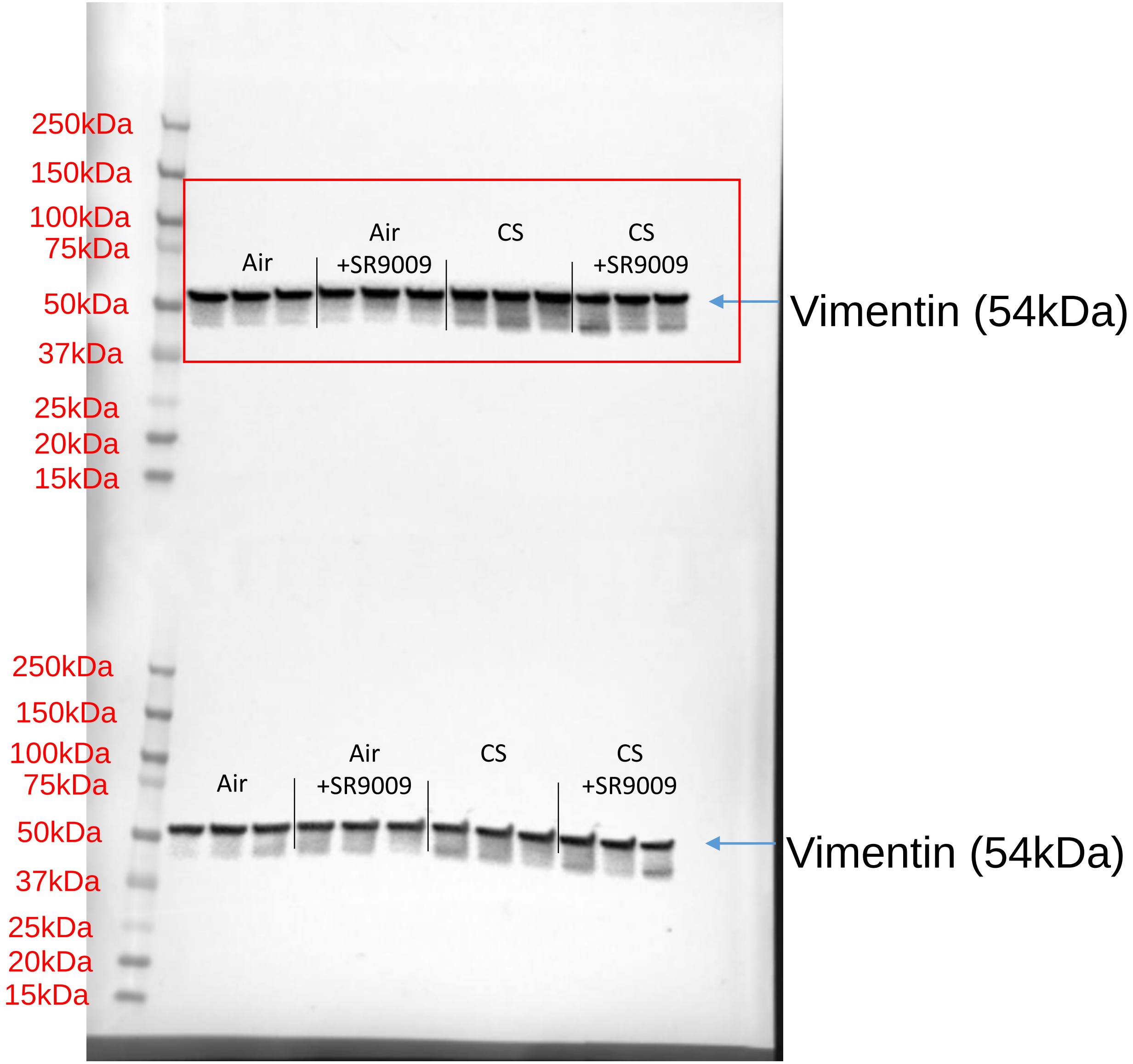

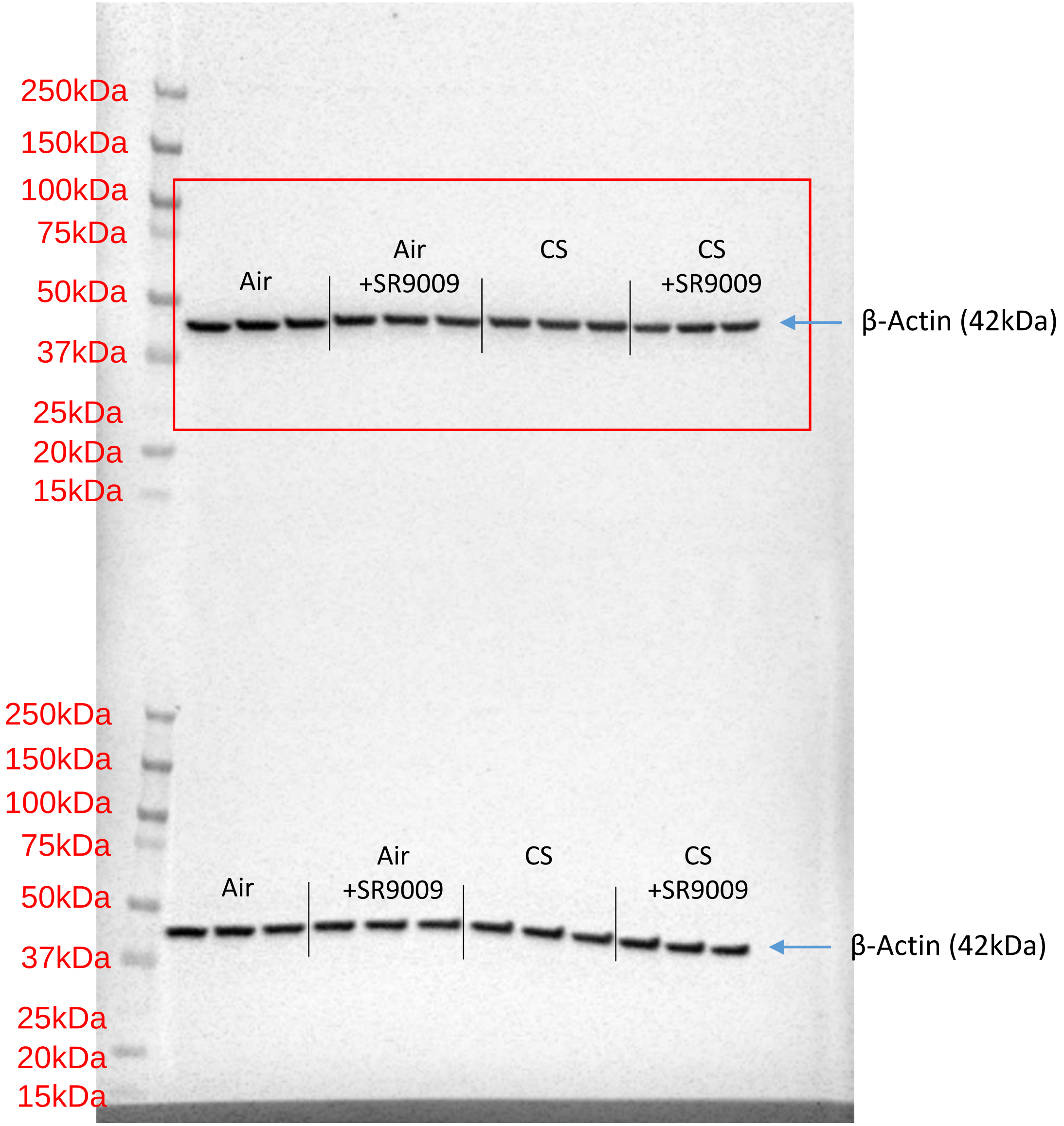

Only bands in red square represented in final figures, others were used in data analysis

Full unedited gel for Fig 9A: Snail/Slug and β-Actin

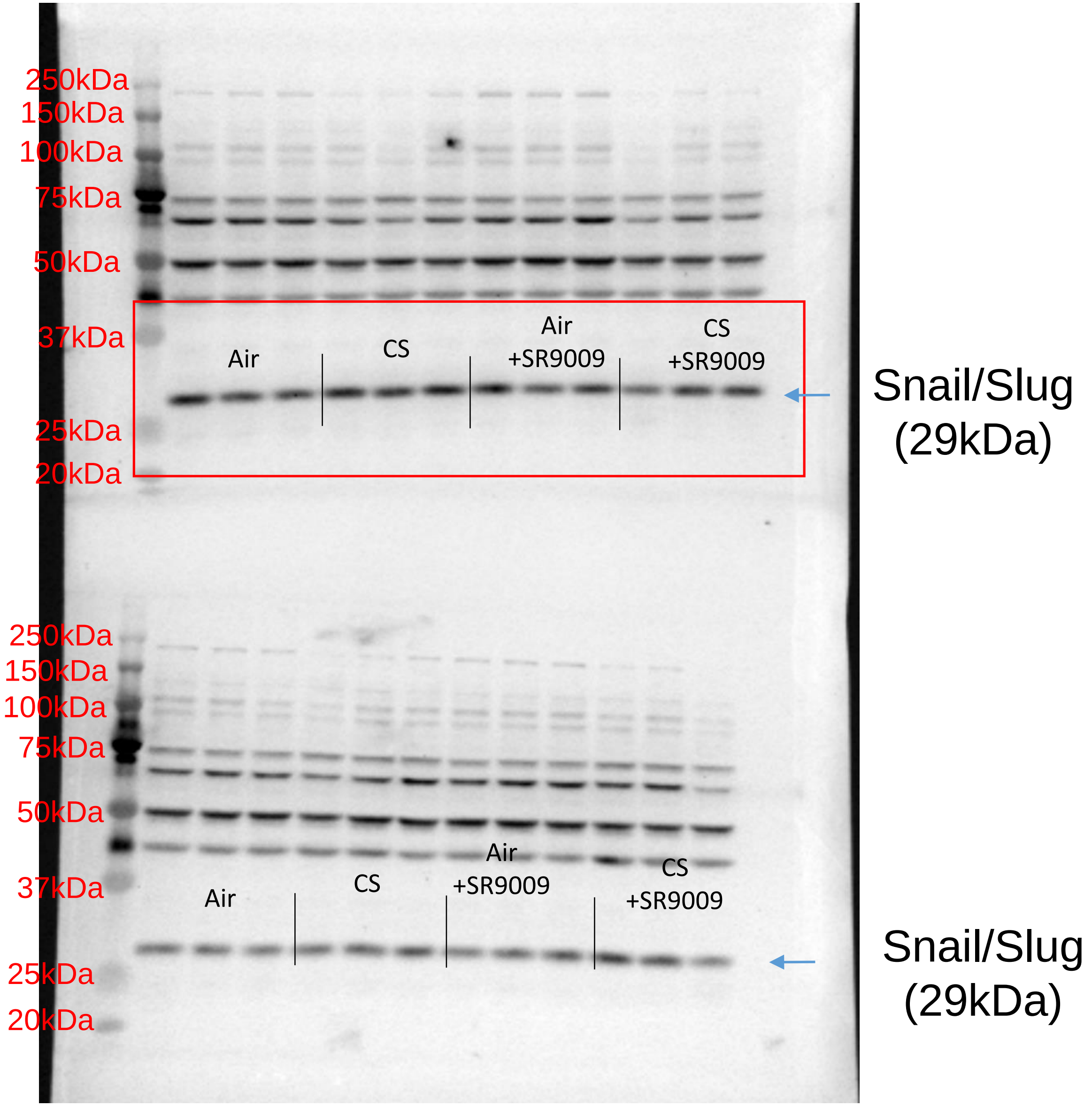

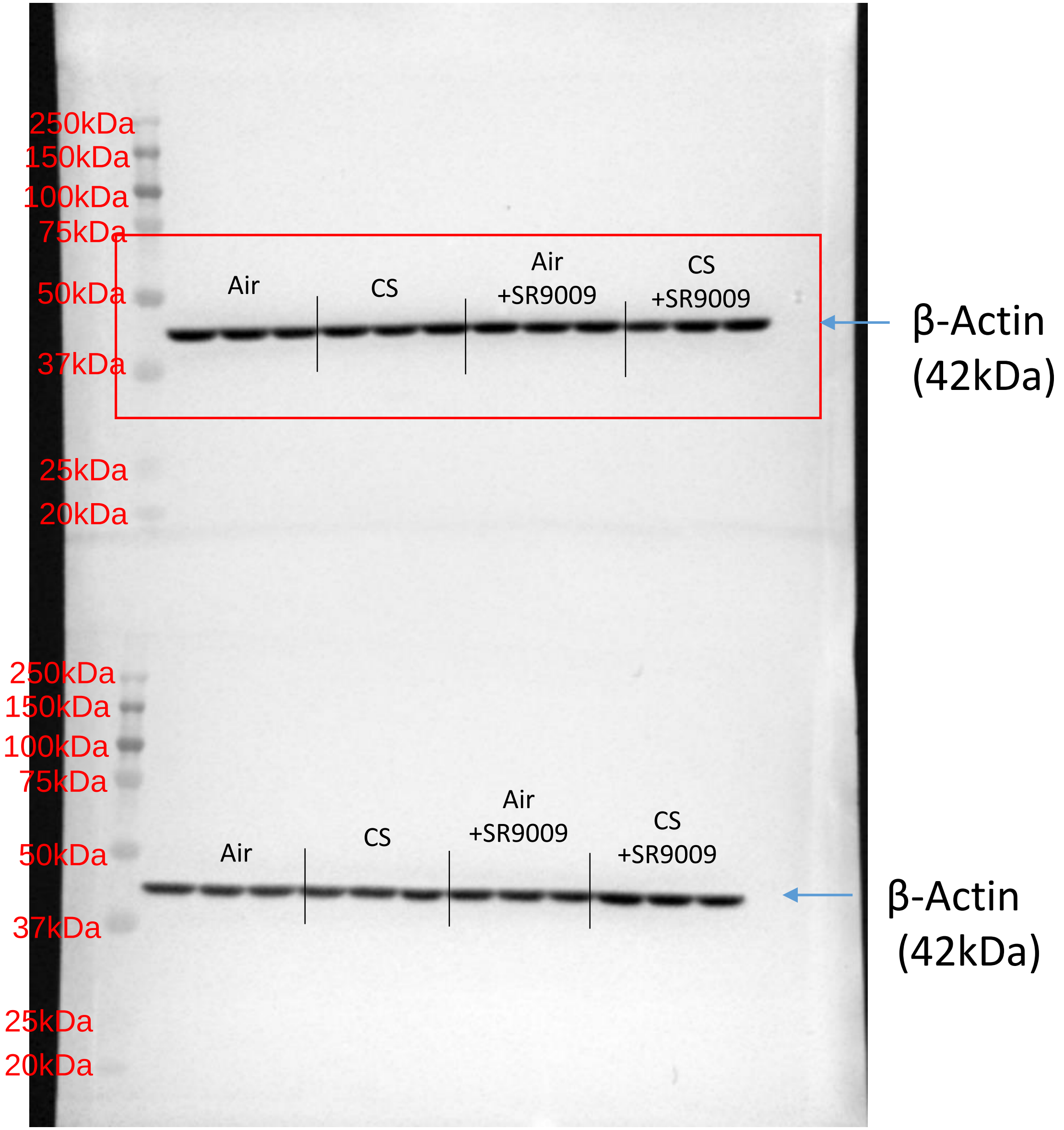

Only bands in red square represented in final figures, others were used in data analysis

Full unedited gel for Fig10: E-cadherin, ZO-1 and β-Actin

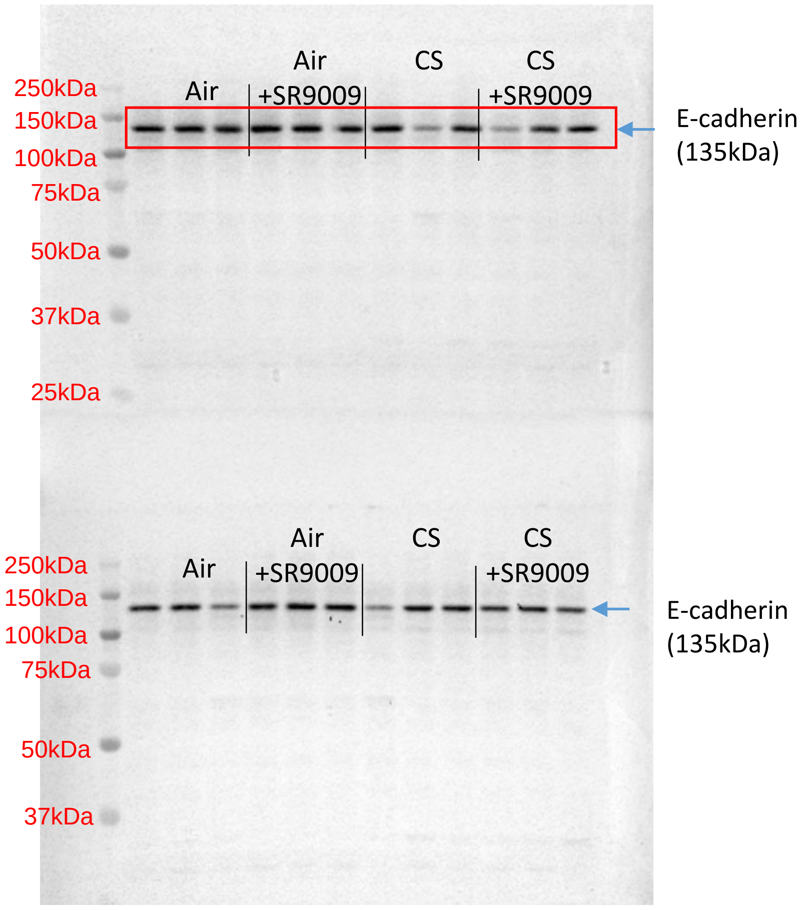

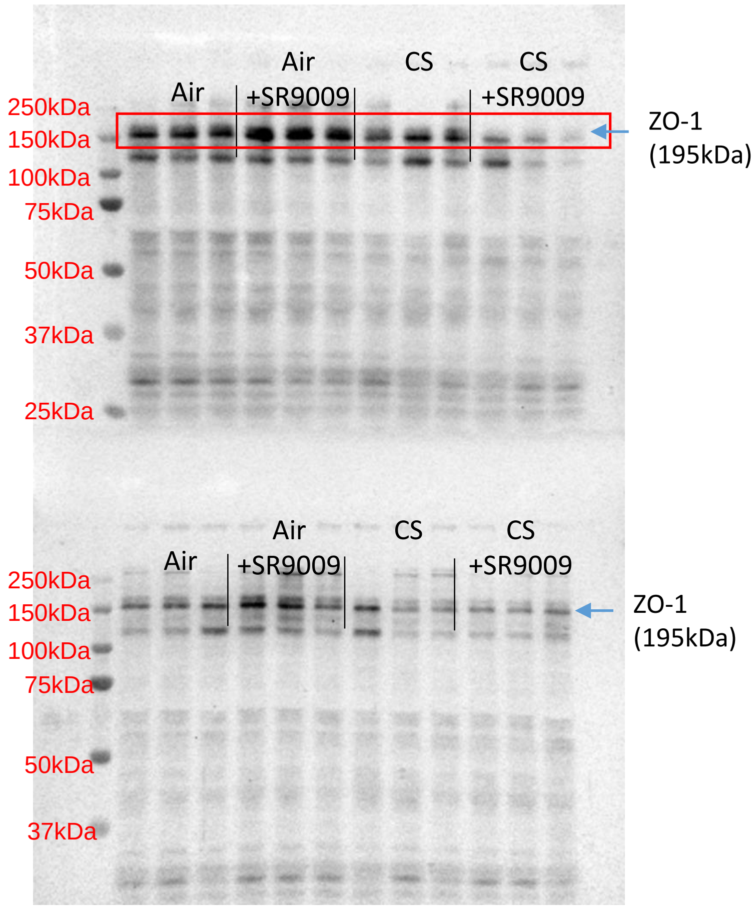

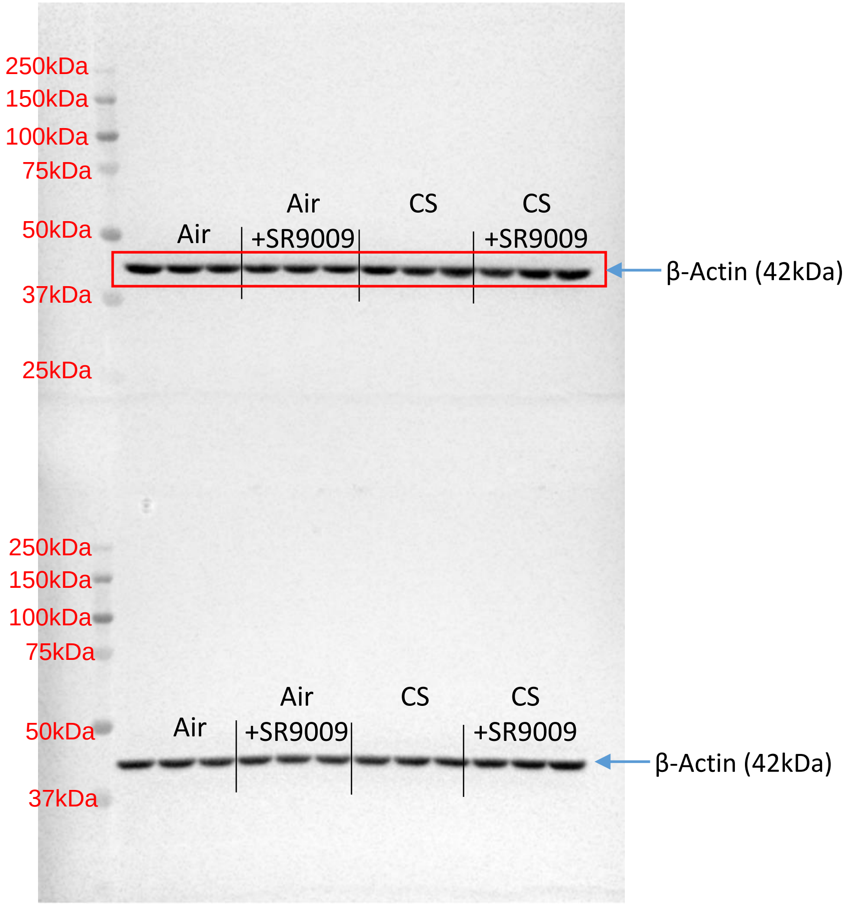

*presented bands for E-cadherin and ZO-1 were selected in one membrane, so both of the targets were shared with the same beta-actin

Only bands in red square represented in final figures, others were used in data analysis

Full unedited gel for Fig 10: p53 and β-Actin

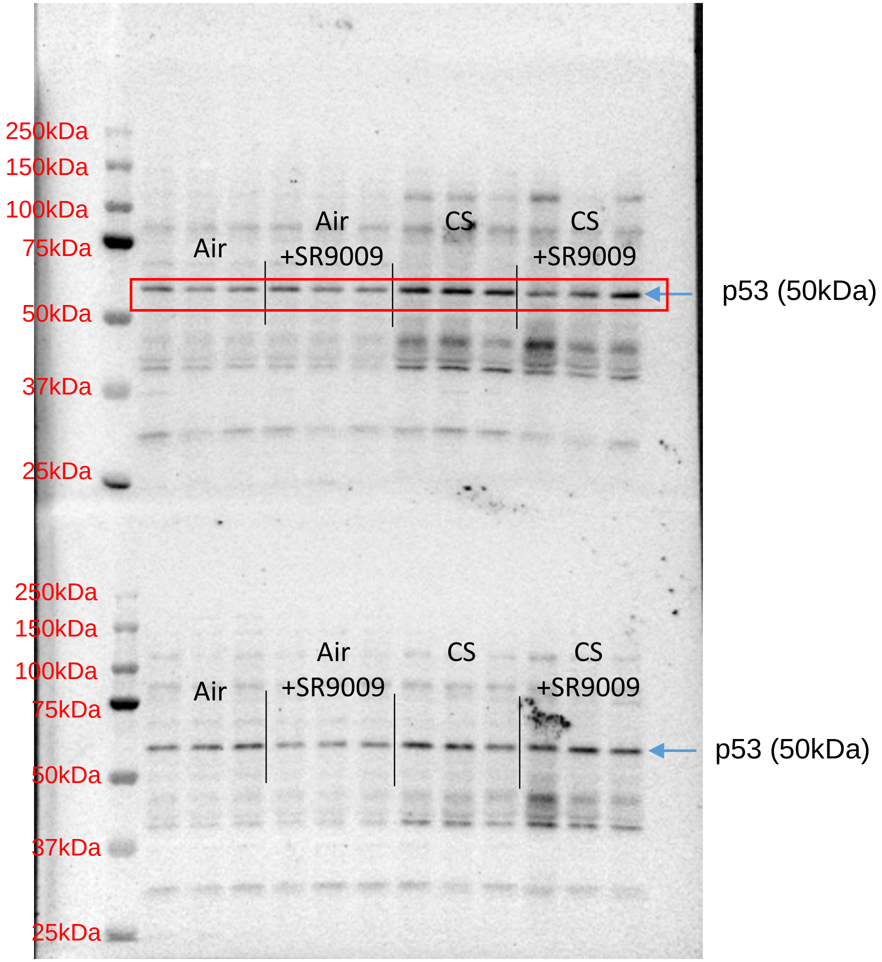

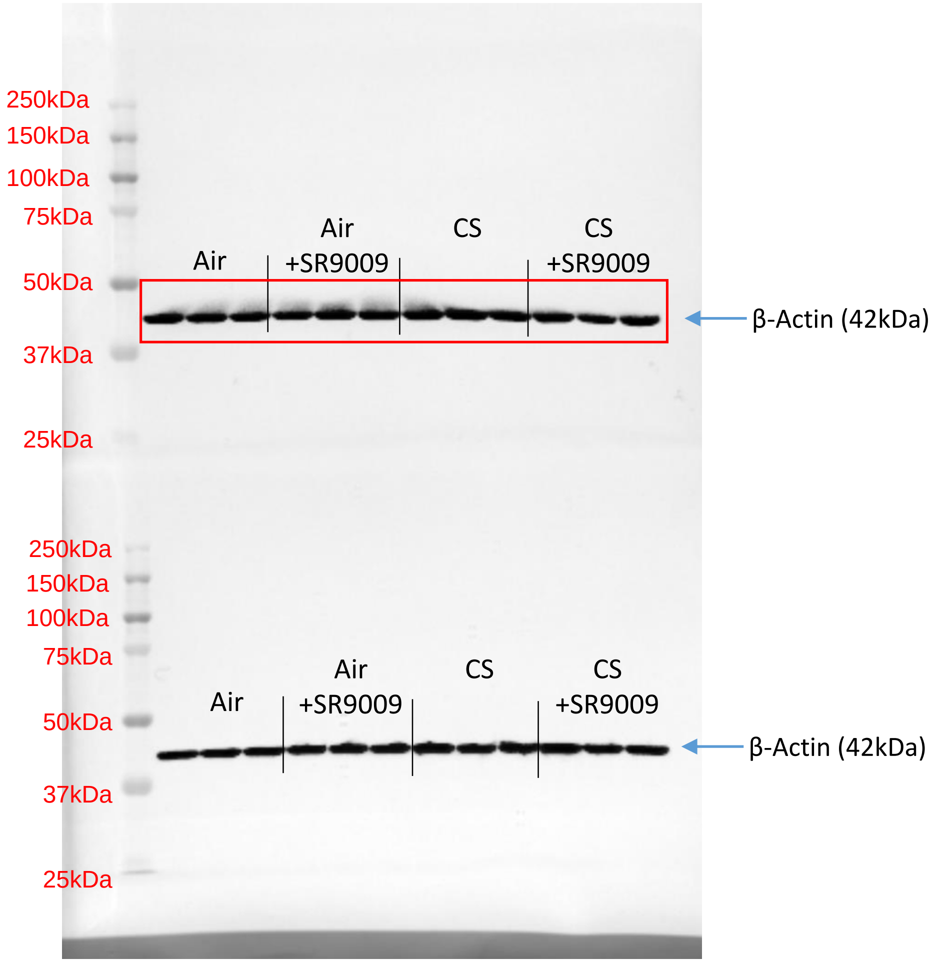

Only bands in red square represented in final figures, others were used in data analysis

Full unedited gel for Fig 10: PAI-1 and β-Actin

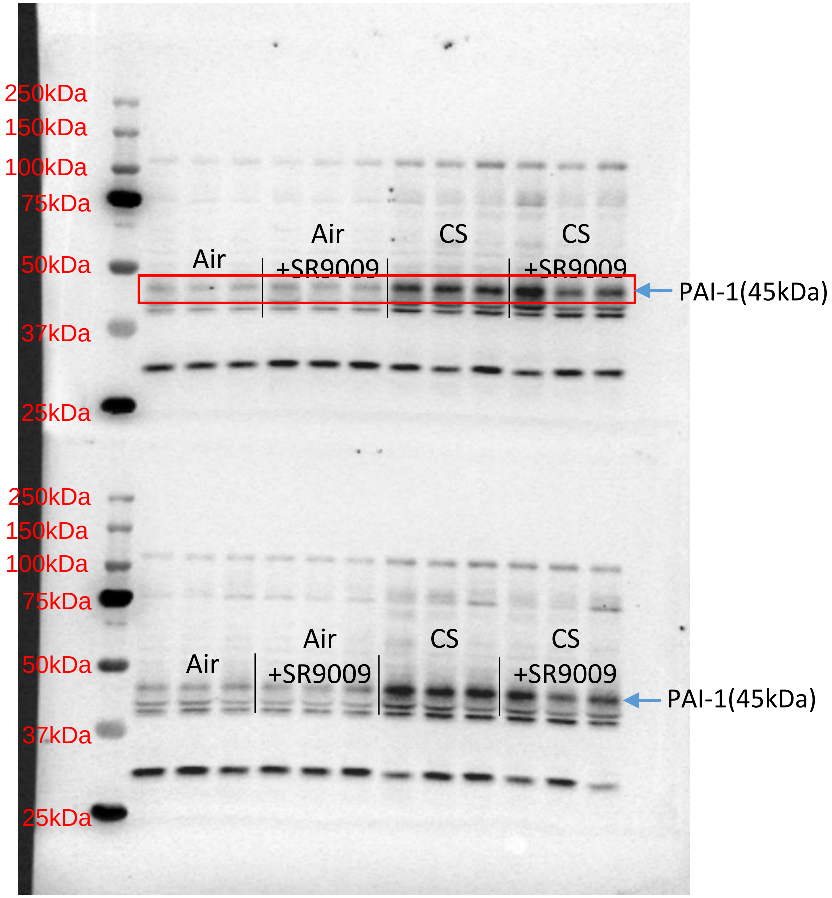

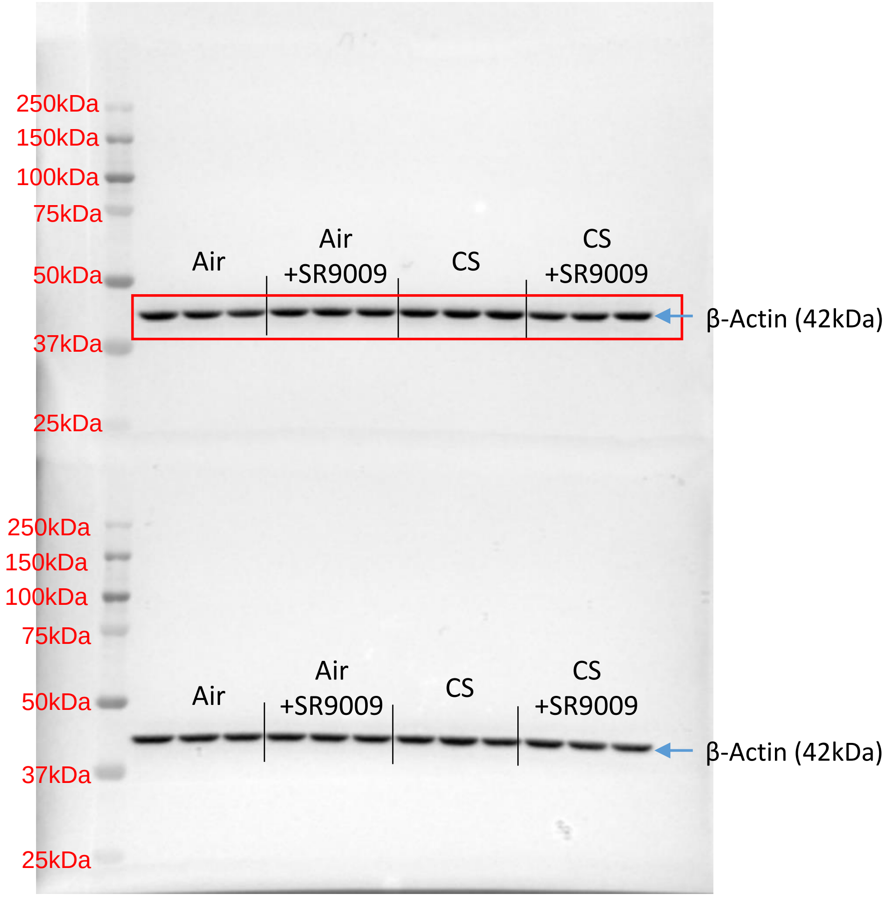

Only bands in red square represented in final figures, others were used in data analysis

Full unedited gel for Fig S2: ROR α, BMAL-1 and β-Actin

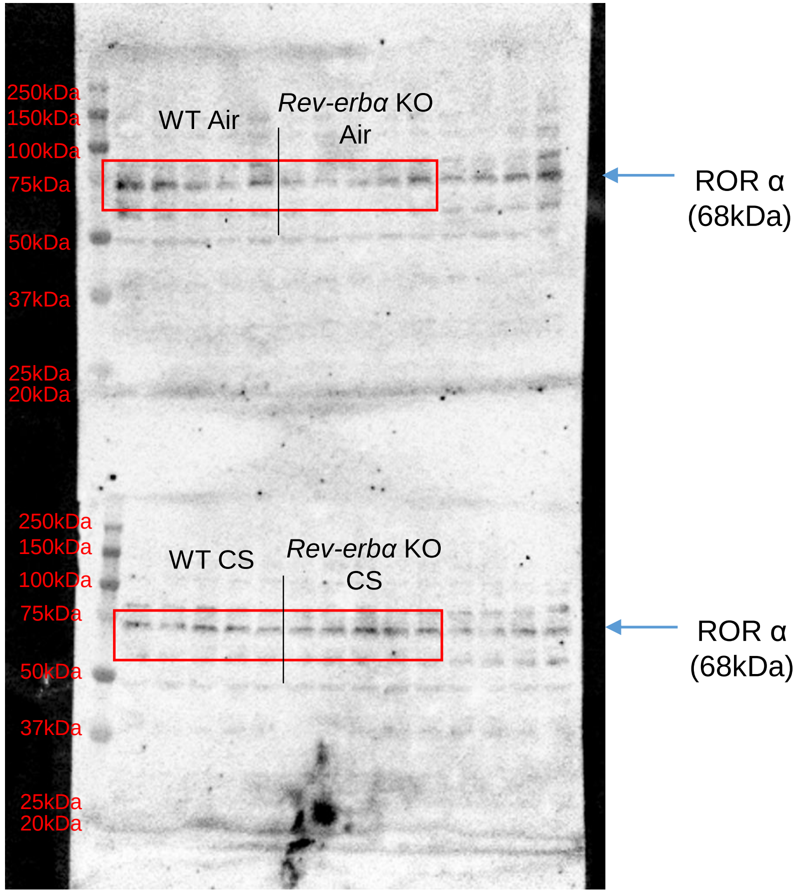

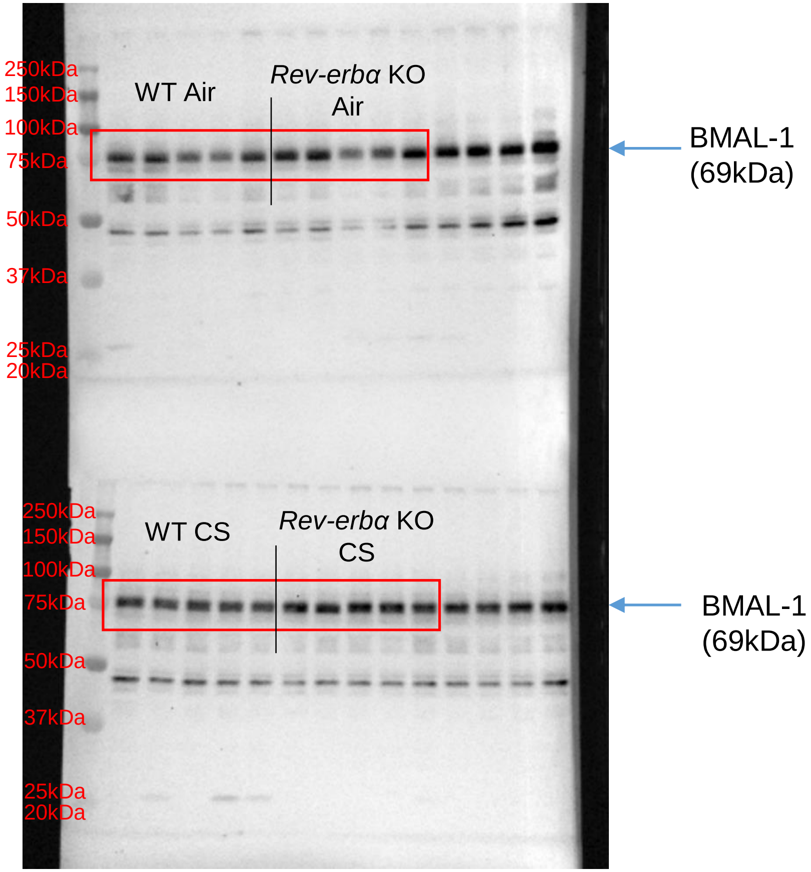

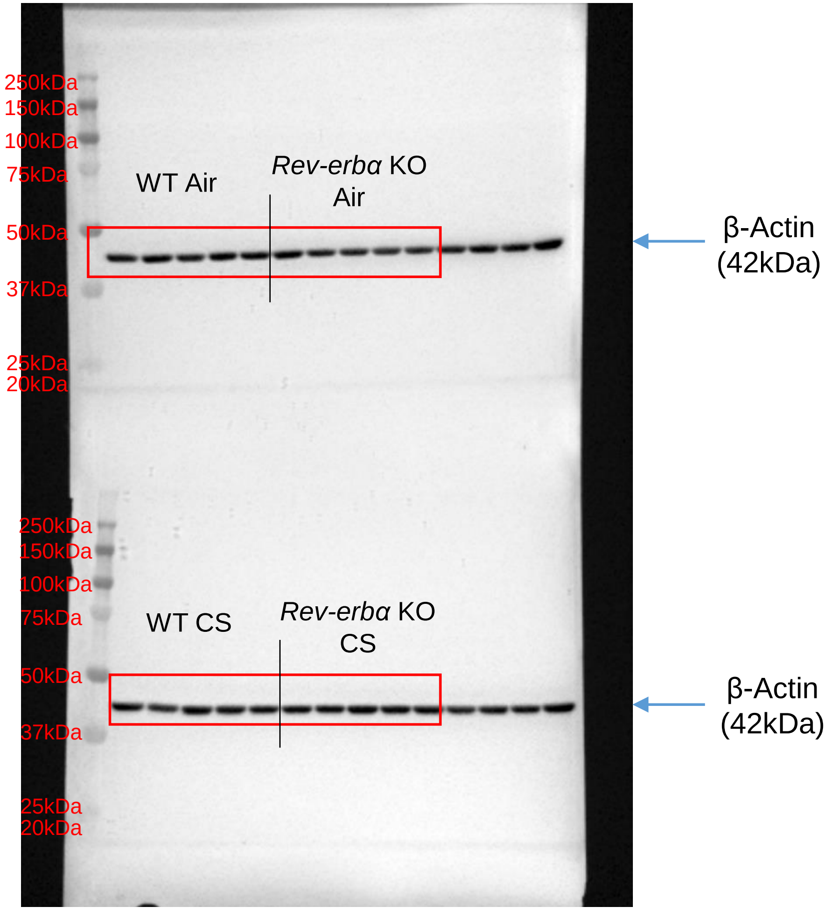

*RORα and BMAL1 were probed on one membrane

Full unedited gel for Fig S2: CLOCK and β-Actin

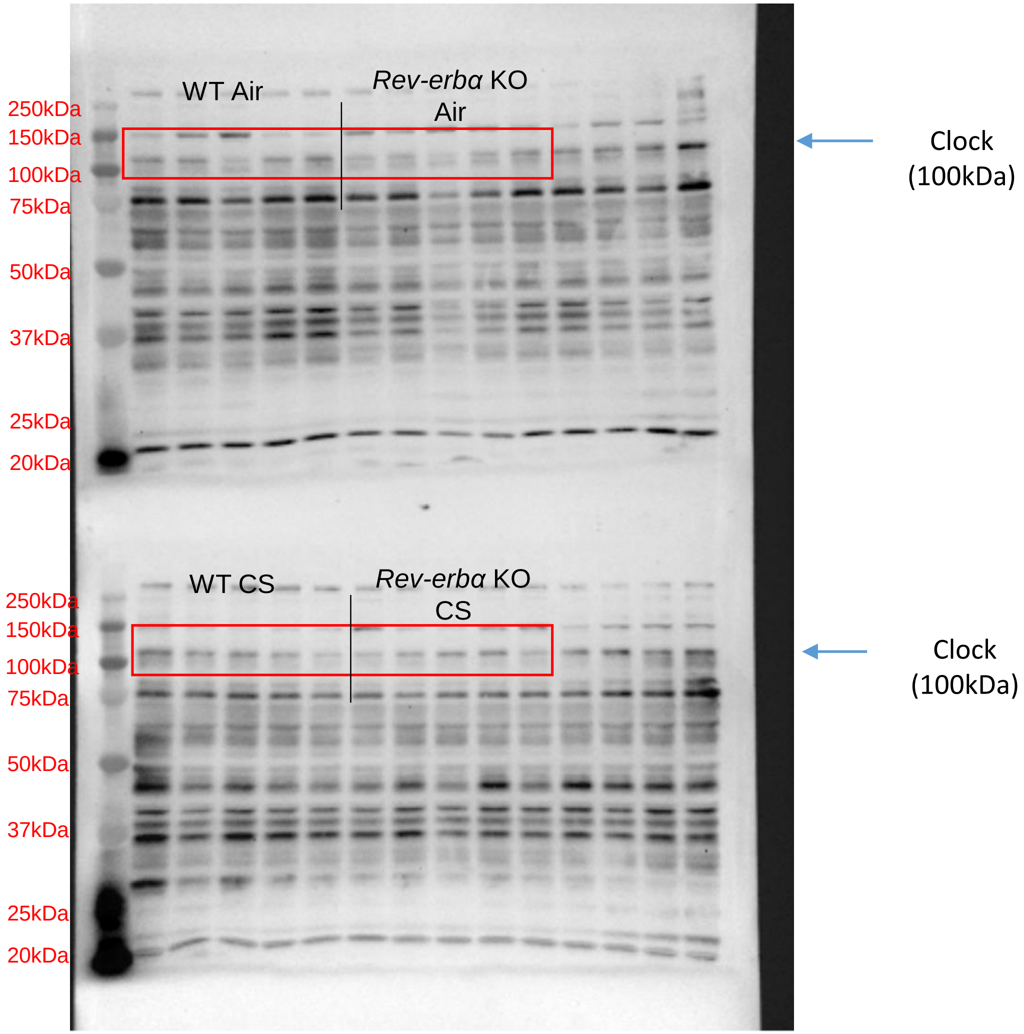

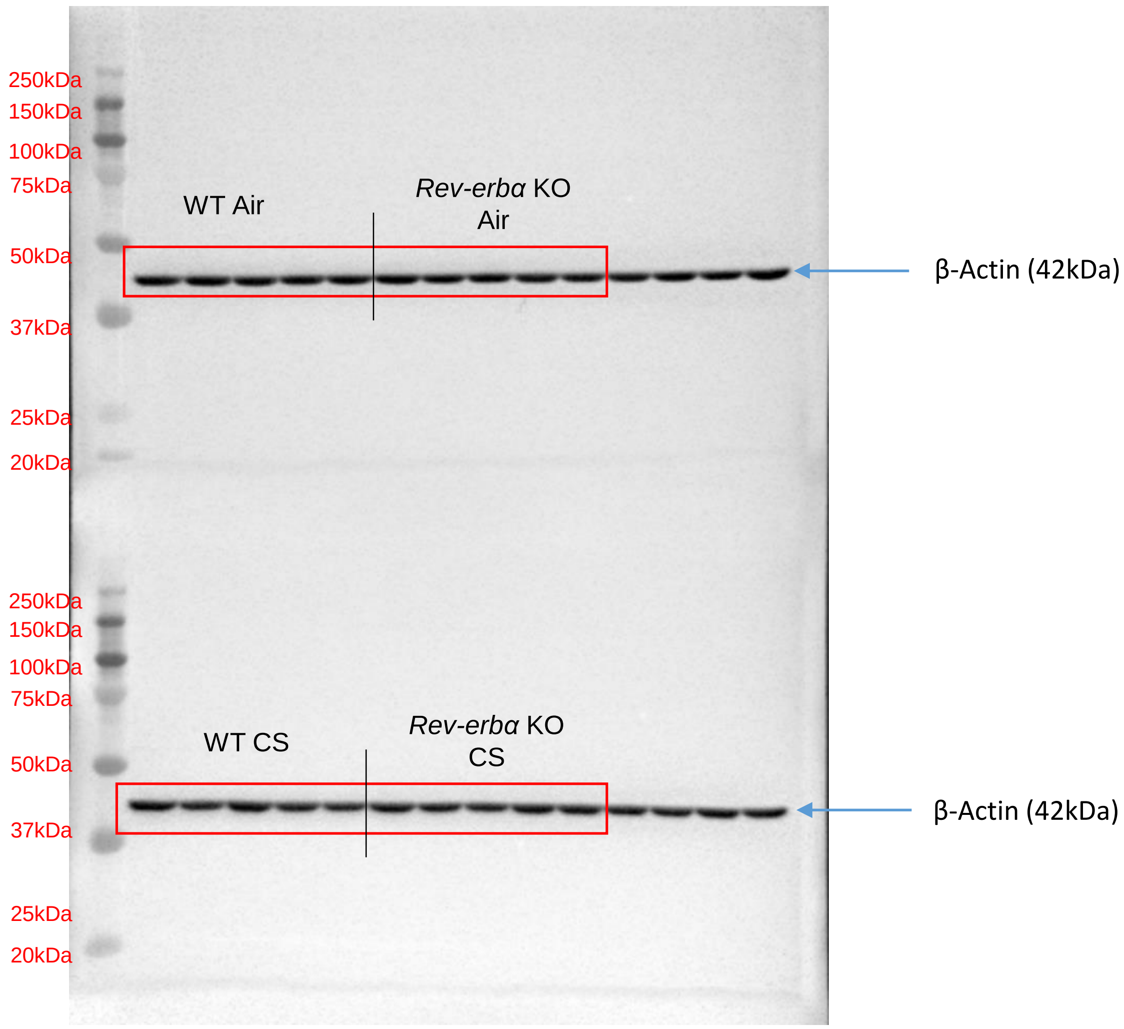

Full unedited gel for Fig S5: MMP2, MMP12 and β-Actin

Same beta actin were used for normalization of Snail-slug expression level as showed in Figure 4A

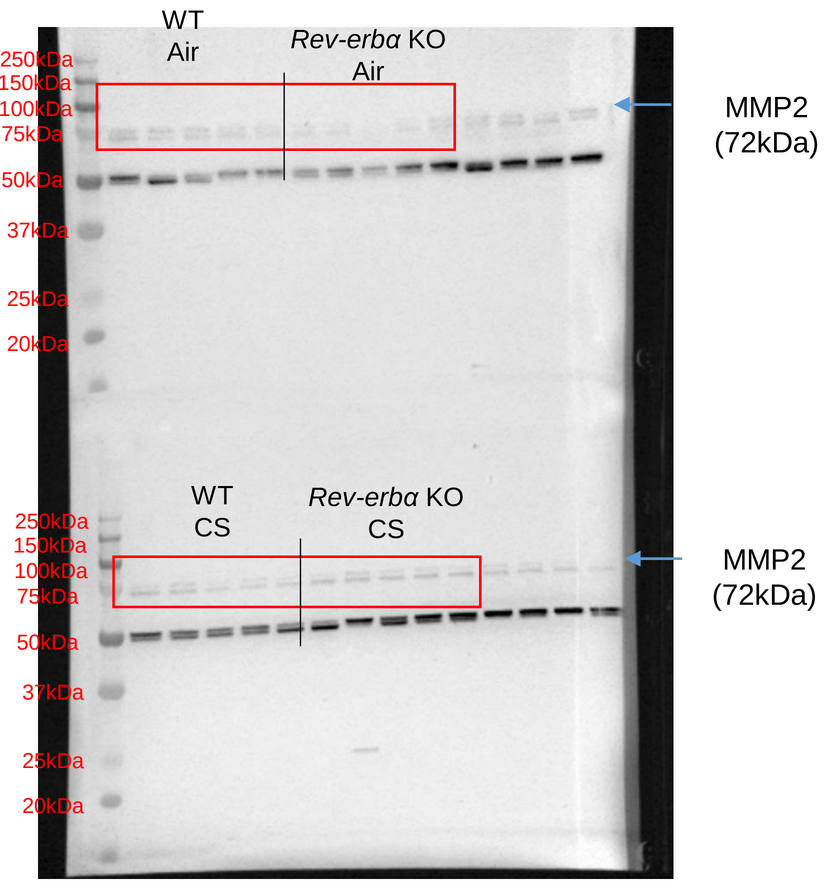

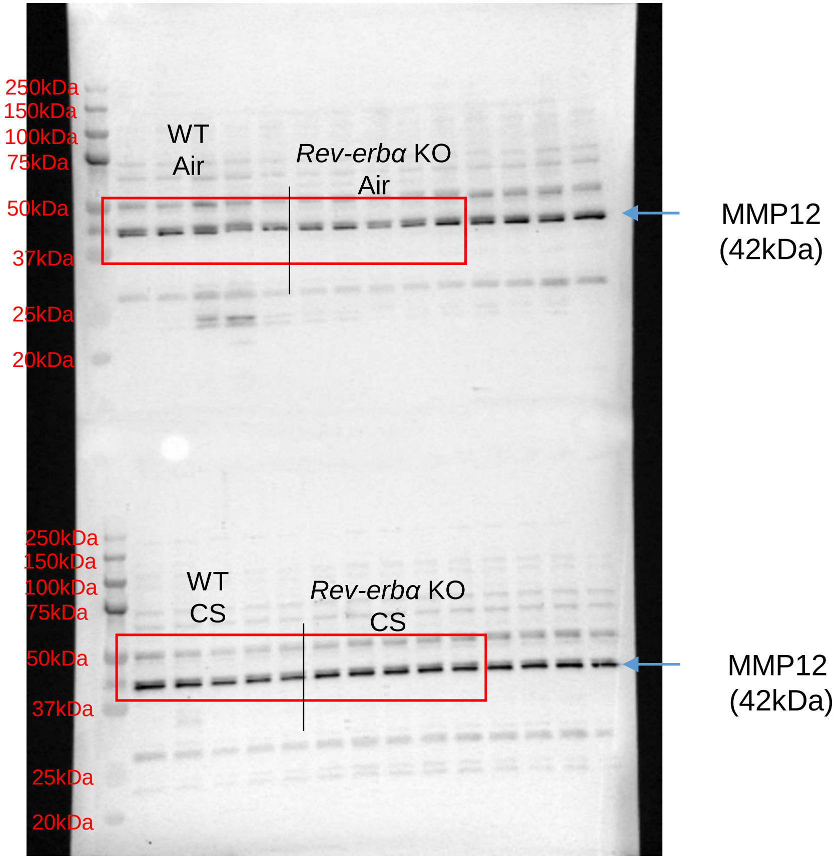

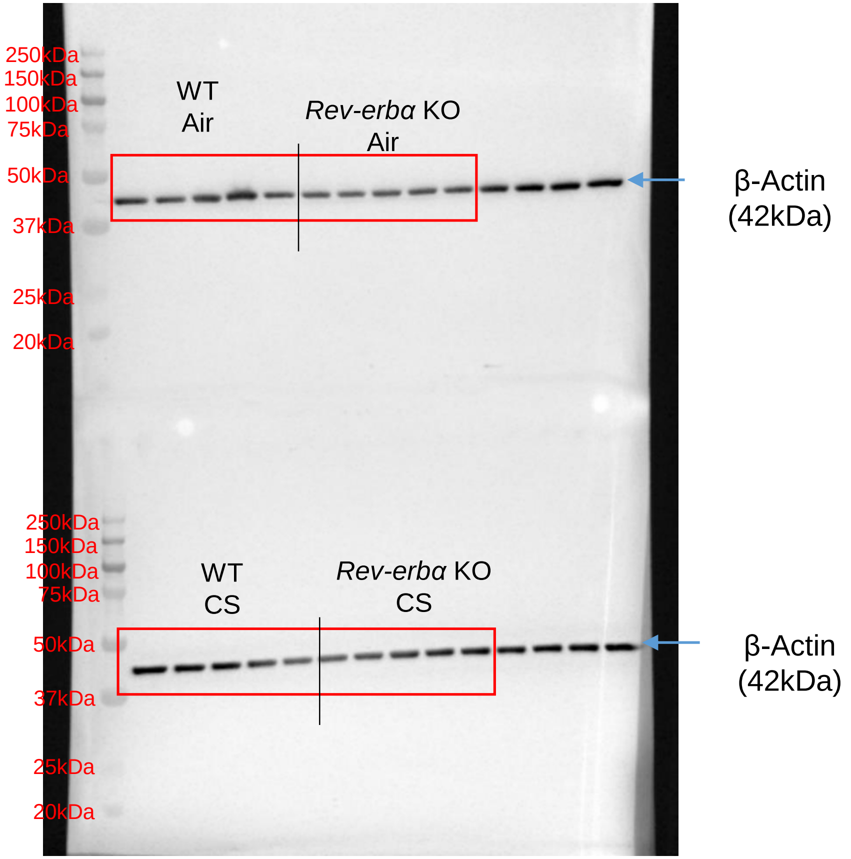

*MMP2 and MMP12 were probed on one membrane

Full unedited gel for Fig S5: MMP9 and β-Actin

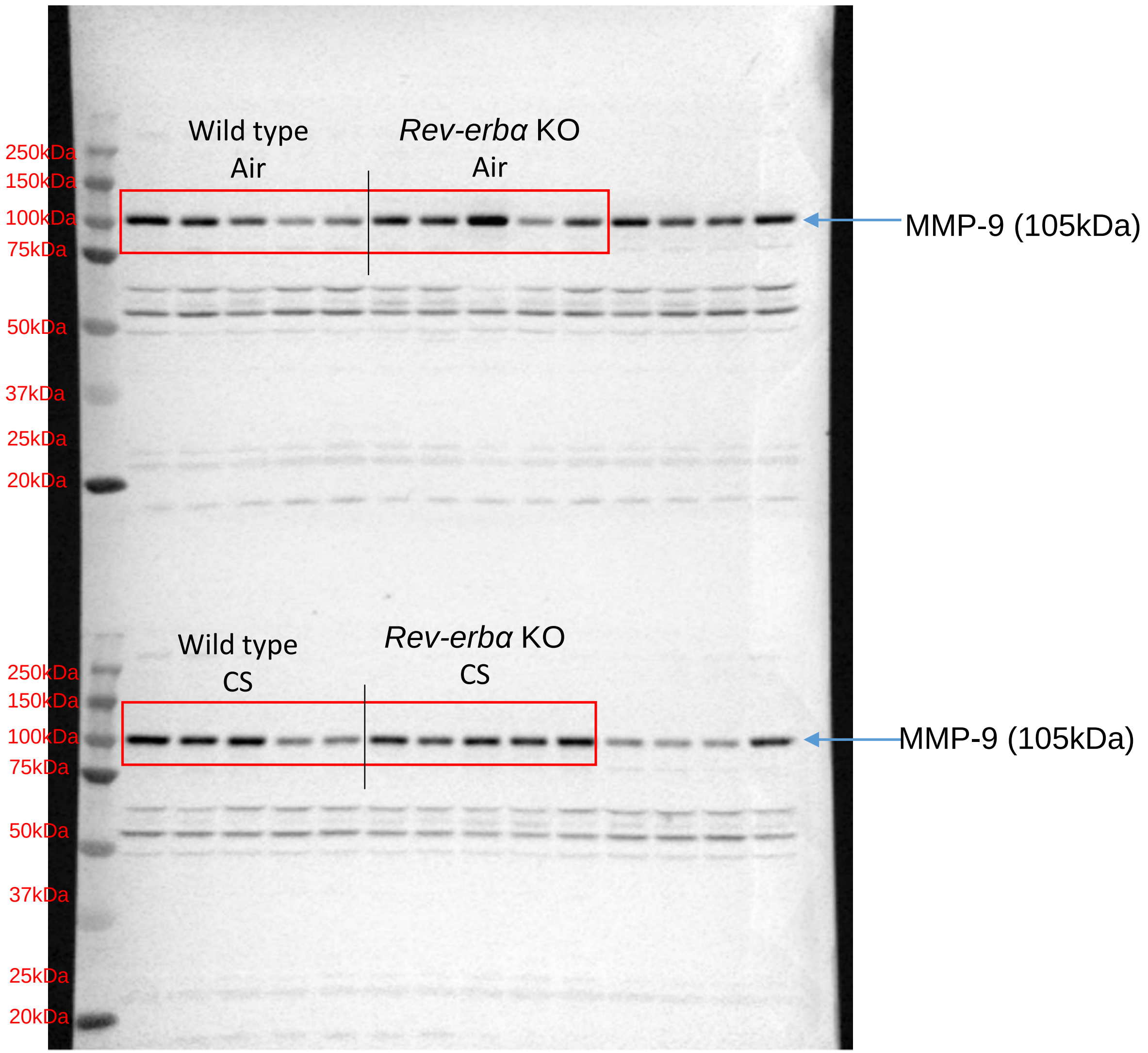

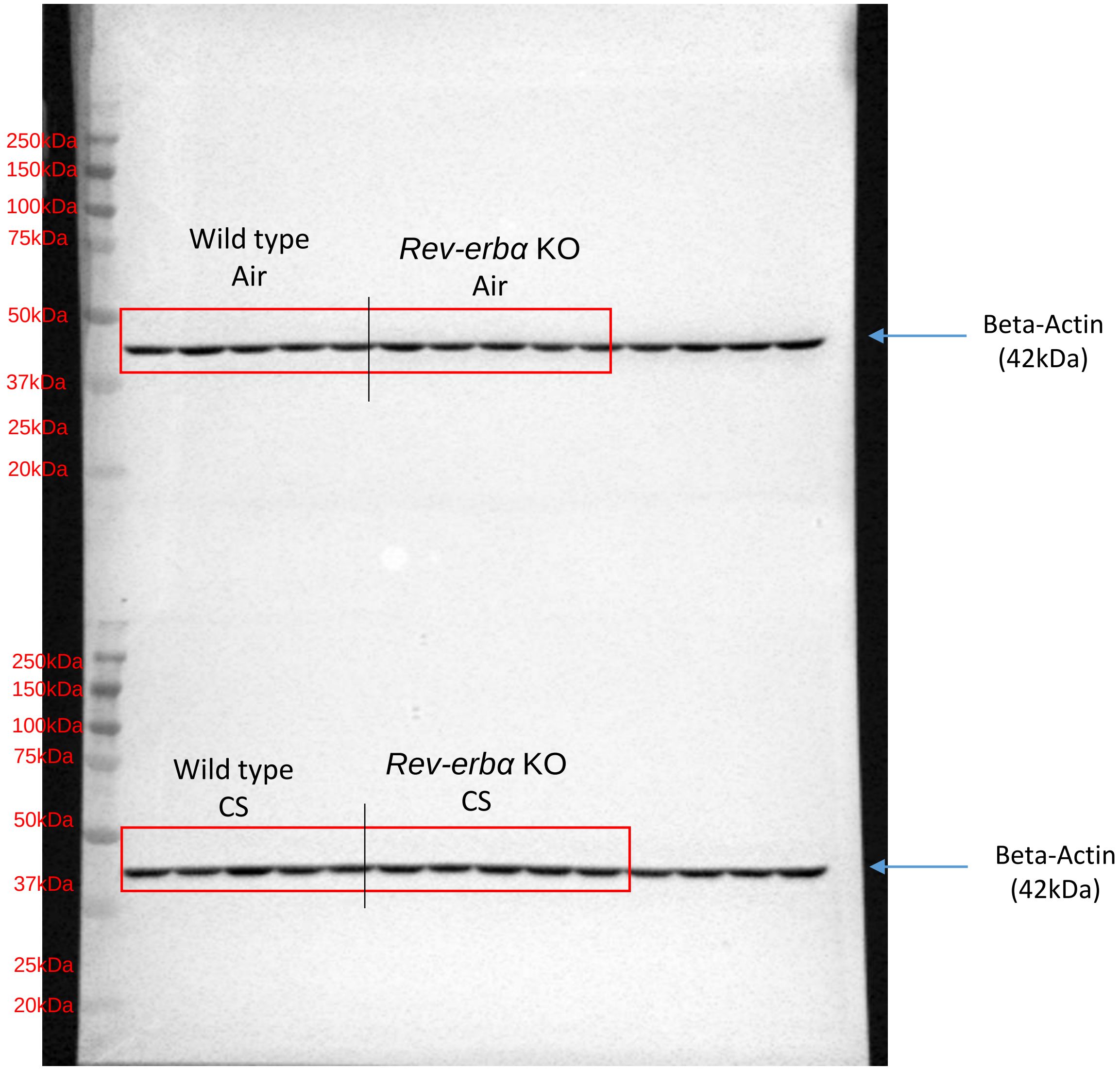

Full unedited gel for Fig S8: MMP2, MMP12 and β-Actin

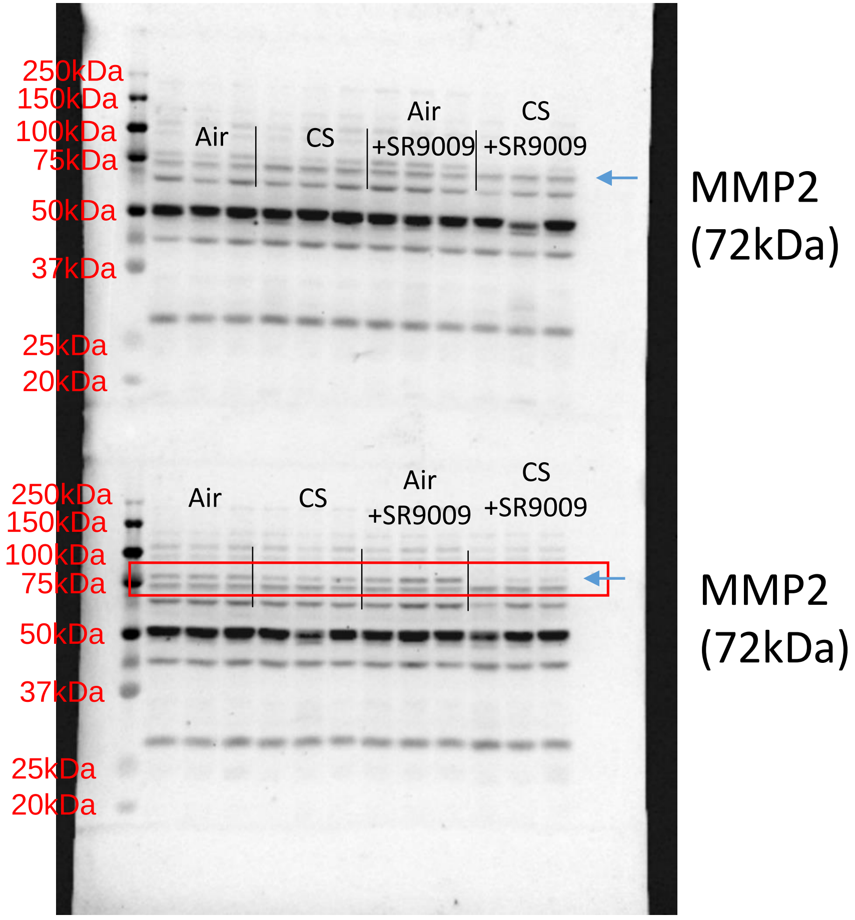

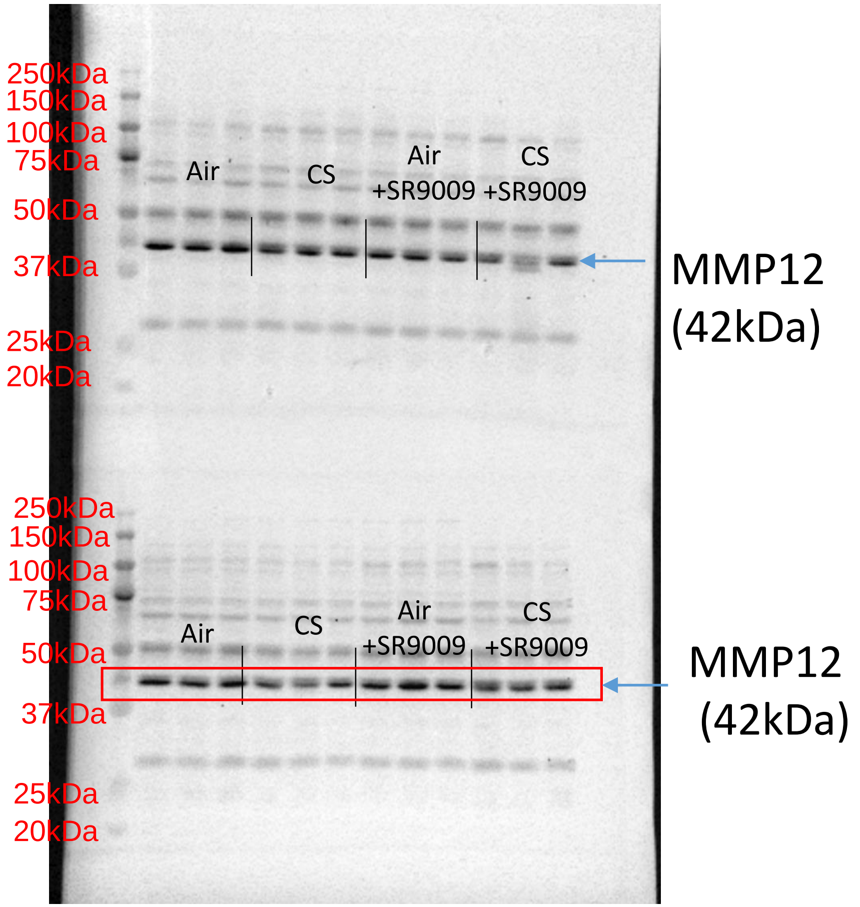

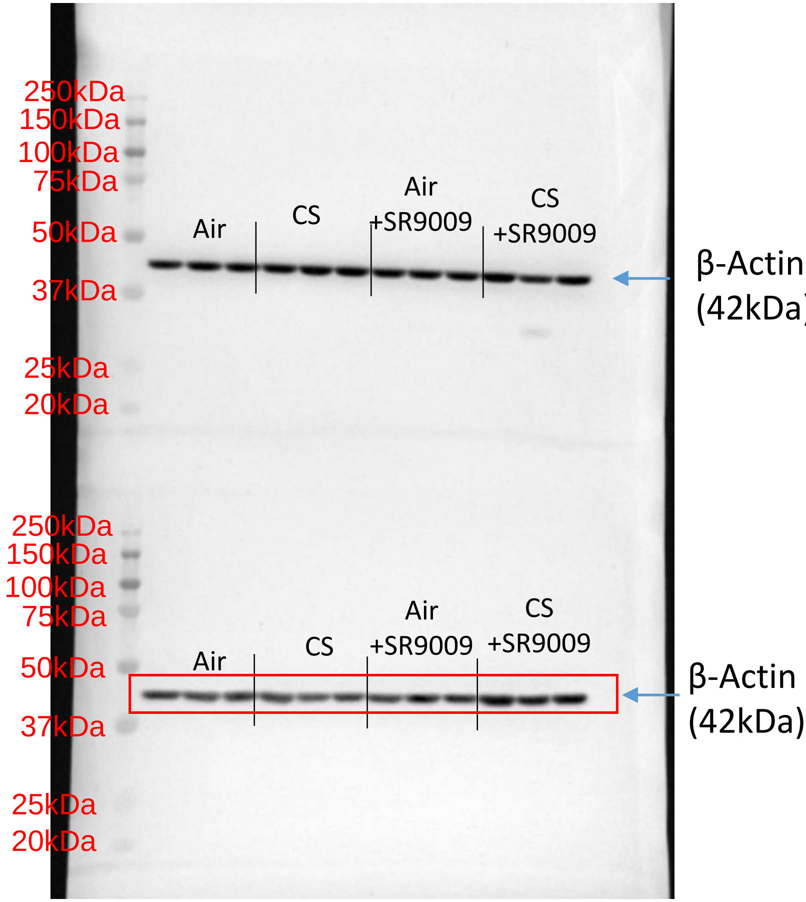

MMP2 and MMP12 were probed on one membrane

Only bands in red square represented in final figures, others were used in data analysis

Full unedited gel for Fig S8: MMP9 and β-Actin

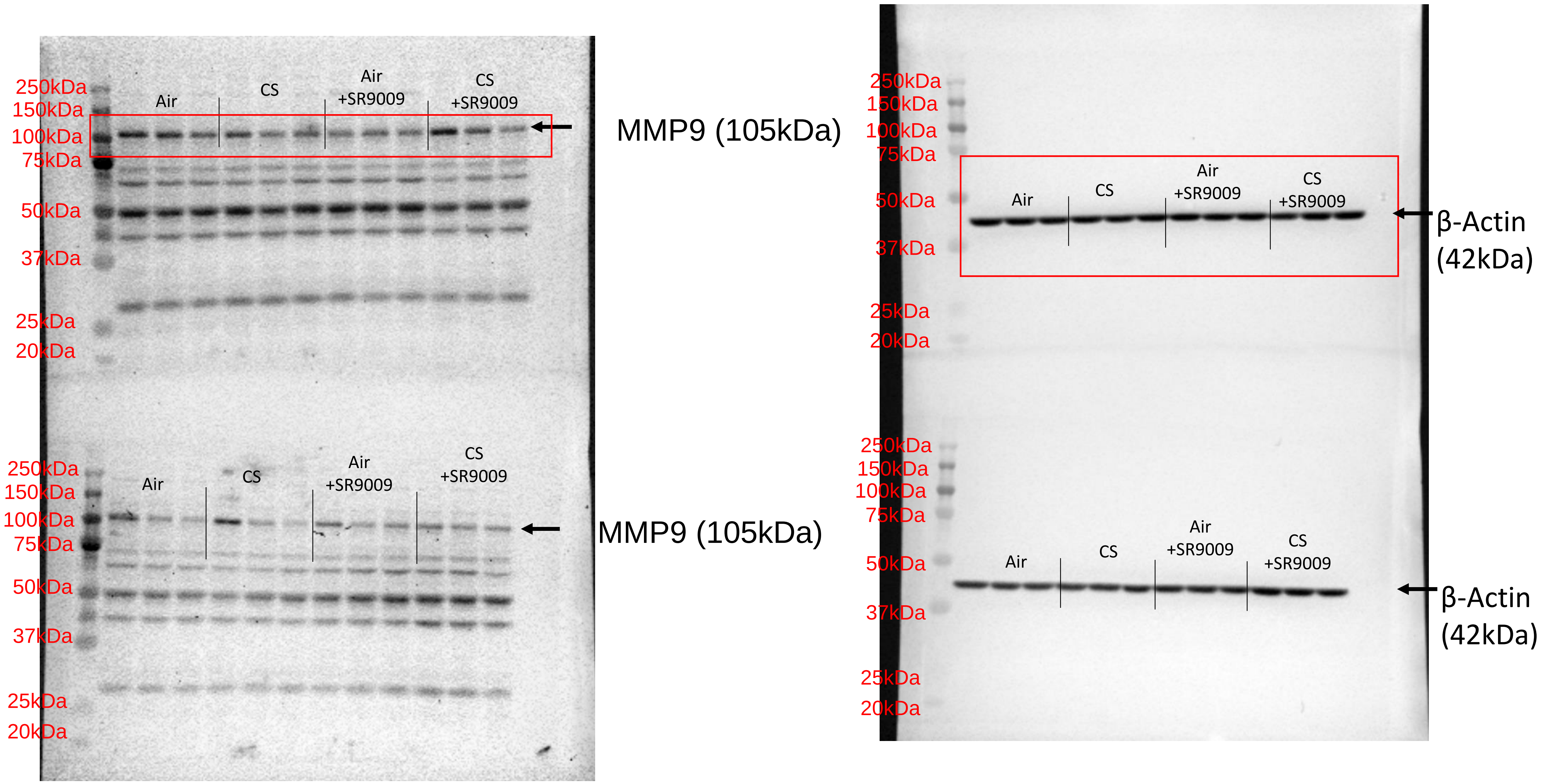

Only bands in red square represented in final figures, others were used in data analysis

Full unedited gel for Fig S9: BMAL1, CLOCK and β-Actin

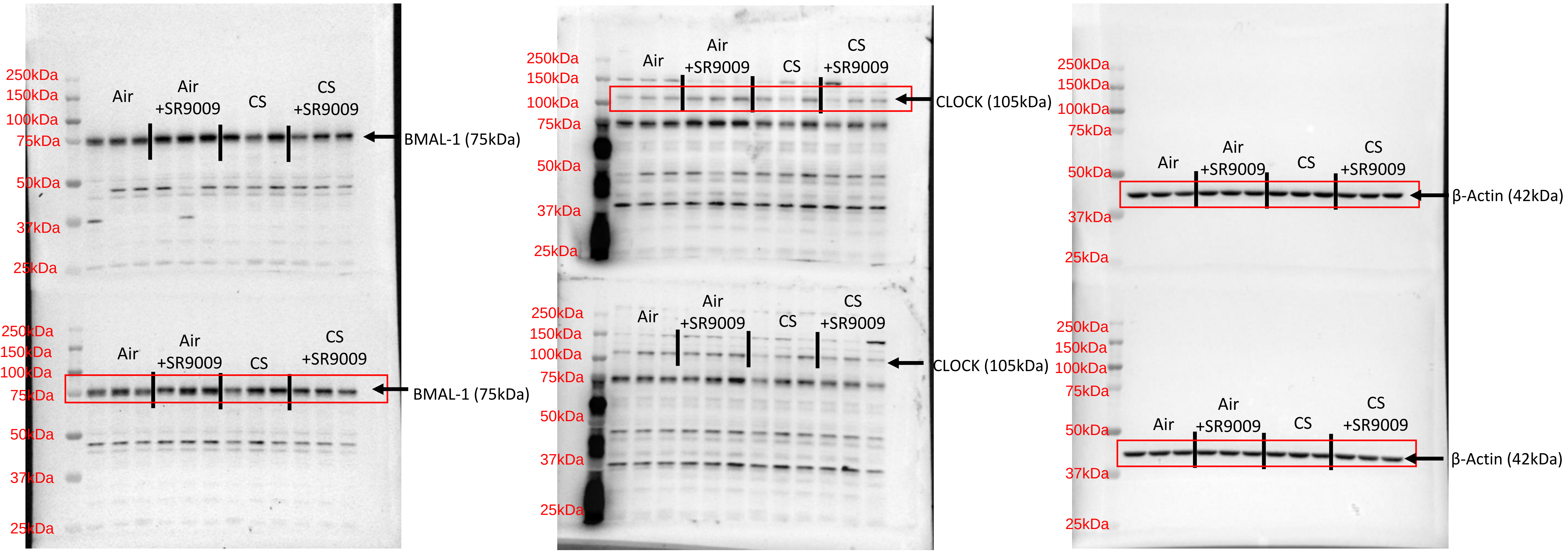

*BMAL-1 and CLOCK were probed on one membrane

Only bands in red square represented in final figures, others were used in data analysis

Full unedited gel for Fig S9: RORα and β-Actin

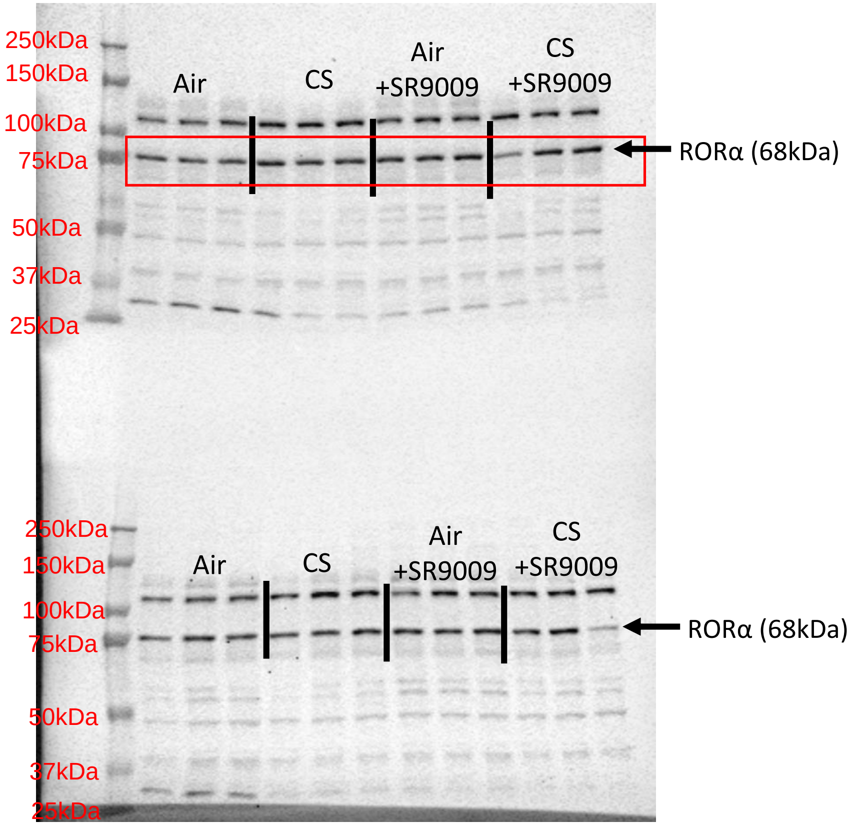

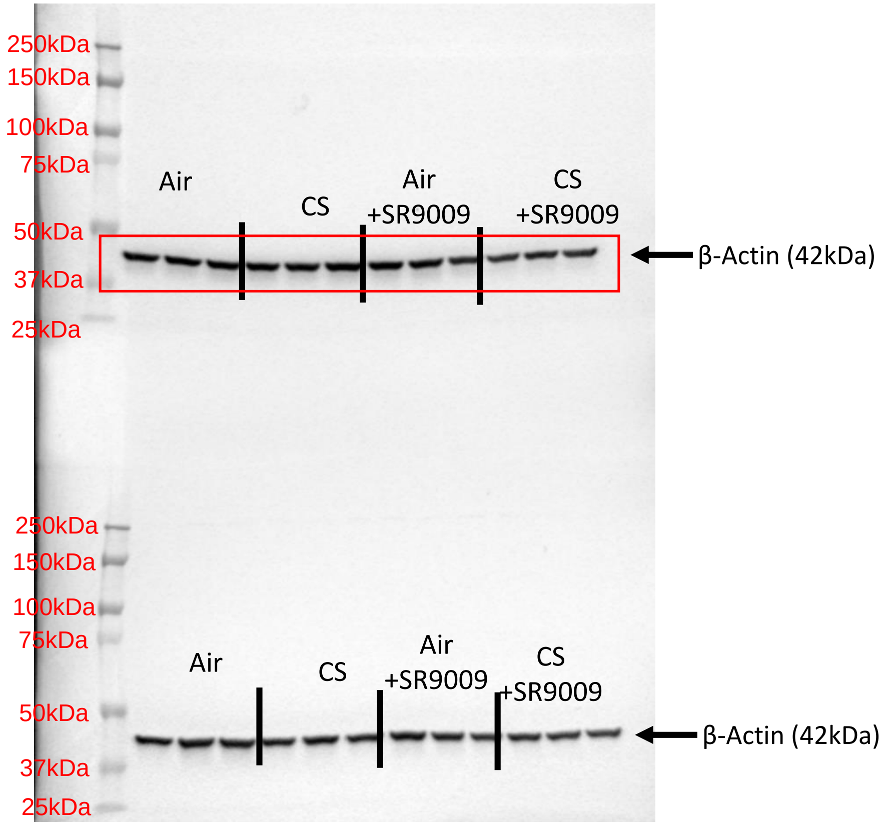

Only bands in red square represented in final figures, others were used in data analysis

Full unedited gel for Fig S9: REVERBα and β-Actin

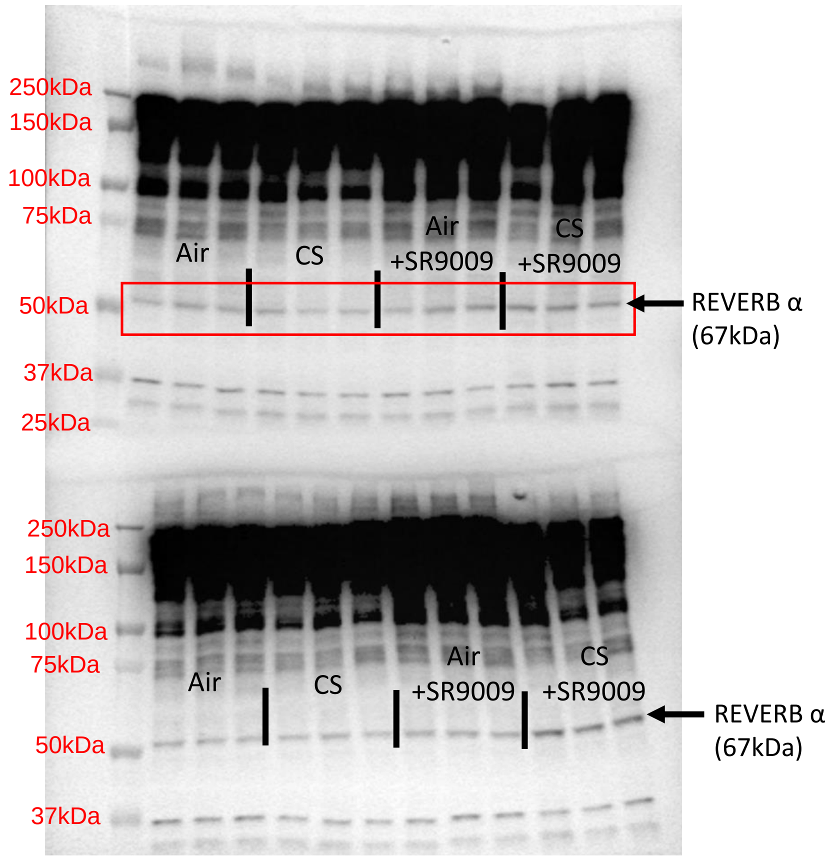

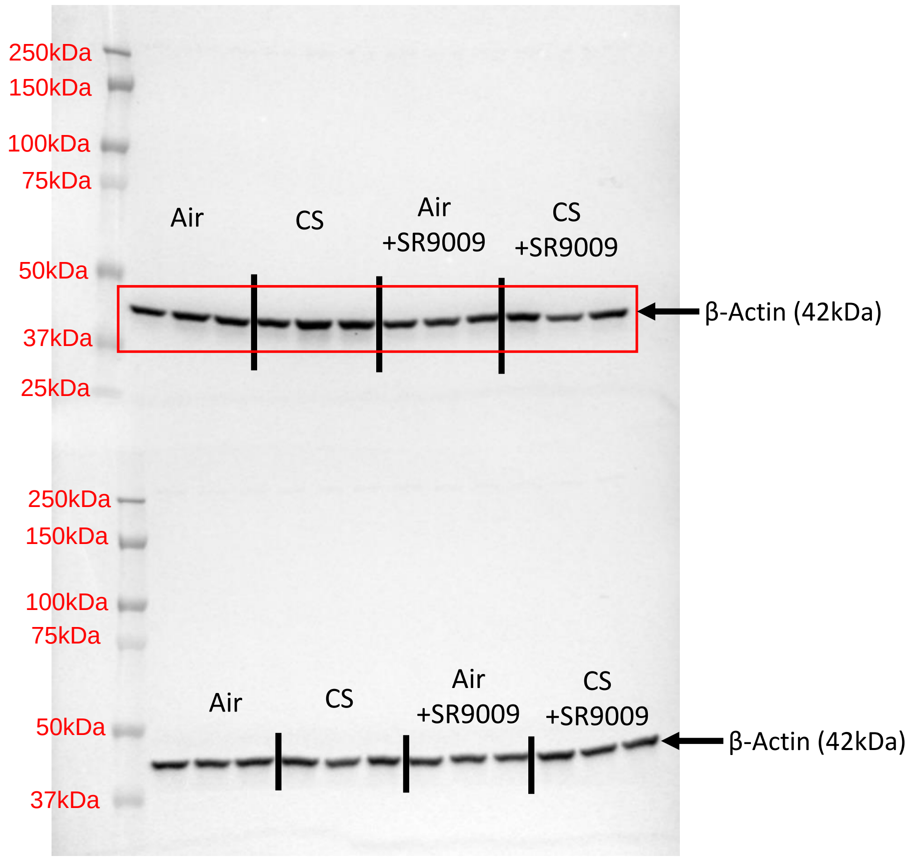

Only bands in red square represented in final figures, others were used in data analysis
